# Differential gene expression in a tripartite interaction: *Drosophila*, *Spiroplasma* and parasitic wasps

**DOI:** 10.7717/peerj.11020

**Published:** 2021-03-04

**Authors:** Victor Manuel Higareda Alvear, Mariana Mateos, Diego Cortez, Cecilia Tamborindeguy, Esperanza Martinez-Romero

**Affiliations:** 1 Centro de Ciencias Genómicas, Universidad Nacional Autónoma de México, Cuernavaca, Morelos, México; 2 Department of Ecology and Conservation Biology, Texas A&M University, College Station, TX, USA; 3 Department of Entomology, Texas A&M University, College Station, TX, USA

**Keywords:** Metatranscriptome, Spiroplasma, Parasitic wasp, Protection, Immunity, Drosophila, Toxins

## Abstract

**Background:**

Several facultative bacterial symbionts of insects protect their hosts against natural enemies. *Spiroplasma poulsonii* strain *s*Mel (hereafter *Spiroplasma*), a male-killing heritable symbiont of *Drosophila melanogaster*, confers protection against some species of parasitic wasps. Several lines of evidence suggest that *Spiroplasma*-encoded ribosome inactivating proteins (RIPs) are involved in the protection mechanism, but the potential contribution of the fly-encoded functions (e.g., immune response), has not been deeply explored.

**Methods:**

Here we used RNA-seq to evaluate the response of *D. melanogaster* to infection by *Spiroplasma* and parasitism by the *Spiroplasma*-susceptible wasp *Leptopilina heterotoma*, and the *Spiroplasma*-resistant wasp *Ganaspis* sp. In addition, we used quantitative (q)PCR to evaluate the transcript levels of the *Spiroplasma*-encoded Ribosomal inactivation protein (RIP) genes.

**Results:**

In the absence of *Spiroplasma* infection, we found evidence of *Drosophila* immune activation by *Ganaspis* sp., but not by *L. heterotoma*, which in turn negatively influenced functions associated with male gonad development. As expected for a symbiont that kills males, we detected extensive downregulation in the *Spiroplasma*-infected treatments of genes known to have male-biased expression. We detected very few genes whose expression patterns appeared to be influenced by the *Spiroplasma-L. heterotoma* interaction, and these genes are not known to be associated with immune response. For most of these genes, parasitism by *L. heterotoma* (in the absence of *Spiroplasma*) caused an expression change that was at least partly reversed when both *L. heterotoma* and *Spiroplasma* were present. It is unclear whether such genes are involved in the *Spiroplasma*-mediated mechanism that leads to wasp death and/or fly rescue. Nonetheless, the expression pattern of some of these genes, which reportedly undergo expression shifts during the larva-to-pupa transition, is suggestive of an influence of *Spiroplasma* on the development time of *L. heterotoma*-parasitized flies. One of the five RIP genes (RIP2) was consistently highly expressed independently of wasp parasitism, in two substrains of *s*Mel. Finally, the RNAseq data revealed evidence consistent with RIP-induced damage in the ribosomal (r)RNA of the *Spiroplasma*-susceptible, but not the *Spiroplasma*-resistant, wasp. Acknowledging the caveat that we lacked adequate power to detect the majority of DE genes with fold-changes lower than 3, we conclude that immune priming is unlikely to contribute to the *Spiroplasma*-mediated protection against wasps, and that the mechanism by which *Ganaspis sp*. resists/tolerates *Spiroplasma* does not involve inhibition of RIP transcription.

## Introduction

During their life cycle, insects face a large diversity of natural enemies such as predators and parasites, as well as infections by bacteria, fungi, and viruses. Although insects rely on an immune system to overcome these infections (reviewed in Hillyer, 2016), parasites and pathogens have evolved counter defenses. In this arms race, many insects have allied with symbiotic bacteria to fight against parasites. Extensive evidence of such defensive symbioses has been accrued over the last ~17 years (Oliver & Perlman, 2020).

Three models of classical ecology can be adapted to explain protection of bacteria against parasites (Gerardo & Parker, 2014). **Exploitation competition** occurs when the symbiont and the parasite compete for a limiting resource (e.g., *Wolbachia* and vectored-viruses compete for cholesterol; Caragata et al., 2013). **Apparent competition** can occur when the symbiont activates (“primes”) the immunity of the host, and thus indirectly interferes with the parasite (e.g., *Wigglesworthia* in *Glossina* against trypanosomes; Wang et al., 2009). Finally, **interference competition** can occur when the symbiont produces a compound (e.g., a toxin) that limits the success of the parasite (e.g., *Hamiltonella defensa* in aphid insects, Oliver et al., 2003; Brandt et al., 2017). One or more of these mechanisms can occur in concert, as suggested for the interactions between flies in the genus *Drosophila* and heritable bacteria in the genus *Spiroplasma*, where these endosymbionts protect the host against parasitic wasps or nematodes (Jaenike et al., 2010; Xie, Vilchez & Mateos, 2010).

The association between *Drosophila melanogaster* and its naturally occurring heritable facultative symbiont *Spiroplasma poulsonii* (*s*Mel) has emerged as a model system to study the evolutionary ecology and mechanistic bases of both defensive mutualisms (Jaenike et al., 2010; Xie, Vilchez & Mateos, 2010) and reproductive parasitism (i.e, male-killing) (Cheng et al., 2016; Harumoto et al., 2016). The presence of *S. poulsonii* in *Drosophila* larvae prevents the successful development of several species of parasitic wasps, including *Leptopilina heterotoma* (Xie, Vilchez & Mateos, 2010; Xie et al., 2014; Haselkorn & Jaenike, 2015; Mateos et al., 2016; Paredes et al., 2016). Parasitism by *L. heterotoma* in the presence of *Spiroplasma* results in no wasp survival, and in variable survival of *D. melanogaster* (range: <1 to 40%). Other factors that affect fly survival are species and strain of *Drosophila*, wasp, and *Spiroplasma*, as well as experimental conditions, including temperature (Mateos et al., 2016; Jones & Hurst, 2020a, 2020b; Corbin et al., 2021). Wasp species that are negatively affected by *Spiroplasma* are referred as “*Spiroplasma*-susceptible”. Several species of *Drosophila* parasitoids are unaffected by the presence of *Spiroplasma* in the host, such as *Ganaspis* sp. (strain G1FL), and are referred to “*Spiroplasma*-resistant” (Mateos et al., 2016).

Research into *Spiroplasma*-mediated protection against wasps has revealed *L. heterotoma* wasp embryos manage to hatch into first instars and achieve some growth, which is subsequently stalled (Xie, Vilchez & Mateos, 2010; Xie et al., 2014; Paredes et al., 2016). Evidence consistent with competition for lipids (i.e., exploitative competition) between *Spiroplasma* and the developing wasp has been reported for the wasp *Leptopilina boulardi* (Paredes et al., 2016).

Regarding the role of interference competition, the genomes of several *Spiroplasma* strains, including *s*Mel, encode genes with homology to Ribosomal inactivation proteins (RIPs; Ballinger & Perlman, 2017). RIP proteins, which are produced by different plants and bacteria (e.g., ricin and Shiga toxin, respectively), cleave a specific adenine present within a highly conserved (i.e., in all eukaryotes) motif of the large ribosomal subunit (28S rRNA), leading to inactivation of the ribosome and inhibition of protein translation (reviewed in Stirpe, 2004).

Damage consistent with RIP activity (hereafter referred to as depurination) has been detected in a *Spiroplasma*-susceptible nematode (Hamilton et al., 2015) and in two *Spiroplasma*-susceptible wasps (the larval parasitoids *L. boulardi* and *L. heterotoma*), but not in a *Spiroplasma*-resistant wasp that oviposits on fly pupae (Ballinger & Perlman, 2017). Although the above studies suggest that competition for nutrients and RIP activity are involved in the *Spiroplasma*-mediated mechanism that causes wasp death, they have not demonstrated that the above mechanisms alone or in combination are necessary and sufficient, and alternative mechanisms, including immune priming, have not been ruled out.

In response to wasp parasitism, *D. melanogaster* mounts an immune response characterized by proliferation of blood cells also known as hemocytes. Plasmatocytes are the first cells to attach to the foreign egg followed by lamellocytes which form successive layers; both types of hemocytes consolidate around the wasp egg, forming a capsule. The inner cells of the capsule produce melanin and release free radicals into the capsule, killing the wasp (Russo, 1996; Carton, Poirié & Nappi, 2008). Wasps have evolved a diverse array of strategies that counter the fly-encoded defense (Schlenke et al., 2007; Mortimer et al., 2013). Whether or not *Spiroplasma* contributes to enhancing the fly-encoded defense against wasps has not been extensively investigated. To date only one study has examined the possible influence of *Spiroplasma* on fly-encoded immunity against wasps (Paredes et al., 2016). Their results revealed no effect of *Spiroplasma* on the number of hemocytes in flies parasitized by *L. boulardi*. Whether *Spiroplasma* influences this or other aspects of fly-encoded immunity against other wasps has not been examined.

Herein, we used an RNA-seq approach to evaluate the transcriptomic response of *D. melanogaster* during interactions involving *Spiroplasma* and two wasps that are generalists of the genus *Drosophila*: the *Spiroplasma*-susceptible *L. heterotoma* and the *Spiroplasma*-resistant *Ganaspis* sp. In addition, we evaluated the effect of wasp parasitism on the expression of *Spiroplasma* RIP genes in two closely related substrains of *s*Mel, which have similar genomes (Gerth et al., 2021), but confer different levels of overall protection against *L. boulardi* (two strains tested) and one of two strains of *L. heterotoma* (Jones & Hurst, 2020b).

## Methods

### Insect and *S. poulsonii* strains

The transcriptomic experiments were performed on *D. melanogaster* flies (strain Canton S), which naturally harbor *Wolbachia* (Riegler et al., 2005). In a failed attempt to remove *Wolbachia*, several generations prior to the experiments, we treated these flies for three consecutive generations with tetracycline, followed by several generations with no antibiotics. Flies were reared in a Standard cornmeal medium (recipes in Supplemental Methods 1) at 25 °C, with a dark:light 12 h-cycle. Canton S flies were artificially infected with *Spiroplasma poulsonii* strain *s*Mel-BR (original isofemale line “Red42” from Brazil; Montenegro et al., 2005), via hemolymph transfer (as in Xie, Vilchez & Mateos, 2010) at least three generations before initiating the experiment. As *s*Mel-BR is a male-killer, the *Spiroplasma-*infected strain was maintained by addition of *Spiroplasma*-free males (Canton S strain) every generation.

The strains of wasps used were: the *Spiroplasma*-susceptible *Leptopilina heterotoma* strain Lh14 (Schlenke et al., 2007; voucher USNMENT01557081; hereafter “Lh”); and the all-female *Spiroplasma*-resistant *Ganaspis* sp. strain G1FL (Mortimer et al., 2013; voucher USNMENT01557080; also known as “drop_ Gan_sp53” in the *Drosophila* parasitoid database; Lue et al., 2021; hereafter “Gh”). Wasps were reared using second instar Canton S *Spiroplasma*-free larvae. These wasp strains are naturally infected with one or more *Wolbachia* strains (Wey et al., 2020).

For the qPCR assays, we used *Wolbachia*-free Oregon R flies to which *s*Mel-BR or *s*Mel-UG (original isofemale line from Uganda, Pool, Wong & Aquadro, 2006), had been artificially transferred at least three generations prior. These flies were maintained by matings with *Spiroplasma*-free Oregon R males under the same environmental conditions as the Canton S background flies, but in an opuntia-banana food medium (recipes in Supplemental Methods 1).

### Wasp exposure

**Figure 1:**
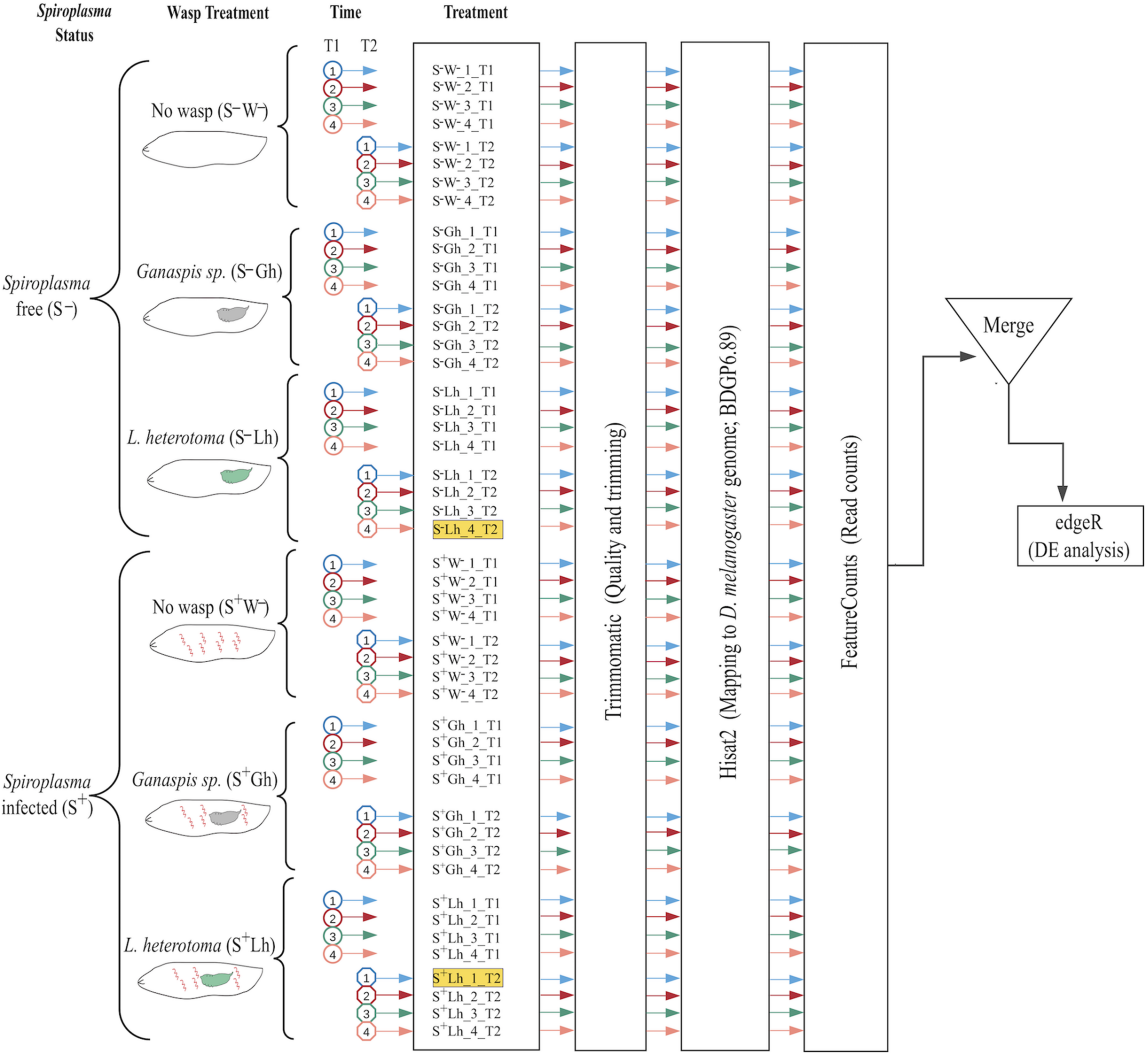
Experimental design and bioinformatic workflow to detect differentially gene expression in *D. melanogaster*. Treatments are firstly split by *Spiroplasma* infection status: S^–^ = *Spiroplasma*-free; and S^+^ = *Spiroplasma*-infected. Treatments are then split by Wasp Treatment: W^–^ = No wasp, Lh = exposed to *Leptopilina heterotoma*; and Gh = exposed to *Ganaspis* sp. Experiments were performed at 24 (T1) and 72 h (T2) post-wasp attack (PWA). For each treatment, four replicates were generated. The two samples highlighted in yellow (S^–^Lh_4_T2 and S^+^Lh_1_T2) were excluded from the analysis (see main text). Each cartoon depicts a fly larva (white) with or without *Spiroplasma* (red spirals) and wasp larva: green = *L. heterotoma* (Lh); and grey = *Ganaspis* sp. (Gh).

To examine the effect of the interaction of *Spiroplasma* and wasp on the transcriptome of *Drosophila* (and of *Spiroplasma*), we compared treatments with the presence and absence of *Spiroplasma* (*s*Mel-BR) and one of the two wasp species at two different time points (Fig. 1). This experimental design resulted in a combination of twelve treatments; six treatments per time point. For each replicate, parental flies (approximately ten females and ten males) were set up in oviposition vials in the evening for overnight oviposition. Parental flies were removed the next morning. One day later, ~30 second-instar *D. melanogaster* larvae were carefully collected and transferred to a Petri dish (60 mm diameter) containing cornmeal food medium. In replicates assigned to a wasp treatment, five male and six female wasps of the corresponding species (*L. heterotoma* or *Ganaspis* sp.) were added to the Petri dish and allowed to oviposit for ~5 h. All female wasps had been previously allowed to oviposit on *D. melanogaster* fly larvae for ~5 h. The purpose of this “training” is to ensure that the wasps are experienced at oviposition prior to the experiment. All Petri dishes were covered, but a small hole was opened (with a hot needle) to allow for gas exchange, and/or through which wasps could feed on a piece of cotton wool soaked in 1:1 water:honey mix that was placed outside the dish. To collect RNA, larvae were retrieved from each Petri dish at either 24 h (T1) or 72 h (T2) post-wasp attack (PWA) (i.e., one or three days after wasps were removed, respectively). To ensure sufficient material for RNA-seq, for each replicate we pooled 11–30 larvae at the 24 h time-point, and 13–23 larvae at the 72 h time point, with the exception of one replicate (only three larvae, which we subsequently removed; see “Results”). Both wasp species were embryos at T1 and larvae at T2. Fly larvae from the same Petri dish were pooled into a single RNA extraction tube (i.e., a replicate).

From each Petri dish in the wasp-exposed treatments, a subsample of fly larvae was used to verify wasp parasitism rate as follows. First, at the time of larvae collection for RNA extraction, five fly larvae per replicate were dissected under the microscope and subsequently discarded. If all five larvae contained at least one wasp egg or larva (i.e., 100% wasp oviposition rate), the replicate was retained and processed. If one or more fly larvae did not contain a wasp larva (or embryo), then five additional fly larvae were examined for wasp presence. We discarded replicates where more than two larvae were found unparasitized. All collections for RNA were performed in the afternoon-evening, and collected larvae were quickly placed in an empty microtube for processing.

### RNA extraction

Preliminary experiments with RNAlater Stabilization Solution revealed that the larvae did not die immediately and appeared to melanize. Therefore, collected larvae were either immediately subjected to the total RNA extraction procedure, or frozen at −80 °C for subsequent extraction. Total RNA was extracted using the Trizol (Invitrogen, Carlsbad, CA, USA) method. Each sample consisted of a pool of *Drosophila* larvae that were homogenized by hand with a sterile plastic pestle in Trizol reagent. The Trizol isolation method was performed following the manufacturer’s protocol but it was stopped at the 70% ethanol wash step. The total RNA pellet in ethanol was submitted to the Texas AgriLife Genomics and Bioinfomatics Services facility for completion of the RNA isolation procedure, assessment of RNA quality and quantity (using Fragment Analyzer; Agilent, Santa Clara, CA, USA), library preparation, sequencing, and demultiplexing.

### Library preparation and sequencing

Total RNA was subjected to removal of ribosomal RNA from eukaryotes and prokaryotes with the RiboZero Epidemiology kit (Illumina, San Diego, CA, USA). The TruSeq stranded kit (Illumina) was then used to prepare the library for sequencing with Illumina (125 bp Single End “HighSeq 2400v4 High Output”).

### Bioinformatic analysis

Quality and presence of adapters was evaluated with FASTQC (Andrews, 2010), followed by a filtering/trimming procedure with Trimmomatic v.0.36 (Bolger, Lohse & Usadel, 2014), using ILLUMINACLIP:/adapters.fasta:2:30:10 LEADING:3 TRAILING:3 SLIDINGWINDOW:4:15 MINLEN:36. To examine differential expression (DE) of *D. melanogaster* genes, trimmed reads were mapped with Hisat2 v.2.0.2-beta (Kim, Langmead & Salzberg, 2015), using—rna-strandness R option. Treatments parasitised by *L. heterotoma* were mapped to an index composed by *D. melanogaster* genome (ensembl version BDGP6) plus *L. heterotoma* genome, reference VOOK00000000. Treatments parasitised by *Ganaspis* sp. were only mapped to *Drosophila*, because there is not an available genome for this strain of *Ganaspis* sp. The resulting *D. melanogaster* mapped reads were quantified using featureCounts from the Subread package v1.6.2 (Liao, Smyth & Shi, 2014), using the following parameters: -s 2, -t exon, -g gene_id. Differential gene expression was assessed for pairs of treatments in R (R core Team) with edgeR v 3.24.3 package (Robinson, McCarthy & Smyth, 2009). For each pairwise treatment comparison, genes with counts <1 cpm for all replicates were discarded. Only genes with absolute 2LogFC >= 0.58 and FDR < 0.05 were considered differentially expressed (DE). The Robust parameter (robust = TRUE) of edgeR was implemented to minimize false positive DE genes. The gene ids corresponding to the DE genes were loaded (before May 2020) into Flymine, (an integrated database for *Drosophila* genomics, Lyne et al., 2007) available at https://www.flymine.org/flymine/begin.do. This platform outputs gene names plus other information such as Gene Ontology (GO), enrichment of pathways, tissue expression and protein domains. Expression patterns of individual genes were obtained from flybase2.0 (Thurmond et al., 2019) available at https://flybase.org/. Venn diagrams used to detect exclusive genes in the interactions were generated using the web-tool available at http://bioinformatics.psb.ugent.be/webtools/Venn/.

To measure *Spiroplasma* gene expression, the trimmed reads from the *Spiroplasma*-infected treatments were subjected to kallisto v.0.43.1 (Bray et al., 2016), using the genome of *S. poulsonii s*Mel-UG (GCF_000820525.2), as reference. Count tables obtained by kallisto were used to detect DE genes using edgeR. Expression of RIP genes in the transcriptome was obtained from this table using trimmed mean of *M*-values (TMM) normalized counts. Heatmaps of expression patterns were generated with the R package pheatmap v 1.0.12.

### Power analyses for DE

To identify limitations on the detection of *Drosophila* DE genes, we performed a statistical power analysis with the R package RNASeqPower v.1.22.1 (Hart et al., 2013). Input parameters such as coverage and the biological coefficient of variation (BCV) to run RNASeqPower are reported in Table S1. Coverage was calculated from our data (Table S2). The BCV was determined for each pairwise comparison and represents the square root of the common dispersion that is reported by edgeR.

### Analyses of depurination signal in the sarcin-ricin loop (SRL) of the 28S rRNA of wasps and flies

Ribosomal inactivation proteins toxins remove a specific adenine present in the sarcin-ricin loop (SRL) of the 28S rRNA leaving an abasic site (i.e., the backbone remains intact). When a reverse transcriptase encounters an abasic site, it preferentially adds an adenine in the nascent complementary DNA strand. This property, which results in an incorrect base at the RIP-depurinated site in the cDNA and all subsequent PCR amplification steps, has been used to detect evidence of RIP activity in any procedure that relies on reverse transcription such as RNA-seq or reverse-transcription qPCR (Hamilton et al., 2015).

To examine whether a signal of depurination consistent with RIP activity was detectable in wasp-derived sequences, we mapped RNA-seq data to a reference sequence file comprised of the 28S rRNA sequences of the wasps and of *D. melanogaster* using Bowtie2 v2.3.5 (Langmead & Salzberg, 2012), with default options. Only sequences that mapped to the wasp 28S rRNA were retained (Dataset S1). To visualize and count the shift from A to T (or other bases), the retained reads were mapped again to the 28S rRNA of wasp in Geneious v.11.1.2 (Biomatters Inc., Newark, NJ, USA; “low sensitivity mode”; maximum gap size = 3; iterate up to 25 times, maximum mismatches per read 2%). The number of reads containing each of the four bases or a gap at the target site were counted by selecting the position at all the reads to be counted, and recording the counts reported by Geneious under the “Nucleotide Statistics” option (gapped reads were excluded from counts). Reads were counted only if they fully covered a specific part of the 28S loop sequence (TACG**A**GAGGAACC). The bold-faced adenine represents the site of RIP depurination. Replicates with fewer than 10 mapped reads were discarded, Table S3 and Fig. S11. Statistical analysis was conducted in R v 4.02 (R core Team) using a Bayesian generalized linear model (bayesglm function in “arm” package), due to the presence of zeros (no depurination) in some treatments. Using the above strategy, raw sequences were also mapped to the full sequence of the 28S rRNA of *D. melanogaster*, and depurination was evaluated. Due to the high number of ribosomal sequences that align to the 28S rRNA of *Drosophila*, only subsets of 1 million of sequences were analyzed (Table S4).

### Expression of *Spiroplasma* RIPs

To verify the RIP expression patterns inferred from the transcriptome (see “Results”) and to examine whether they were consistent between substrains of *s*Mel, we used qPCR on a new set of treatments. We followed the “Wasp exposure” methodology (described above), but used *Wolbachia-*free *D. melanogaster* (Oregon R) harboring the *S. poulsonii* strain *s*Mel-BR or *s*Mel-UG. Five larvae per treatment (parasitized or not by *L. heterotoma* or *Ganaspis* sp.), were collected at 24 and 72 h PWA, flash frozen in liquid nitrogen and homogenized by hand with a pestle. Total RNA was extracted with the All prep DNA/RNA mini kit (Qiagen, Germantown, MD, USA). 1µg of total RNA was used to synthesize cDNA using superscript II reverse transcriptase (Invitrogen, Carlsbad, CA, USA), following manufacturer’s procedures. cDNA was used as a template for qPCRs, performed on a CFX96 detection system (Bio-Rad, Hercules, CA, USA). The mix contained 5 µl of iTaq Universal SYBR Green Supermix (Bio-Rad, Hercules, CA, USA), 2.5 µl water, 2 µl cDNA, and 0.25 µl of each primer (stock solution at 10 µM). Primer sequences and efficiencies for RIP2, RIP3-5 and rpoB were taken from (Ballinger & Perlman, 2017). For RIP1, we designed and used the following primers Forward: 5′- AATCAGAGGGGCATTAGCTC-3′ Reverse 5′-CTTCGCTTGTGGTTCTTGAT-3′, efficiency = 0.995. Although Ballinger & Perlman (2017) reported a primer pair targeted at RIP1, this primer pair matches a fragment of RIP2 instead.

Relative expression was calculated using efficiency-corrected Ct values using (Ct × (Log(efficiency)/Log(2))) formula. DeltaCt was calculated as Ct-rpoB minus Ct-RIPx (Dataset S2). We used JMP Pro v.15 (SAS, Cary, NC, USA) to fit a full factorial Generalized Regression (Normal distribution) model. The response variable was delta Ct Value. The independent variables (all fixed and categorical) were: RIP gene (“RIP”: RIP1, RIP2, RIP3-5), Wasp Treatment (No wasp, Lh and Gh), *Spiroplasma* strain (Brazil or Uganda) and Time Point (24 or 72 h). Significant effects and interactions were explored with Tukey HSD tests with Least Square Means Estimates (Supplemental File S1).

### Data availability

All raw reads generated in this project have been submitted to NCBI under BioProject PRJNA577145 and BioSample SAMN13020352. Count tables for *D. melanogaster* and *Spiroplasma* are in Dataset S3. Command lines used to run bioinformatic analyses are available in Supplemental Methods 2. Raw Ct values from the qPCR analyses are in Dataset S2. Output results of edgeR for each pairwise treatment comparison are in Dataset S6.

## Results

To examine the effect of *Spiroplasma* on *D. melanogaster* gene expression under wasp parasitism, we generated 12 RNA-seq treatments (Fig. 1), with an average of 47 million quality single-end reads per sample, ~90% of these reads mapped to the *D. melanogaster* genome. A fraction of these reads mapped to ribosomal sequences of the host, which is an indication of incomplete ribodepletion (Table S2). The power analyses revealed that we should have sufficient power to detect 91–100% of differentially expressed (DE) genes with fold changes = 4; 75–99% of DE genes with fold-changes= 3; 38–80% of DE genes with fold changes = 2, and 16–37% of DE genes with fold changes = 1.5 (Table S1; Fig. S1).

The multidimensional scaling (MDS) plot of all treatments at 24 h post-wasp attack (PWA; T1) did not reveal any particular grouping by treatment (Fig. S2). At the 72 h PWA time point (T2), however, the treatments separated at the first dimension by presence/absence of *Spiroplasma* (Fig. S3). This plot allowed us to detect two replicates that we deemed outlier and decided to exclude from further analyses (see Fig. 1). Replicate “S^**–**^Lh_4_T2”, which belonged to a *Spiroplasma*-free treatment, grouped with the *Spiroplasma*-infected treatments. Absence of *Spiroplasma* infection in replicate S^**–**^Lh_4_T2 was confirmed, as no reads mapped to the *Spiroplasma* genome. Thus, the grouping of replicate S^**–**^Lh_4_T2 with the *Spiroplasma*-infected treatments (which should lack males) could be explained by a lack of males; a scenario that is likely given that this replicate only had three larvae (i.e., assuming equal sex ratios in the *Spiroplasma*-free flies, the probability of sampling all females is 0.5^3^ = 12.5%). Concerning replicate “S^+^Lh_1_T2”, its expression pattern included upregulation of numerous genes associated with immune response. Consequently, we suspected that this particular replicate likely contained one or more larvae infected by a pathogenic bacterium.

In the next sections, we first describe the response of *D. melanogaster* to parasitism by the wasps (*L. heterotoma* or *Ganaspis* sp.) in the absence of *Spiroplasma*, followed by the fly response to the sole presence of *Spiroplasma*. Finally, taking these results into account, we examine the response of *Drosophila* during the *Spiroplasma*-wasp interaction. Due to expected power limitations, we acknowledge that there may be relevant *Drosophila* genes to the interaction among fly, wasp and *Spiroplasma*, that were not detectable as DE in our experiments, particularly if their fold-changes are lower than ~3 and/or their expression level is low (Oshlack & Wakefield, 2009).

### Response of *D. melanogaster* to *L. heterotoma* parasitism in the absence of *Spiroplasma*

**Figure 2:**
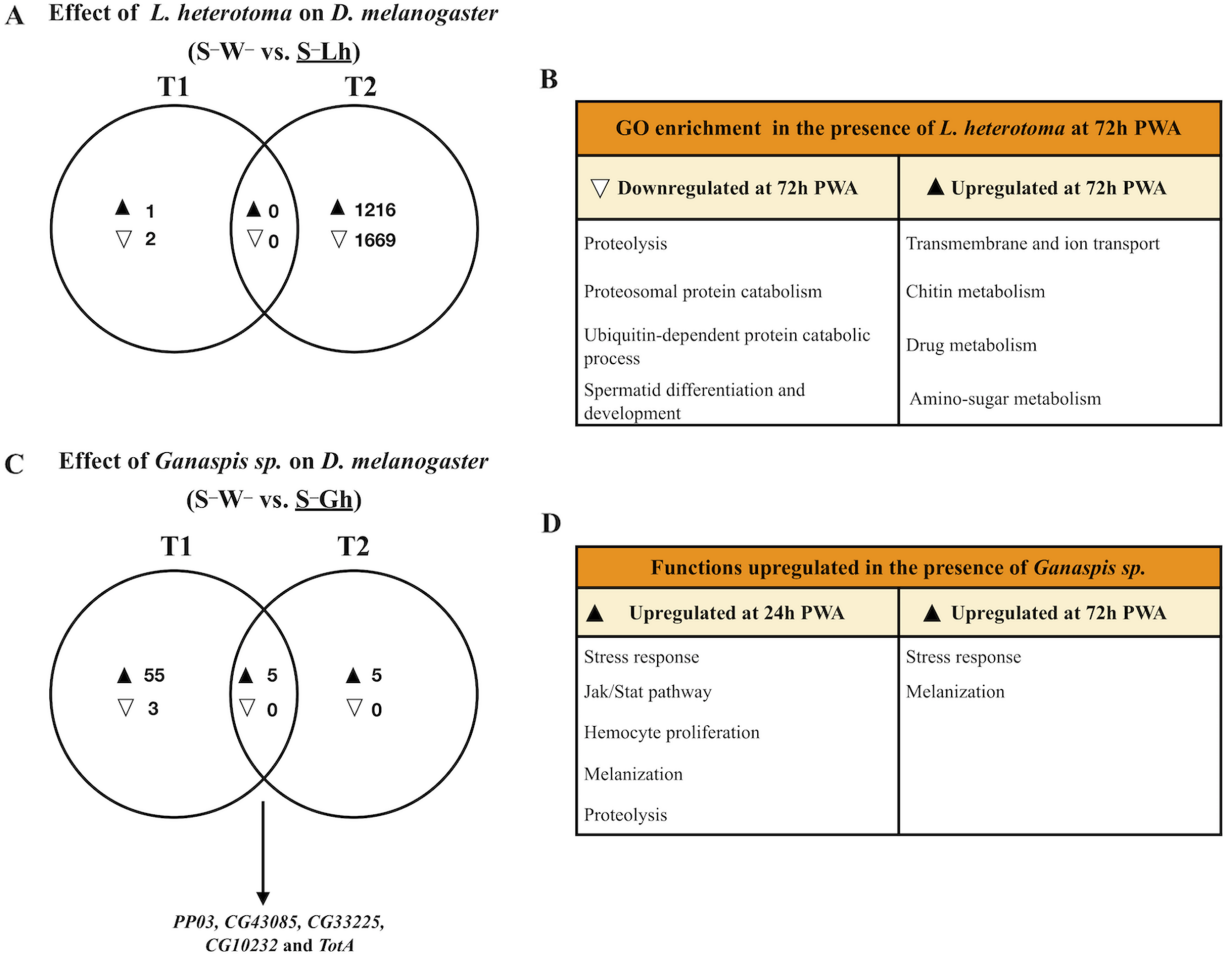
Venn diagrams and enriched functions or Gene Ontology (GO) categories during wasp parasitism in the absence of *Spiroplasma*. Differentially expressed genes of *D. melanogaster* at time points T1 and T2 (24 and 72 h post-wasp attack (PWA), respectively) by (A) and (B) *L. heterotoma* or (C) and (D) *Ganaspis* sp. Black and white triangles indicate up- and downregulation, respectively, in the treatment that is underlined. (B) Some of the most enriched GO categories induced by *L. heterotoma*. (D) Functions induced by *Ganaspis* sp. were manually assigned based on gene annotation of DE genes. (C) The arrow in this panel indicates the five genes in the T1 and T2 intersection. S^–^W^–^ = *Spiroplasma*-free and wasp-free; S^–^Lh = *Spiroplasma*-free (S^–^) and parasitized by wasp *L. heterotoma* (Lh); S^–^Gh = *Spiroplasma*-free (S^–^) and parasitized by wasp *Ganaspis* sp. (Gh).

Parasitism by *L. heterotoma* at T1 (24 h) post-wasp attack (PWA), did not have a large effect on *D. melanogaster* gene expression, as only one gene (*thor*) was upregulated, whilst two genes, *Hml* and *mt:ATPase6*, were downregulated (Fig. 2A; Dataset S4). In contrast, at T2 (72 h PWA), 1216 genes were up- and 1669 down-regulated (Fig. 2A; Dataset S5). Of the 1216 upregulated genes at T2, 818 grouped into 32 GO enriched categories (Fig. 2B); of which transport was the most enriched. A pathway analysis revealed that upregulated genes are involved with energy generation pathways, such as lipid metabolism and citric acid cycle (Dataset S5). Of the 1669 downregulated genes, 945 grouped into 25 GO categories, of which proteolysis was the most enriched (Fig. 2B; Dataset S5). Unexpectedly, a subset of 67 downregulated genes belongs to GO categories related to spermatogenesis, suggesting that *L. heterotoma* may interfere with male gonad development (further discussed below).

### Response of *D. melanogaster* to *Ganaspis* sp. parasitism in the absence of *Spiroplasma*

In the presence of the wasp *Ganaspis* sp. (i.e., S^**–**^W^**–**^ vs. S^**–**^Gh), three genes were down- and 60 were up-regulated at T1 (Fig. 2C). The two downregulated genes are known to be expressed by hemocytes (*Peroxidasin* and *hemolectin*) (Irving et al., 2005), whereas the other is a small nucleolar RNA (*Uhg4*). Only one gene, *hemolectin*, was down-regulated by both *L. heterotoma* and *Ganaspis* sp. at T1 (Dataset S4). The sixty upregulated genes in the presence of *Ganaspis* sp. at T1 include genes that are known to be expressed preferentially by hemocytes such as *hemese*, *ItgaPS4, ItgaPS5*, *Scavenger receptor class C* (*CG3212*), one serpin (*CG6687*), a serine protease (*CG6639*), as well as one gene involved in hemocyte proliferation (*pvf2*) (Irving et al., 2005). One activator of the Jak Stat pathway, *upd3* and some effectors of this pathway, *TotA, tep1* and *tep2* (Agaisse & Perrimon, 2004), were also upregulated. Prophenoloxidase 3 (*PPO3)* and *yellow-f* genes, which are involved in the melanization process (Han et al., 2002; Dudzic et al., 2015), were also upregulated by *Ganaspis sp. PPO3* was highly upregulated (2 LogFC = 9). The complete list of DE genes and GO enrichment at T1 is provided in Dataset S4.

At T2, the presence of *Ganaspis* sp. induced upregulation of ten genes, but no genes were downregulated (Fig. 2C; Dataset S5). The upregulated genes included the stress response genes *TotA*, *TotB*, *TotC* and *victoria*, but also *PPO3*; log2FC of these genes ranged 3–10. Five genes were shared between the two time points (Fig. 2C). Two of them, *CG10232* and *CG33225* are predicted to be involved in proteolysis, and *CG43085* has no function or prediction assigned. The other two genes were *PPO3* and *TotA*; their log2FC were higher at T1 than at T2. In general, immune functions were upregulated by *Ganaspis* sp. parasitism at both time points (Fig. 2D).

### Response of *D. melanogaster* to *Spiroplasma*

**Figure 3:**
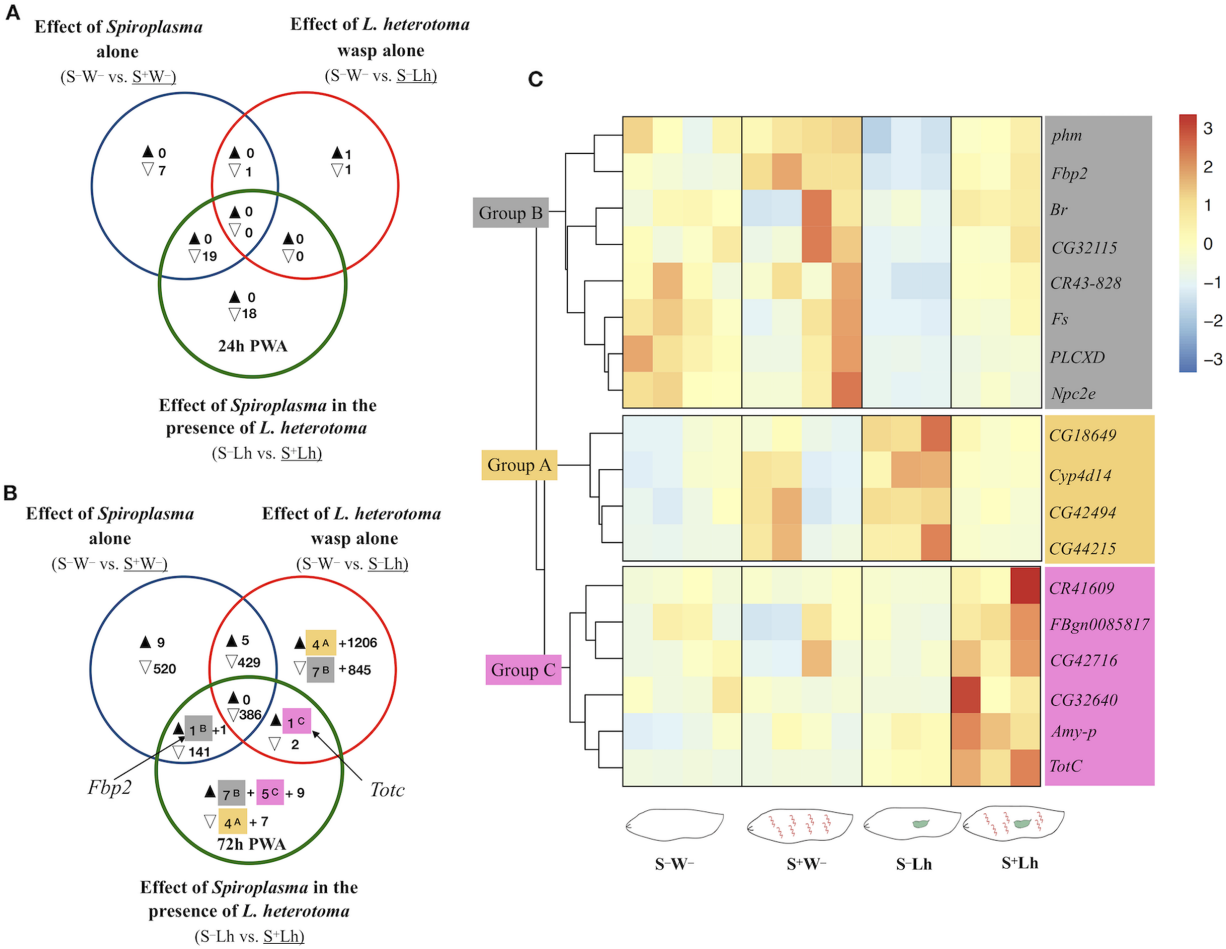
Patterns of differentially expressed genes of *Drosophila* in the *Spiroplasma*- *L. heterotoma* (Lh) interaction. Unique and shared number of DE genes in each of three treatment comparisons (superscript letters refer to groups defined below). S^–^W^–^ = *Spiroplasma*-free and wasp-free; S^+^W^–^ = *Spiroplasma*-infected (S^+^) and wasp-free (W^–^); S^–^Lh = *Spiroplasma*-free (S^–^) and parasitized by wasp *L. heterotoma* (Lh); S^+^Lh = Spiroplasma-infected (S^+^) and parasitized by wasp *L. heterotoma* (Lh). (A) Time-point 1 (T1): 24 h post-wasp attack (PWA). (B) Time-point 2 (T2): 72 h PWA. Black and empty triangles indicate up- and downregulation, respectively, in the treatment that is underlined. Blue sets = effect of *Spiroplasma* in the **absence** of wasps. Red sets = effect of wasp *L. heterotoma* in the **absence** of *Spiroplasma*. Green sets = effect of *Spiroplasma* in the **presence** of wasp *L. heterotoma*. (C) Heatmap of candidate genes involved in the *Spiroplasma* mediated protection mechanism (at T2 only); Z-score of edgeR TMM-normalized values are shown. Three main clusters are identified. Group A (yellow background) contains four genes exclusively downregulated in the green set, and exclusively upregulated in the red set. Group B (grey background) contains seven genes exclusively upregulated in the green set, and exclusively downregulated in the red set, plus *Fbp2* (upregulated in the blue and green sets). Collectively, we refer to Groups A and B as “restored” (see text). Group C (pink background) contains five genes exclusively upregulated in the green set, plus *TotC* (upregulated in the red and green sets). Group C genes had the highest expression level in the presence of *Spiroplasma* and Lh (S+Lh), whereas the other three treatments had levels similar to each other.

The sole presence of *Spiroplasma* (i.e., S^**–**^W^**–**^ vs. S^+^W^**–**^ comparison) at 24 h PWA did not reveal any upregulated genes, but 27 were downregulated (blue set in Fig. 3A). Twenty of 27 downregulated genes are reported as preferentially expressed in adult testis (Dataset S4). At 72 h PWA, the presence of *Spiroplasma* induced upregulation of 16 and downregulation of 1,476 genes (blue set it Fig. 3B). Only downregulated genes (692 of the 1,476) were assigned to one or more of 71 GO categories (Dataset S5). Some of these categories are related to the energy generation process such as oxidative phosphorylation, pyruvate metabolic process, and glycolytic process. Among the most enriched categories were male gamete generation and spermatogenesis, which is in agreement with the expected lack of males in S^+^W^**–**^ treatments. In addition, 1,333 of the 1,476 downregulated genes at T2 are classified as preferentially upregulated in fly testis. Furthermore, the cpm values of ~78% of these genes in all replicates of the S^+^W^**–**^ treatment were <1, implying very low expression levels. Therefore, as expected, a large number of the genes with lower expression in the *Spiroplasma* treatment, including the *roX1* and *roX2* (exclusively expressed in males, and part of the dosage compensation system; reviewed in Lucchesi & Kuroda, 2015), may simply reflect the absence of males. For the remaining downregulated genes that are not reported as having male-biased gene expression, it is not possible to determine whether they are *Spiroplasma*-responsive vs. sex-biased genes, because RNAseq studies comparing female vs. male larvae at this stage are lacking.

### *Drosophila* gene expression during *Spiroplasma–L. heterotoma* interaction

To explore if the presence of *Spiroplasma* influences gene expression of *D. melanogaster* during parasitism by *L. heterotoma*, we adopted the following strategy to identify genes whose expression was specifically influenced by the *Spiroplasma* X *L. heterotoma* interaction. We used a Venn diagram depicting differentially expressed genes from the following three pairwise treatment comparisons. Comparison S^**–**^W^**–**^ vs. S^+^W^**–**^ (blue sets in Fig. 3), represents the effect of *Spiroplasma* in the absence of any wasp. Comparison S^**–**^W^**–**^ vs. S^**–**^Lh (red sets), represents the effect of *L. heterotoma* in the absence of *Spiroplasma*. Comparison S^**–**^Lh vs. S^+^Lh (green sets) represents the effect of *Spiroplasma* in the presence of Lh. Genes that fell in the exclusive part of this green set were initially deemed as influenced specifically by the *Spiroplasma* X *L. heterotoma* interaction. These genes were further evaluated with heatmaps of expression levels at each replicate of the four relevant treatments (S^**–**^W^**–**^, S^+^W^**–**^, S^**–**^Lh and S^+^Lh). Genes that fell in the intersection of the green set with one or both of the blue and red sets, were also further evaluated with heatmaps. In general, the heatmaps allowed us to prioritize genes of interest based on their behavior in the context of the other treatments (see below), and based on relatively low among-replicate variation.

The S^**–**^Lh vs. S^+^Lh comparison at T1 yielded zero up- and 18 exclusively down-regulated genes (exclusive green set in Fig. 3A, Dataset S4). Ten of these genes code for small nucleolar RNAs (snoRNA). Five other genes (*fest*, *CG10063*, *SkpC*, *eIF4E3* and *cona*), albeit exclusively downregulated in the *Spiroplasma–L. heterotoma* interaction, seem to be influenced by *Spiroplasma* alone, based on the observation that these genes were downregulated by *Spiroplasma* alone at the later time point T2 (Dataset S5). Furthermore, *fest* and *eIF4E3* are upregulated in testis, and thus their downregulation may simply reflect absence of males.

The only gene-containing intersection (green+blue; Fig. 3A) at T1 had 19 genes, of which 16 are preferentially expressed in adult testis (Dataset S4). The heatmap of all genes in the intersection (Fig. S4), revealed that most of them had similarly low levels of expression in the two *Spiroplasma*-infected treatments (i.e., S^+^W^**–**^ and S^+^Lh), compared to the S^**–**^W^**–**^, which had the highest; a pattern that could be attributed to the absence of males in the *Spiroplasma* treatments. In turn, the S^**–**^Lh treatment exhibited an intermediate expression level (Fig. S4). In other words, expression levels among the four treatments were: S^**–**^W^**–**^ > S^**–**^Lh > S^+^W^**–**^ = S^+^Lh. We hereafter for simplicity we loosely refer to genes with such an expression pattern as “Lh-affected male gonad genes”.

At T2, 11 genes were exclusively downregulated in the presence of *Spiroplasma* and wasp (S^+^Lh) compared to only wasp (S^**–**^Lh); that is, in the exclusive green set (Fig. 3B; Dataset S5). Of these, four (identified as Group A with yellow highlight and superscript “A”) were exclusively upregulated in presence of wasp (S^**–**^Lh) compared to no wasp (S^**–**^W^**–**^) (exclusive red set in Fig. 3B; Dataset S5). Also at T2, 21 genes were exclusively upregulated in the S^+^Lh treatment compared to S^**–**^Lh (exclusive green set). Of these 21 genes, seven (assigned to “Group B”; superscript “B” and grey highlight) were exclusively downregulated in S^**–**^Lh compared to the *Spiroplasma*-free and wasp-free control (S^**–**^W^**–**^); that is, in the exclusive red set. Hereafter, we refer to genes in Groups A and B as “restored” because parasitism by *L. heterotoma* (S^**–**^Lh) increased and decreased (respectively) their expression with respect to the S^**–**^W^**–**^ control, but in the presence of *Spiroplasma* plus *L. heterotoma* (i.e., the S^+^Lh treatment), expression levels appear to return to those observed in the control (S^**–**^W^**–**^; Fig. 3C). In other words, *Spiroplasma* appears to “buffer” or counter the effects caused by the presence of *L. heterotoma*. Visual inspection of heatmaps of the remaining genes in the exclusive green set (not shown) or in the intersections with the green set (Figs. S5 and S6) revealed the following additional genes with expression patterns suggestive of an effect of the interaction of *Spiroplasma* and *L. heterotoma*. *Fbp2*, from the blue+green intersection, clustered with the Group B genes. *TotC*, from the red+green intersection, along with five genes exclusively upregulated in the green set, clustered into Group C (labeled with superscript “C” and pink highlight in Figs. 3B and 3C). Group C genes exhibit the highest expression level in the S^+^Lh treatment.

A heatmap of the remaining exclusive DE genes (green set, Fig. 3B) that do not belong to Groups A–C (nine up- and seven down-regulated, Fig. S13), did not show relevant expression patterns when compared to the other treatments and taking into account variation among replicates. Among these exclusively upregulated genes was the antimicrobial peptide *Attacin-C* (*AttC*; FC = 1.5); the only DE gene associated with an immune function. Finally, all of the genes in the red+blue+green intersection (386 genes) and most of the genes in the blue+green intersection (143 genes) at T2 (Fig. 3B), are enriched in male gonads (Dataset S5) and exhibited the expression pattern observed in the “Lh-affected male gonad genes” (Figs. S5 and S6).

### *Drosophila* gene expression during *Spiroplasma–Ganaspis* sp. interaction

**Figure 4:**
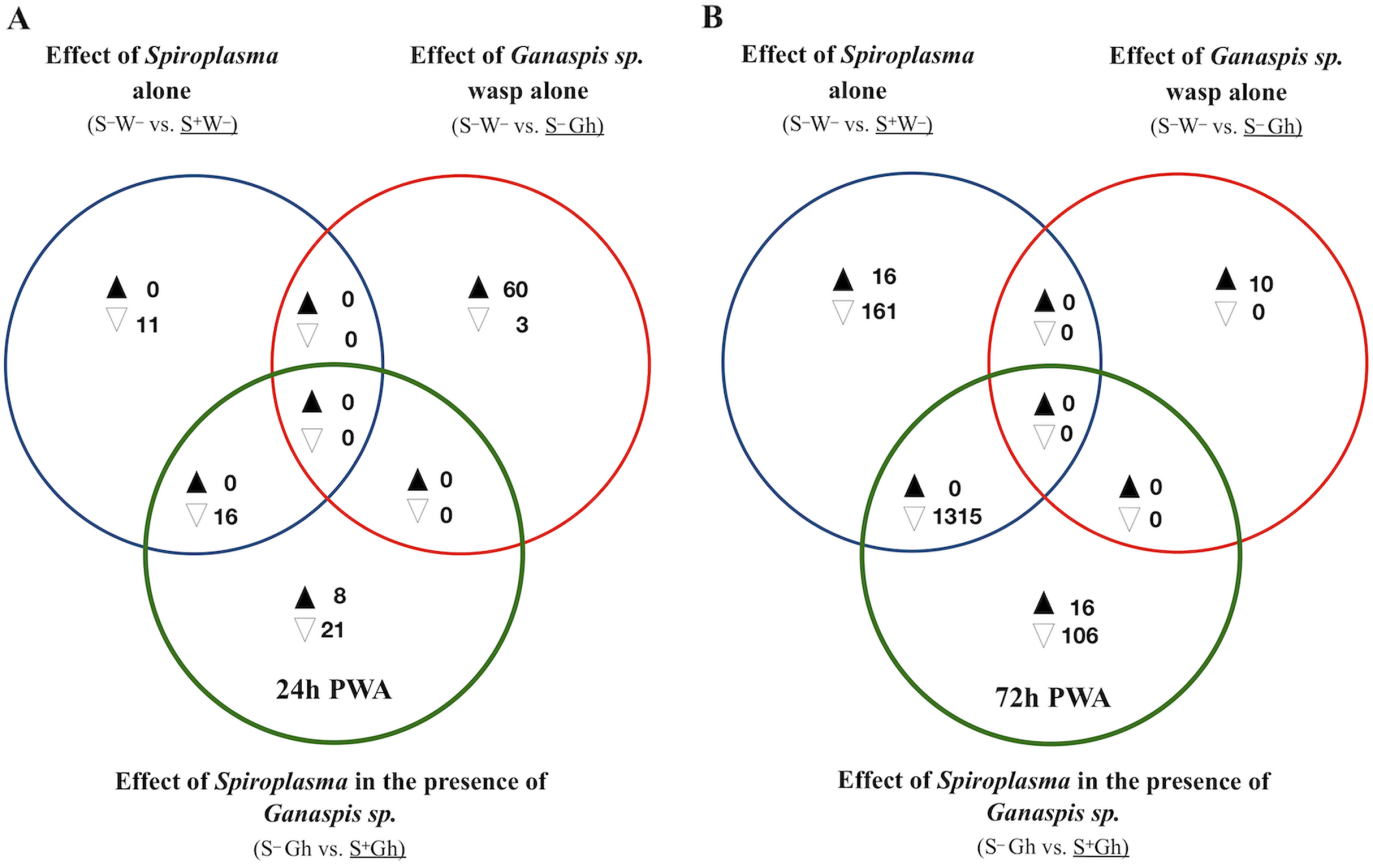
Patterns of differentially expressed genes of *Drosophila* in the *Spiroplasma*-*Ganaspis* sp. (Gh) interaction. Unique and shared number of DE genes in each of three treatment comparisons. S^–^W^–^ = *Spiroplasma*-free and wasp-free; S^+^W^–^ = *Spiroplasma*-infected (S^+^) and wasp-free (W^–^); S^–^Gh = *Spiroplasma*-free (S^–^) and parasitized by wasp *Ganaspis sp*. (Gh); S^+^Gh = *Spiroplasma*-infected (S^+^) and parasitized by wasp *Ganaspis* sp. (Gh). (A) Time-point 1 (T1): 24 h post-wasp attack (PWA). (B) Time-point 2 (T2): 72 h PWA. Black and white filled triangles indicate up- and downregulation, respectively, in the treatment that is underlined. Blue sets = effect of *Spiroplasma* in the **absence** of wasps. Red sets = effect of *Ganaspis* sp. wasp. in the **absence** of *Spiroplasma*. Green sets = effect of *Spiroplasma* in the **presence** of *Ganaspis* sp. wasp.

To identify fly genes specifically influenced by the *Spiroplasma*–*Ganaspis* sp. interaction, we adopted the same Venn diagram plus heatmaps strategy as with the *Spiroplasma-L. heterotoma* interaction, except that the *L. heterotoma* treatments were replaced with *Ganaspis* sp. treatments (see Fig. 4). Excluding genes induced by the sole presence of *Spiroplasma* or *Ganaspis* sp., resulted in eight and 21 exclusively up- and down-regulated (respectively) genes at T1 (exclusive green set, Fig. 4A, Dataset S4). The eight upregulated genes were: four snoRNA (*CR33662*, *CR34611*, *CR34616* and *CR34631*), one small nuclear RNA (*CR32162*), lysozyme E, diphthamide methyltransferase (*Dph5*) and mitochondrial ribosomal protein S14 (*mRpS14)*. Among the 21 downregulated genes were *DnaJ-1* (whose product is a cofactor of heat shock proteins), *starvin* (which acts as co-chaperone of Hsp70 proteins), *nervana 3* (which codes for one subunit of a sodium-potassium pump), *nanos* (which encodes a ribosomal RNA-binding protein), and one long non-coding RNA (*CR31400*). A heatmap of the exclusive genes (Fig. S14), did not reveal any interesting expression pattern in comparison with other treatments.

At T1, only one intersection (green+blue) contained genes; all downregulated (*n* = 16; Fig. 4A). A heatmap of expression levels of these 16 genes (Fig. S7) reveals that the two *Spiroplasma* treatments (S^+^Gh and S^+^W^**–**^) have similarly low expression levels, whereas the two treatments lacking *Spiroplasma* (S^**–**^W^**–**^ and S^**–**^Gh) have similarly high expression levels; implying that there is no influence of *Ganaspis* sp. on expression of these genes. Three of the downregulated genes are involved with male functions (*msl-2, roX1* and *roX2*), one gene (*blanks*) is highly expressed in adult testis, and three other genes (*RpL22-like*, *RpS5b* and *RpS19b*) code for ribosomal proteins (Dataset S4).

At T2, 16 and 106 genes were exclusively up- and down-regulated (respectively) in the S^+^Gh treatment (exclusive green set in Fig. 4B, Dataset S5). The 16 exclusively upregulated genes included three mitochondrially-encoded genes (*ND1, CoIII* and *Co*I), *necrotic* (which is negative regulator of the Toll pathway; Green et al., 2000), *dumpy* (which is involved in wing development), and one multidrug resistance gene (*Mdr50*). The 106 exclusively downregulated genes did not group in any GO category, making it difficult to link these genes to informative biological functions. The expression values of several of these genes in the S^+^Gh treatment is similar to S^+^W^**–**^ treatment (Fig. S8). This observation, along with the report that 74 of these genes are highly expressed in adult testis (Dataset S5), suggests that observed expression patterns are likely influenced solely by the presence of *Spiroplasma*. The heatmap of the 16 exclusively upregulated genes (Fig. S9), also indicates a possible influence of only *Spiroplasma*. These genes showed a trend of higher expression levels in the S^+^W^**–**^ treatment relative to the S^**–**^W^**–**^ treatment, but this difference was not significant, possibly due to the high variation among S^+^W^**–**^ replicates.

Only one intersection at T2 (green + blue sets) contained genes (*n* = 1315; all down-regulated; Fig. 4B). These genes are associated with spermatid differentiation/development functions and 1,214 of these 1,315 are predominantly expressed in the adult testis (Dataset S5). Thus, their lower expression in the presence of *Spiroplasma* (with or without *Ganaspis* sp.; see Fig. S10) is likely simply the result of an absence of males in these treatments. An expression pattern akin to the “Lh-affected male gonad genes” was not observed in the case of wasp *Ganaspis* sp.

### Expression of *Spiroplasma* RIP proteins and evidence ribosomal damage

**Figure 5:**
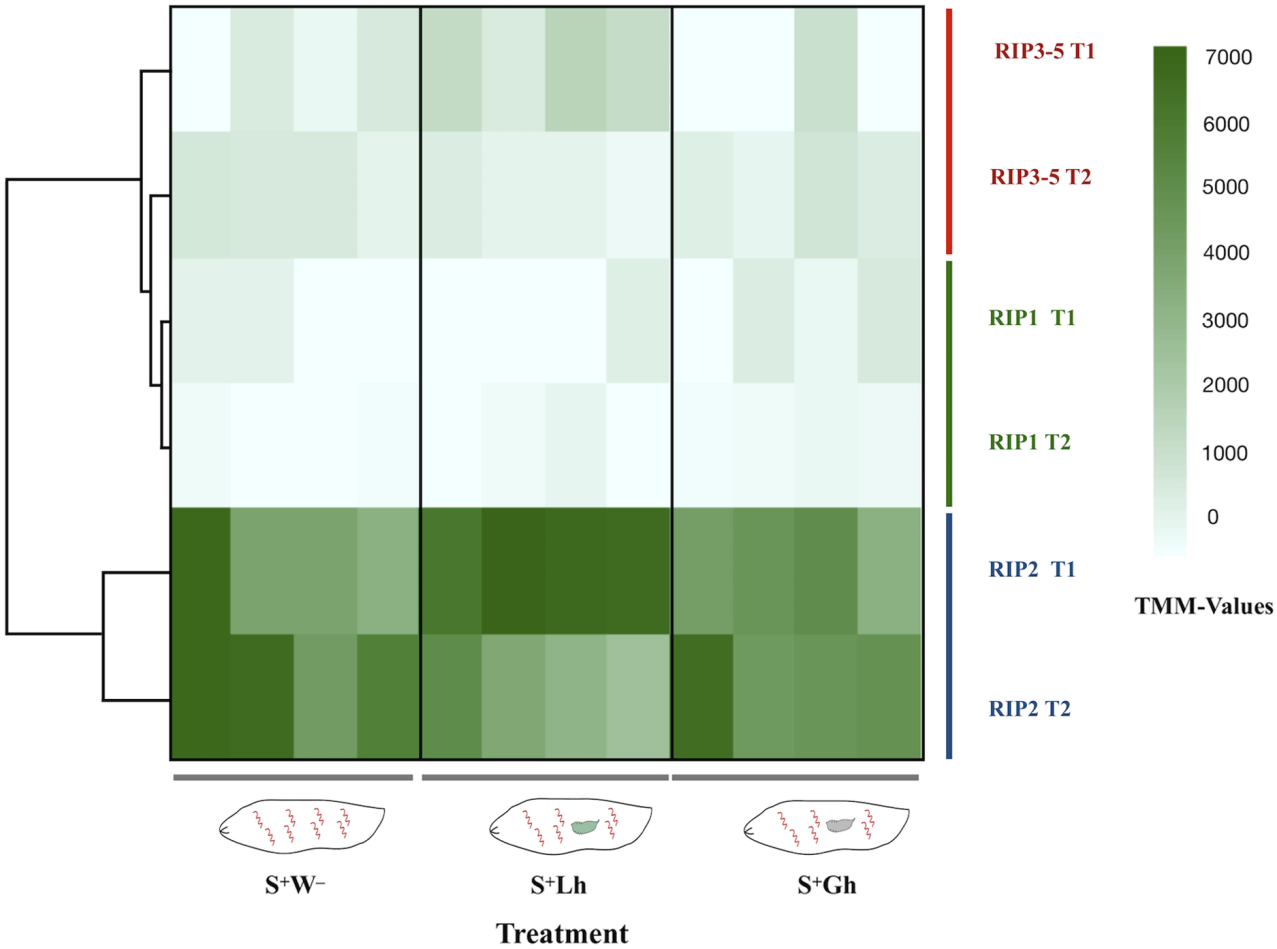
RNA-seq expression levels of the *Spiroplasma* ribosome inactivating protein (RIP) genes. Expression of the five RIP genes in *D. melanogaster* larvae in the absence of wasp parasitism (S^+^W^–^), during parasitism by *L. heterotoma* (S^+^Lh) or *Ganaspis* sp. (S^+^Gh), at 24 h (T1) or 72 h (T2) post-wasp attack (PWA). Values of TMM edgeR normalized counts are shown. Because the RIP3, RIP4 and RIP5 genes are identical at the nucleotide level, their collective expression levels were reported under the label “RIP3-5”.

The total number of reads mapped to *Spiroplasma* excluding ribosomal sequences, was very low (range = 1,080–17,826 per replicate, Table S2). No DE genes were detected, but this could be a reflection of lack of power. For differential gene expression analyses in bacteria, a minimum of four-five million reads per replicate has been recommended (Haas et al., 2012). Due to the relevance of RIP genes in *Spiroplasma* (Ballinger & Perlman, 2017), we examined the read counts of the five RIP genes encoded in the *s*Mel genome, which revealed that the gene encoding RIP2 was the most highly expressed at both time points and in all treatments (Fig. 5).

**Figure 6:**
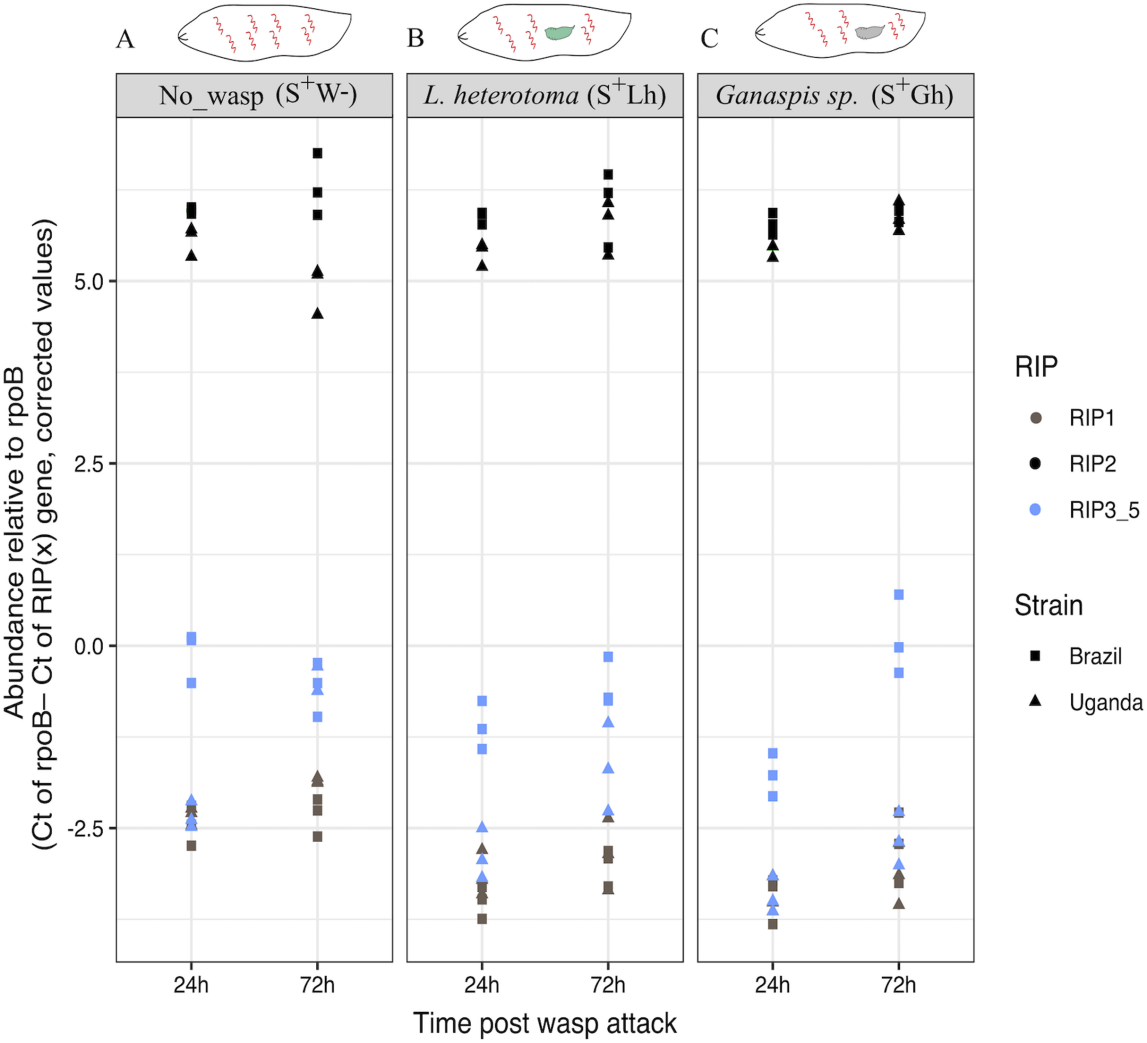
qPCR gene expression of the Ribosome Inactivating Protein (RIP) genes in *D. melanogaster* larvae. (A) Expression of the five RIP genes in the absence of wasp (No_wasp, S^+^W^-^). (B) During parasitism by *L. heterotoma* (S^+^Lh). (C) During parasitism by *Ganaspis sp*. (S^+^Gh). at 24 or 72 h post-wasp attack (PWA). Two different *S. poulsonii* strains were evaluated: sMel-BR and sMel-UG. RIP2 was consistently expressed at a significantly higher level than RIP1 and RIP3-5 in all wasp treatments, time points, and *Spiroplasma* strains (Tukey HSD *P* < 0.05). Because the RIP3, RIP4 and RIP5 genes are identical at the nucleotide level, their collective expression levels were reported under the label “RIP3_5”.

To corroborate the observed patterns of RIP gene expression based on the RNA-seq data, RT-qPCR assays were conducted using both the Uganda (UG) and Brazil (BR) *Spiroplasma s*Mel strains, and the two wasps, *Ganaspis* sp. and *L. heterotoma*. Consistent with the RNA-seq results, the gene encoding RIP2 was the most highly expressed of the RIP genes in both *Spiroplasma* strains regardless of the presence or absence of wasp (Fig. 6; Dataset S2). The full output of the statistical analyses is provided in Supplemental File 1.

A significantly lower percentage of adenines (i.e., evidence of depurination) at the 28S rRNA site targeted by RIPs was only detected in the wasp *L. heterotoma* in the presence of *Spiroplasma* at the time point T2 (X^2^ = 128.58, df = 1, *P* < 2.2E−16; Fig. S11 and Table S3). Evidence consistent with *Spiroplasma*-induced depurination was not detected in ribosomal RNA of the wasp *Ganaspis* sp. or *D. melanogaster* (Figs. S11 and S12; Tables S3 and S4).

## Discussion

To investigate the mechanism by which *Spiroplasma* protects *Drosophila* against wasps, we used an RNA-seq approach to evaluate the transcriptomic response of *D. melanogaster* during interactions involving *Spiroplasma* and two parasitic wasps that differ in their susceptibility to *Spiroplasma*: the *Spiroplasma*-susceptible *L. heterotoma* whose development is obliterated by *S. poulsonii*; and the *Spiroplasma*-resistant *Ganaspis* sp. (G1FL) whose success is unaffected by *S. poulsonii* (Xie et al., 2014; Mateos et al., 2016). The RNAseq approach employed ribodepletion rather than poly-A tail enrichment, with the purpose of recovering both host and *Spiroplasma* mRNA reads. We also used qPCR to evaluate the effect of wasp parasitism on the expression of *Spiroplasma* RIP genes in two closely related substrains of *s*Mel. The RNAseq data were also used to assess the level of ribosomal RNA depurination (i.e., the expected damage induced by RIPs) in the two wasps.

### The adopted ribodepletion procedure is not an ideal strategy for dual transcriptomics in the *Drosophila–Spiroplasma* system

In an attempt to recover mRNA reads from both host and symbiont (also known as “dual transcriptome”), we generated RNA-seq libraries avoiding poly-A-tail enrichment step, typically used for eukaryotic mRNA analyses. Our strategy aimed at removal of ribosomal RNA from both host and symbiont by using the RiboZero Epidemiology kit, which is expected to deplete ribosomal RNA from both bacteria and eukaryotes. A previous application of a similar ribodepletion kit reported removal of >90% of the rRNA sequences of *Drosophila ananassae* (Kumar et al., 2012). In contrast, in our study the reads that mapped to rRNA genes comprised 3.4–78% of the total reads mapped to *D. melanogaster*, indicating a variable and ineffective degree of ribosomal RNA depletion. Furthermore, by evaluating the pattern of reads mapped to the 28S rRNA gene, we found that ribodepletion effectiveness varied by region of the gene (see Fig. S12). We suggest that future applications of ribodepletion in *D. melanogaster* consider including additional probes to capture such regions with higher efficiency. Even if more effective depletion of fly rRNA had been achieved, it is possible that our sequencing effort still would have been inadequate for DE analyses of *Spiroplasma*. Concerning detection of DE genes of *Drosophila*, we acknowledge that according to the power analyses, we are expected to miss DE genes with fold-changes below 3. With the estimated coefficients of variation from our dataset (i.e., BCV range ~0.3–0.4), achieving a power of ~93% for genes with fold-change of 2 would require 5–8 replicates with 100X coverage (or 7–10 replicates with 20X coverage). Notwithstanding these limitations, our results revealed expression patterns consistent with expectations from the known phenotypes induced by *Spiroplasma* and by the wasps.

### Response of *D. melanogaster* to parasitism by *L. heterotoma* or *Ganaspis* sp. in the absence of *Spiroplasma*

The two wasp species used in this study, *L. heterotoma* and *Ganaspis* sp., belong to the same family (Figitidae), but their parasitism strategies are quite different (Schlenke et al., 2007; Mortimer et al., 2013). Our fly transcriptome analysis also revealed differences in the effects of these wasps. We detected evidence of host immune activation by *Ganaspis* sp., but not by *L. heterotoma*. It is possible, however, that *L. heterotoma* induces immune response at levels below those detectable by our experiments. Our results revealed that *L. heterotoma* induces genes related to energy production, which could be a strategy of the wasp to obtain more resources from the host. On the other hand, it may reflect energy investment for a fly function such as immune response, which is energetically expensive (Schlenke et al., 2007; Dolezal et al., 2019). If it indeed reflects investment in immune functions, these have not been detected (this study; Schlenke et al., 2007) and are unsuccessful, as the fly generally does not survive attacks by this strain of *L. heterotoma* (Xie et al., 2014; Jones & Hurst, 2020b).

The *L. heterotoma*-induced downregulation of genes related to spermatogenesis; a phenomenon not induced by *Ganaspis* sp., is an interesting contrast between the two wasps. In accordance with our results, Schlenke et al. (2007) reported downregulation of genes involved with gonad development during parasitism by *L. heterotoma* and *L. boulardi*, albeit at earlier stages. These two independent observations suggest that *L. heterotoma* might induce castration of male hosts. Xie et al. (2011) reported that *Drosophila hydei* males (infected with a non-male-killing strain of *Spiroplasma*) that survived parasitism by *L. heterotoma* (Lh14) had extremely reduced fecundity, but the experiments could not rule out causes other than castration. The phenomenon of male castration has only been reported in parasitoids of the family Braconidae, where the polydnavirus present in the wasp venom induces testis degradation (reviewed in Beckage & Gelman, 2004).

This study is the second one to use a genome-wide approach to evaluate gene expression of *D. melanogaster* in response to *L. heterotoma*. The first one was conducted by Schlenke et al. (2007) and employed microarrays with *D. melanogaster* Oregon-R strain at time points earlier than 24h PWA. In their latest time point (22–24 h PWA), Schlenke et al. (2007) found 37 DE genes (*P* < 0.01, −0.5 > FC > 1.0; fold-changes for genes DE at a *P* < 0.05 were not reported). At a similar time point (our T1), we detected only one DE gene (*thor*), which was not detected by Schlenke et al. (2007). We note that we detected expression of all but one of the 37 DE genes reported by Schlenke et al. (2007). Differences between our results and those of Schlenke et al. (2007) may be attributable to the different methodology (e.g., microarrays vs. RNAseq, different software pipelines and cutoff parameters), or lack of power, particularly to detect DE genes with low FC values and low CPM values (Sheet “Schlenke: FC” in Dataset S4 shows our CPM and FC values for these 37 genes, and FC values reported by Schlenke et al. (2007) for comparison). Other potential differences could be related to *Drosophila* genotypes or *Wolbachia* infection status (i.e., presence/absence), or other experimental conditions or their interactions. The collective evidence from both studies suggests that parasitism by *L. heterotoma* does not induce a detectable immune response in *D. melanogaster*, likely as a result of this wasp’s effective strategies to counter the host’s defenses (Schlenke et al., 2007).

In a different scenario, parasitism by *Ganaspis* sp. induced the Jak/Stat pathway, one of the immune pathways of *D. melanogaster*. Effectors of this pathway (*Tot* and *Tep* genes) were highly upregulated. Tep proteins play an immune role in the mosquito *Anopheles gambiae* against the parasite *Plasmodium berghei* (Blandin et al., 2004). In *D. melanogaster*, deletion of *Tep 1–4* genes impaired resistance against two Gram-positive bacteria and increased the survival of the wasps *L. boulardi* and *Asobara tabida* (Dostálová et al., 2017). The function of *Tot* genes is generally unknown, but they belong to a family of eight genes that are activated by stressful conditions such as bacterial challenge, high temperatures, mechanical pressure, or UV radiation (Ekengren & Hultmark, 2001). Furthermore, one of these genes (*TotM*) enhances immunity against the sexually transmitted fungus *Metarhizium robertsii* (Zhong et al., 2013). Parasitism of *D. melanogaster* by the wasps *L. boulardi* (Schlenke et al., 2007) and *A. tabida* (Salazar-Jaramillo et al., 2017) also induce the up-regulation of *Tep* and *Tot* genes, suggesting that upregulation of these genes could be a common response against wasp parasitism. Another potential signal of immune activation against *Ganaspis* sp. is the induction of genes encoding serine proteases, because these enzymes are involved in triggering immune response in insects (reviewed in Alvarado et al., 2020). The melanization cascade, another feature of the *D. melanogaster* immune response, also appears to be activated by *Ganaspis* sp., as two components of this cascade (*PPO3* and *yellow-f*) were up-regulated. Finally, the upregulation of genes that are known to be expressed preferentially in hemocytes (i.e., *PPO3*, *ItgaPS4 and he*), or involved in hemocyte proliferation (i.e., *pvf2*) implies that hemocytes are activated by *Ganaspis* sp. parasitism. In accordance with this, Mortimer et al. (2013) showed that the number of lamellocytes increases during parasitism by the same *Ganaspis* sp. strain used in the present study. Even though *D. melanogaster* appears to mount an immune response against *Ganaspis* sp., the response is inadequate, as ~100% of flies parasitized by this wasp die (Mortimer et al., 2013; Mateos et al., 2016). The reason likely lies in that the venom of this wasp contains a calcium ATPase that prevents activation of plasmatocytes; a necessary early step of the encapsulation process (Mortimer et al., 2013).

### Response of *D. melanogaster* to parasitism by *L. heterotoma* or *Ganaspis* sp. in the presence of *Spiroplasma*

If the fly contributes to the death of *L. heterotoma* that occurs in the presence of *Spiroplasma*, examination of the transcriptomic response of the fly during the *Spiroplasma–L. heterotoma* interaction might reveal signals of such wasp-inhibiting fly functions. The fly transcriptomic response in the *Spiroplasma–Ganaspis* sp. interaction (where the wasp success in unaffected by *Spiroplasma*), provides a contrast point representing a *Spiroplasma*-resistant wasp. Our analyses revealed several sets of genes whose expression appears to be influenced specifically by the interaction between the *Spiroplasma* and *L. heterotoma* treatments. None of these genes, with exception of attacin-C (*AttC*), were associated with an immune function in *D. melanogaster*. We thus infer that the *Spiroplasma*-mediated wasp-killing mechanism is not strongly influenced by host-encoded immunity, at least at a level detectable by our experiments. Consistent with our findings, an RNA-seq analysis of *Spiroplasma* protection against nematodes in *D. neotestacea* did not detect changes in the host’s immune response (Hamilton et al., 2014). Similarly, Paredes et al. (2016) did not detect an increased cellular response, based on the number of hemocytes, during the *Spiroplasma*–*L. boulardi* interaction. The lack of detectable influence of *Spiroplasma* on host-encoded immunity is consistent with evidence that *Spiroplasma* is not detected as an intruder by the fly, due to the lack of cell wall, where the typical bacterial immune elicitors are found (Hurst et al., 2003; Herren & Lemaitre, 2011).

Regarding the “Lh-affected male gonad genes”, we consider them unlikely candidates for *Spiroplasma*-mediated protection mechanism, because their expression pattern (i.e., S^**–**^W^**–**^ ****> S^**–**^Lh > S^+^W^**–**^ = S^+^Lh) and their reported male-gonad-biased gene expression, seem best explained by: absence of males in the two *Spiroplasma* treatments (i.e., those with the lowest expression levels); and interference of *L. heterotoma* with male gonad development (i.e., with intermediate expression levels).

The remaining genes with apparent influence by the interaction between the *Spiroplasma* and *L. heterotoma* treatments were those assigned to Groups A–C (Fig. 3). These genes could be involved in the *Spiroplasma*-mediated mechanism that leads to the death of *L. heterotoma* or fly survival, or could reflect a side effect of the *Spiroplasma–L. heterotoma* interaction. Group A and Group B (Fig. 3) were comprised of 12 genes whose expression patterns appear to be “restored” in the *Spiroplasma* plus *L. heterotoma* (S^+^Lh) treatment, and do not exhibit such an expression pattern in the context of the *Spiroplasma*-resistant wasp *Ganaspis* sp. The four genes in Group A had relatively low fold changes (<2), and, only two had annotations; *CG42494* is predicted to have chitin-binding activity, whereas *Cyp4d14* encodes a cytochrome P450 domain. Cytochrome P450 are a large family of proteins that is involved in detoxification, but also in developmental processes (Chung et al., 2009). For both of these genes, expression levels are reported to decrease between the L3 and pupa transition. Therefore, if the S^**–**^Lh treatment were developmentally delayed with respect to the S^+^Lh treatment (see discussion below), our observed expression patterns may simply be a consequence of development time differences between the treatments, rather than a cause of wasp death or enhanced fly survival. Furthermore, if the detoxification function of *Cyp4d14* were relevant to the *Spiroplasma*-mediated protection mechanism, we would expect higher expression in the S^+^Lh than in the S^**–**^Lh treatment, which is contrary to our observations.

Genes in Group B exhibit a substantial increase in expression in wild type flies during the larva-to-pupa transition according to flybase (Thurmond et al., 2019). Therefore, our observed expression patterns could simply reflect slight differences in fly development time, where *L. heterotoma* slows down host development, but presence of *Spiroplasma* “restores” development time to that of the unparasitized host. In partial support of this hypothesis, larvae parasitized by *L. hetetoroma* and *L. boulardi* pupate ~2 days later than the unparasitized controls (Schlenke et al., 2007). Whether *Spiroplasma* counteracts the wasp-induced delay in development remains to be determined. Given its possible function as a sulfur-storage protein (Meghlaoui & Veuille, 1997), increased expression of *Fbp2* might reflect a greater availability of nutrients for the fly, which might enhance its tolerance to insults from the wasp.

Genes in Group C had the highest expression level in the S^+^Lh treatment (Fig. 3C), which could suggest a role during *Spiroplasma-L. heterotoma* interaction. The six genes in this group are: one amylase (*Amy-p*); one trypsin inhibitor (*CG42716*); an RNA pseudogene (*CR41609*); a gene referred to as “withdrawn” in Flybase (FBgn0085817); *TotC* and a gene predicted to contain a DNAJ domain (*CG32640*). DNAJ domains are characteristic of co-chaperones, which are proteins that bind to chaperones stimulating ATP hydrolysis (Cheetham & Caplan, 1998). Chaperones in turn are involved in correct protein folding, but also are activated during stressful conditions to prevent cellular damage stress (Voth & Jakob, 2017). Based on their association to “response to stress”, increased expression of *TotC* and the DNAJ-domain-containing gene may contribute to increasing the fly’s tolerance to the wasp parasitism. In addition, *TotA* (FBgn0028396), another stress responsive gene, was upregulated but with an FDR value slightly above our cutoff (0.053; Sheet “All genes: S^**–**^Lh vs. S^+^Lh T2”; Dataset S6).

A caveat to our interpretations is that genes influenced by the interaction of *Spiroplasma* X wasp treatments could also reflect sex differences in response to wasp parasitism, due to the mixed sex (*Spiroplasma*-free) vs. all female (*Spiroplasma*-infected) comparisons. Evidence of sex differences in immunity against other natural enemies has been reported (reviewed by Belmonte et al., 2020). Further studies controlling for sex (e.g., separating males and females in the *Spiroplasma*-free treatment) are needed to address this. Similarly, due to the (unintended) presence of *Wolbachia* in the flies used in the RNAseq analysis, we cannot rule out that *Wolbachia* influences expression of the genes of interest via interactions with the *Spiroplasma* and/or the wasp treatments. It is also possible that the genes of interest are influenced by an interaction between the presence of *Spiroplasma* and the injury effect of wasp oviposition. Nonetheless, the genes deemed as influenced by the *Spiroplasma* × *L. heterotoma* interaction, were not influenced by the interaction of *Spiroplasma* with the other wasp (*Ganaspis* sp.), suggesting that the detected expression patterns are not simply the result of *Spiroplasma* × “injury”. Despite the above limitations, we uncovered a set of candidate genes and phenomena that can be further explored in understanding the *Spiroplasma*-mediated protection mechanism. Experimental manipulation of gene expression of these candidate genes in the absence of *Spiroplasma*, could be used to test whether they influence the outcome of parasitism by *L. heterotoma* and other wasps, including those against which *Spiroplasma s*Mel seems to confer stronger rescue (e.g., *L. boulardi* or *L. victoriae*; Mateos et al., 2016; Paredes et al., 2016; Jones & Hurst, 2020a). Furthermore, detailed analyses on the effects of *Spiroplasma* and wasps on fly development time are warranted.

### RIP expression

Concerning the hypothesis that a *Spiroplasma-*encoded toxin contributes to killing of *Spiroplasma*-susceptible wasps, we found evidence that neither wasp influences expression of RIP genes, but RIP2 gene was highly expressed in contrast to RIP1 or RIP3-5. In a previous report in the absence of parasitism, RIP2 was the most highly expressed of the RIP genes throughout the fly life cycle (only substrain Uganda was examined; Garcia-Arraez et al., 2019). The only context where relatively lower expression of RIP2 and RIP1 in *s*Mel has been reported is in the case of a transcriptome comparison of in vitro culture versus fly hemolymph (Masson et al., 2018), implying that expression of these genes can be regulated. The expression of RIP genes in context of other *Spiroplasma* tolerant/resistant wasps has not been determined.

Our study also reveals that both the *s*Mel-UG and *s*Mel-BR sub-strains of *S. poulsonii* express their RIP genes at similar levels in the presence or absence of *L. heterotoma* and *Ganaspis* sp. Although differences in the genomes of substrains *s*Mel-UG and *s*Mel-BR have been reported (Gerth et al., 2021), our observations suggest that at least for RIP expression patterns the two strains are very similar. Based on expression levels alone, it appears that *s*Mel RIP2 might have a stronger role in wasp death than the other RIP genes. However, it is intriguing that the genome of *s*Hy (the poulsonii-clade native *Spiroplasma* of *D. hydei)* does not harbor a gene with high homology to *s*Mel RIP2. Instead, its genome encodes a gene with high homology to *s*Mel RIP1, as well as putative RIP-encoding genes with homology to genes in *Spiroplasma* strains associated with species other than *D. melanogaster* (Gerth et al., 2021). Strain sHy is known to kill *L. heterotoma* and enhance survival of its host *D. hydei* (Xie, Vilchez & Mateos, 2010). It is thus possible that more than one of the RIPs in the *Spiroplasma* strains that kill *L. heterotoma*, contributes to wasp killing.

Detection of signals of ribosomal depurination in the *Spiroplasma*-susceptible (*L. heterotoma*) but not in the *Spiroplasma*-resistant (*Ganaspis* sp.) wasp is consistent with the hypothesis that RIP-induced depurination contributes to wasp death. Ballinger & Perlman (2017), using a more direct approach to evaluate depurination (i.e., qPCR), reported evidence of *Spiroplasma* (strain sMel-UG) induced depurination in *L. heterotoma*, as well as in *L. boulardi* (another *Spiroplasma*-susceptible wasp), but not in the *Spiroplasma*-resistant pupal ectoparasitic wasp (*Pachycrepoideus vindemmiae*). Ballinger & Perlman (2017) hypothesized that the resistance of *P. vindemmiae* to *Spiroplasma* (and to depurination) may stem from the fact that it is not immersed in *Spiroplasma*-layden hemolymph during development. This explanation, however, would not apply to *Ganaspis* sp., which is a larval endo-parasitoid that spends the initial stages of development in the host hemocoel. Whether RIP-induced depurination is necessary and sufficient to kill susceptible wasps has not been determined. Ideally, the effect of RIP on the wasp would be tested in the absence of *Spiroplasma*, or in “knocked-out” mutants in these genes. In addition, the target cells and the mechanism of entry of *Spiroplasma* RIPs has not been determined. It is unclear whether *Spiroplasma* must colonize wasp tissues in order to deliver RIP or if toxins can be acquired during wasp feeding; both RIP1 and RIP2 proteins have been detected in the fly hemolymph (see Garcia-Arraez et al., 2019). It is also possible that other *Spiroplasma*-encoded putative virulence factors such as chitinase (*ChiD*) or glycerol-3-phosphate oxidase (*glpO*) could contribute to wasp death (Masson et al., 2018). Presence of *glpO* in *S. taiwanense* is proposed to be the cause of its pathogenicity to mosquitoes (Lo & Kuo, 2017).

Assuming RIP is an important factor in wasp killing, the apparent lack of ribosome depurination of *Ganaspis* sp., along with the unaltered expression of RIP genes, suggest that this wasp avoids RIP-induced damage by interfering with translation of RIP mRNA, or by inactivating RIP. Resistance to RIP toxicity has been reported in Lepidoptera, and has been attributed to serine protease-mediated hydrolysis in the digestive tract (Gatehouse et al., 1990). As suggested by Ballinger & Perlman (2017) for *P. vindemmiae*, it is possible that conditions in the gut of *Ganaspis* sp. inactivate ingested RIPs. Alternatively, RIP proteins may be active but unable to enter *Ganaspis* sp. cells, or unable to reach the appropriate cellular compartments to damage ribosomes. It is unlikely that wasp ribosomes that come into contact with RIPs are immune to depurination because of the extremely conserved eukaryotic motif targeted by these proteins.

## Conclusions

In the absence of *Spiroplasma* infection, we found evidence of *Drosophila* immune activation by *Ganaspis* sp., but not by *L. heterotoma*, whose parasitism seems to induce host castration. Acknowledging our expected limited power to detect DE genes with low fold-changes, we identified very few genes whose expression was influenced by the *Spiroplasma–L. heterotoma* interaction, and they do not appear to be related to immune response. Future research is needed to determine whether these candidate genes are involved in the *Spiroplasma*-mediated mechanism that leads to wasp death or fly rescue. We also found that transcript levels of RIP toxin genes were not influenced by wasp parasitism or *Spiroplasma* strain, and that RIP2 was the most highly expressed one. We detected evidence consistent with the action of RIP toxins on the *Spiroplasma*-susceptible wasp (*L. heterotoma*), but not on the *Spiroplasma*-resistant wasp (*Ganaspis* sp.). Therefore, the mechanism by which *Ganaspis* sp. resists/tolerates *Spiroplasma* does not involve inhibition of RIP transcription.

## Supplemental Information

10.7717/peerj.11020/supp-1 Supplemental Information 1 Sequences that mapped to the partial 28S rRNA region of parasitic wasps. Geneious outputs of the sequences that mapped to the 28S rRNA partial region of *L. heterotoma* and *Ganaspis sp* wasps.

10.7717/peerj.11020/supp-2 Supplemental Information 2 Calculations of delta Ct values to the five *Spiroplasma* RIP genes. Delta Ct values for the five *Spiroplasma* encoded RIP genes. The MRSO-BR and MRSO-UG *Spiroplasma* strains were evaluated in the absence of wasps or in the presence of one of two wasps (*L. heterotoma* or *Ganaspis* sp.) at two time points post-wasp attack (24 and 72 h).

10.7717/peerj.11020/supp-3 Supplemental Information 3 Raw count tables of gene expression for *D. melanogaster* and *Spiroplasma*.. Compressed file containing the raw count tables of gene expression for *D. melanogaster* and *Spiroplasma* along different treatments.

10.7717/peerj.11020/supp-4 Supplemental Information 4 Differentially expressed genes and GO erichments at Time point 1 (T1). List of genes differentially expressed (DE) among different treatment pairwise comparisons. GO enriched categories. Fold-change comparisons in different treatments and comparison of our results with a previous publication. All data correspond to time point 1.

10.7717/peerj.11020/supp-5 Supplemental Information 5 Differentially expressed genes and GO enrichments at Time point 2 (T2). List of genes differentially expressed (DE) among different pairwise treatment comparisons. GO enriched categories. All data correspond to time point 2.

10.7717/peerj.11020/supp-6 Supplemental Information 6 Full results of edgeR for the differential expression analysis at time points 1 (T1) and 2 (T2). Complete outputs of edgeR for the differential expression analyses for each of the pairwise treatment comparisons reported in this study.

10.7717/peerj.11020/supp-7 Supplemental Information 7 Recipes to prepare *D. melanogaster* food diet. Instructions to prepare the two food diets (cornmeal and opuntia-banana) used in this study.

10.7717/peerj.11020/supp-8 Supplemental Information 8 Command lines used to run bioinformatic analysis. Examples of the unix command lines used to perform the bioinformatic analysis described in article. The script to identify DE genes by edgeR is also included.

10.7717/peerj.11020/supp-9 Supplemental Information 9 Full output of the statistical analyses of qPCR RIP expression. Full results of the statistical analysis performed with JMP Pro v.15 software. The analyzed data are qPCR values of gene expression of *Spiroplasma* RIP genes.

10.7717/peerj.11020/supp-10 Supplemental Information 10 Power analysis of RNA-seq data. Different combinations of parameters were evaluated. The coverage value represents the range found in our data. The range of Biological coefficient of variation (BCV; estimated by edgeR) of our data was 0.3–0.4.

10.7717/peerj.11020/supp-11 Supplemental Information 11 Mapping statistics of RNA-seq data. Numbers of read sequences mapped to *D. melanogaster* and *Spiroplasma* for each of our generated treatments.

10.7717/peerj.11020/supp-12 Supplemental Information 12 Values of depurination in the 28S rRNA sequences of *L. heterotoma* or *Ganaspis* sp.. Number of adenines or other bases in the specific site of RIP attack in the following treatments: *Spiroplasma*-infected & *L. heterotoma*-exposed (S^+^Lh); *Spiroplasma*-infected & *Ganaspis* sp-exposed (S^+^Gh); *Spiroplasma*-free & *L. heterotoma*-exposed (S^–^Lh); and *Spiroplasma*-free & *Ganaspis* sp.-exposed (S^–^Gh).The two time points post-wasp attack (PWA) were evaluated: T1 = 24 h and T2 = 72 h.

10.7717/peerj.11020/supp-13 Supplemental Information 13 Values of depurination in the 28S rRNA of *D. melanogaster*. Number of adenines or other bases in the specific site of RIP attack. Treatments are; *Spiroplasma*-infected and wasp-free (S^+^W^–^). *Spiroplasma*-free & wasp free (S^–^W^–^). The two time points post-wasp attack (PWA) were evaluated: T1 = 24 h and T2 = 72 h.

10.7717/peerj.11020/supp-14 Supplemental Information 14 Supplemental figures.

## Additional Information and Declarations

### DNA Deposition

The following information was supplied regarding the deposition of DNA sequences: The RNA raw sequences generated in this project are available at NCBI: PRJNA577145, SAMN13020352.

### Data Availability

The following information was supplied regarding data availability: Raw data is available in the Supplemental Files.
