# Supplementary material for: Differential gene expression in a tripartite interaction: Drosophila, Spiroplasma and parasitic wasps
Source: PeerJ. 2021 Mar 4;9:e11020. doi: 10.7717/peerj.11020 (PMC7937342; doi:10.7717/peerj.11020)
Supplement: Supplemental Information 7 — Instructions to prepare the two food diets (cornmeal and opuntia-banana) used in this study. [file peerj-09-11020-s007.pdf]

Standard Cornmeal fly food

Canton S flies were maintained using standard cornmeal food medium. To prepare one liter of this medium:

Ingredients

1L	Deionized water
14.6g	Agar (Genesee Scientific Cat#66-103)
18.4g	Yeast flakes (Genesee Scientific Cat#62-106)
33.0g	Cornmeal (Bought in supermarket)
52.2	Sugar (Bought in supermarket)
2g	Tegosept (Same as Benzoic acid, p-hydroxy-, methyl ester; methyl paraben; nipagen; Lexgard M) (Genesee Scientific Cat#20-258)

1. Put one liter of water in a pot and boil, you can use an electric single burner stove.
2. Add all ingredients into the pot and mix with the whisk often for ~15 min.
3. Turn off the stove
4. Dispense food medium into fly vials

Oregon R flies were maintained using Opuntia Banana food medium. To prepare one liter of this medium:

Ingredients

1L	Deionized water
10g	Agar (Genesee Scientific Cat#66-103)
27.5g	Yeast flakes (Genesee Scientific Cat#62-106)
47.5g	Corn Syrup (Bought in supermarket)
30g	Malt (Genesee Scientific Cat#62-111)
137.5g	Bananas (Bought in supermarket)
2.125g	Opuntia powder (We are currently using “Starwest Botanicals Nopal Cactus Powder Wildcrafted, 1 Pound”)
2g	Tegosept (Same as Benzoic acid, p-hydroxy-, methyl ester; methyl paraben; nipagen; Lexgard M) (Genesee Scientific Cat#20-258)

1. Put one liter of water in a pot and boil, you can use an electric single burner stove.

2. Place bananas, opuntia powder and malt in blender. With ladle take a few spoonfulls of the boiled water from the pot and add to blender. Blend and set aside.

3. Add agar, yeast flakes, corn syrup and Tegosept to the pot with boiling water. Boil and mix with the whisk often for ~10 min.
4. Turn off the stove
5. Dispense food medium into fly vials
