# Supplementary material for: Differential gene expression in a tripartite interaction: Drosophila, Spiroplasma and parasitic wasps
Source: PeerJ. 2021 Mar 4;9:e11020. doi: 10.7717/peerj.11020 (PMC7937342; doi:10.7717/peerj.11020)
Supplement: Supplemental Information 8 — Examples of the unix command lines used to perform the bioinformatic analysis described in article. The script to identify DE genes by edgeR is also included. [file peerj-09-11020-s008.pdf]

Command lines used to run bioinformatics analysis.

```
##Mapping fastq reads to D.mel index with hisat2, index was firstly created

hisat2-build Drosophila_melanogaster.BDGP6.dna.fa index_D_mel

hisat2 --rna-strandness R -p 16 -x index_D_mel -U treatment.fastq -S
mapped_treatment.sam

##Sorting sam file

samtools sort -n -@ 20 mapped_treatment.sam > mapped_treatment_sorted.bam

##Counting reads with featureCounts

featureCounts -T 18 -s 2  -a Drosophila_melanogaster.BDGP6.89.gtf  -t exon -g
gene_id -o treatment_feature_counts.txt treatment_sorted.bam

##Creating index to kallisto, Spiroplasma_transcripts.fasta is a multifasta with
the gene_id and dna sequence of each gene encoded in S. poulsonii genome

kallisto index -i Spiroplasma_index  Spiroplasma_transcripts.fasta

kallisto quant -i Spiroplasma_index --single -l 200 -s 20 -o sample_1.out -t 20 -
-rf-stranded sample_1.fastq
```

Script to obtain DE expressed genes using edgeR

```
# This script was designed  for a pairwaise comparison (Control-Treatment). In
this example each condition (Control-Treatment) has four replicates. Names of the
columns in file (e.g. Count_table_file.txt) have to be named as C.1 C.2 C.3 C.4
T.1 T.2 T.3 and T.4. Genes will be up- or down-regulated in treatment in contrast
to control.


#Reading the count table file
library(edgeR)
library(splines)
Counts <- read.table("ganaspis_spirot2_features.txt", header=T)
head(Counts)
dim(Counts)


##Creating a DGEList object
dgList <- DGEList(counts=Counts, genes=rownames(Counts))
group <- c(1,1,1,1,2,2,2,2)
dgList <- DGEList(counts=Counts, genes=rownames(Counts), group=group)
dgList$samples
head(dgList$counts)
head(dgList$genes)

#Data Exploration
barplot(dgList$samples$lib.size,names=colnames(dgList),las=2)
title("Barplot of library size")
plotMDS(dgList)
title(" un-normalised MDS plot")
logcounts <- cpm(dgList,log=TRUE)
boxplot(logcounts, xlab="", ylab="Log2 counts per million",las=2)
abline(h=median(logcounts),col="blue")
title("Boxplots of log2CPMs (unnormalised)")



#Filtering
countsPerMillion <- cpm(dgList)
summary(countsPerMillion)
countCheck <- countsPerMillion > 1
head(countCheck)
```

```
keep <- which(rowSums(countCheck) >= 4)
dgList <- dgList[keep,]
summary(cpm(dgList))
dim(dgList)
head(countsPerMillion)
write.table(countsPerMillion,file="cpm_values.txt",sep="\t",quote=F)

#Normalisation
dgList <- calcNormFactors(dgList, method="TMM")
tmm_value <- cpm(dgList)
head(tmm_value)
write.table(tmm_value,file="TMM_values.txt",sep="\t",quote=F)

#Data Exploration
plotMDS(dgList)

title("MDS_plot normalized")



#Setting up the Model
sampleType<- rep("C", ncol(dgList))
sampleType[grep("T", colnames(dgList))] <- "T"
designMat <- model.matrix(~sampleType)
designMat

#Estimating Dispersions

dgList = estimateDisp(dgList, design=designMat, robust=TRUE)
plotBCV(dgList)
title("biological coefficient of variation")

dgList$common.dispersion
sqrt(dgList$common.dispersion)

fit <- glmQLFit(dgList, designMat,robust=TRUE )
mod<-glmQLFTest(fit,coef=2)

all_table<-topTags(mod, n=Inf)$table

write.table(all_table,file="all_DE_table.txt",sep="\t",quote=F)

#Plotting
deGenes <- decideTestsDGE(mod, p=0.05)
deGenes <- rownames(mod)[as.logical(deGenes)]
plotMD(mod, de.tags=deGenes)
abline(h=c(-1, 1), col=2)
```

```
topTags(mod,n=100)$table

plotMD(mod, main="DE genes")
abline(h=c(-1, 1), col=2)

#Getting DE genes
tabla = read.table("all_DE_table.txt", header=T)
dim(tabla)
head(tabla)
upper=subset(all_table,logFC >= 0.585 & FDR <= 0.05)
dim(upper)

write.table(upper,file="up_DE_genes.txt",sep="\t",quote=F)

down=subset(tabla,logFC <= -0.585 & FDR < 0.05)

dim(down)

write.table(down,file="down_DE_genes.txt",sep="\t",quote=F)
```

Example to generate the plot corresponding to Figure 6

```
library(ggplot2)
rip_values = read.table(file.choose(), header=T)

rip_values$Treatment_f<- factor(rip_values$Treatment, levels
=c("S+W0","S+Lh","S+Gh"))

p<-ggplot(rip_values, aes(x=Time, y=Value, color=RIP, shape=Strain)) +
  geom_point(aes(shape=Strain)) + scale_shape_manual(values=c(15,17)) +
  theme_bw() + scale_y_continuous(name="Abundance relative to rpoB\n ( Ct of rpoB
– Ct of RIP(x) gene, corrected values)") +  scale_x_discrete(name= "Time post
wasp attack")


p + facet_grid(~Treatment_f)

treat_lab<-c("No_wasp","Lh","Gh")
names(treat_lab)<-c("S+W0","S+Lh","S+Gh")

p + facet_grid(~Treatment_f, labeller = labeller(Treatment_f=treat_lab))
```

Example to generate the plot corresponding to Figure S1. Graph of RNA-seq power results

```
library(ggplot2)
power<-read.table(file.choose(), header = T)
cv_labs<-c("CV=0.3", "CV=0.4")
names(cv_labs)<-c(0.3,0.4)

ggplot(power, aes(x=FC, y=Power)) + geom_point(aes(shape=Replicates,
color=Coverage)) + facet_grid(CV ~., labeller = labeller(CV=cv_labs))  +
theme_bw() + scale_x_continuous(c(1.5,2,3,4), name = "Fold Change")
```

Example to generate the plot corresponding to Figure S10. Signals of wasp ribosome depurination in the presence of *Spiroplasma* in *L. heterotoma* (S+Lh) or *Ganaspis sp*. (S+Gh) ribosomal reads.

```
## File to generate this plot is Dataset S7. Values of depurination in the 28S
rRNA of L. heterotoma or Ganaspis sp.

library(ggpubr)
library(ggplot2)

depur = read.table("Input_file.txt", header=T, row.names = NULL)

dpr1<-subset(depur, Time=="24_PWA")

dpr2<-subset(depur, Time=="72_PWA")
```

```
box1<-ggplot(dpr1, aes(x=Treatment, y=Total_sequences, fill=Treatment)) +
geom_boxplot(outlier.shape = NA)  + scale_y_continuous( name="Numbers of reads")
+ theme_bw() + geom_point(pch = 21, position = position_jitterdodge())

box1<- box1+ theme(legend.position = "none")


box2<-ggplot(dpr2, aes(x=Treatment, y=Total_sequences, fill=Treatment)) +
geom_boxplot(outlier.shape = NA)  + theme(axis.title.y =    element_blank(),
legend.position = "none") + theme_bw() +  theme(legend.position = "none") +
theme(axis.title.y = element_blank()) + geom_point(pch = 21, position =
position_jitterdodge())

per1<-ggplot(dpr1, aes(x=Treatment, y=Percentage, fill=Treatment)) +
geom_boxplot(outlier.shape = NA)  + theme(legend.position = "none") +
scale_y_continuous(name = "% of Adenines in target site") + theme_bw() +
theme(legend.position = "none") + theme(axis.title.x =
element_blank())+geom_point(pch = 21, position = position_jitterdodge())

per2<-ggplot(dpr2, aes(x=Treatment, y=Percentage, fill=Treatment)) +
geom_boxplot(outlier.shape = NA) + theme(legend.position = "none") +
scale_y_continuous(name = "% of Adenines in target site") + theme_bw() +
theme(legend.position = "none") + theme(axis.title.x = element_blank(),
axis.title.y = element_blank()) + geom_point(pch = 21, position =
position_jitterdodge())


 ggarrange(per1, per2,box1,box2,labels = c("a)","c)","b)","d)"), ncol = 2, nrow =
2, widths = 0.1, heights = 0.3, hjust = -2, vjust = 0.9, font.label =
list(size=10) )
```

Heatmaps

```
##Exmaple to generate one heatmap using pheatmap package.  In some cases
different pattern of colours were selected.

pheatmap(valores, scale = "row", cluster_rows = T, cluster_cols = F, border_color
= F, cellwidth = 15, cellheight = 20)
```

Statistical analysis of depurination in wasp ribosomal sequences.

```
library(dplyr)
library(arm)
depur <- read.table(file.choose(), header=T)
head(depur)
heterotoma_t1<- filter(depur, Wasp=="L.heterotoma", Time=="24_PWA")
fit_t1_bayes <- cbind(heterotoma_t1$Adenine,heterotoma_t1$Other)
fit_t1_bayes

fit_t1_bayesb<-bayesglm(fit_t1_bayes~Symbiont, family=binomial, data =
heterotoma_t1)
summary(fit_t1_bayesb) #10.9/6, we will use quasibinomial
fit_t1_bayesq<-bayesglm(fit_t1_bayes~Symbiont, family=quasibinomial, data =
heterotoma_t1)
summary(fit_t1_bayesq)
car::Anova(fit_t1_bayesq)

heterotoma_t2<-filter(depur, Wasp=="L.heterotoma", Time=="72_PWA")
fit_t2_bayes <- cbind(heterotoma_t2$Adenine,heterotoma_t2$Other)
fit_t2_bayes

fit_t2_bayesb<-bayesglm(fit_t2_bayes~Symbiont, family=binomial, data =
heterotoma_t2)
summary(fit_t2_bayesb) # 2.754/6 underdispertion

fit_t2_bayesq<-bayesglm(fit_t2_bayes~Symbiont, family=quasibinomial, data =
heterotoma_t2)
summary(fit_t2_bayesq)
car::Anova(fit_t2_bayesq)
```
