# Supplementary material for: Differential gene expression in a tripartite interaction: Drosophila, Spiroplasma and parasitic wasps
Source: PeerJ. 2021 Mar 4;9:e11020. doi: 10.7717/peerj.11020 (PMC7937342; doi:10.7717/peerj.11020)

Generalized Regression for Value

Model Comparison

Show	Response Distribution	Estimation Method	Validation Method	Nonzero Parameters	AICc	BIC	R-Square
☑	Normal	Standard Least Squares	None	37	130.44475	189.51217	0.9953923

Model Launch

Response Distribution

Normal

Estimation Method

Lasso

☐ Adaptive

Validation Method

AICc

☑ Early Stopping

Go

Standard Least Squares

Model Summary

Response	Value
Distribution	Normal
Estimation Method	Standard Least Squares
Validation Method	None
Mean Model Link	Identity
Scale Model Link	Identity

Measure	
Number of rows	108
Sum of Frequencies	108
-LogLikelihood	8.1366596
Number of Parameters	37
BIC	189.51217
AICc	130.44475
RSquare	0.9953923

Generalized Regression for Value

Standard Least Squares

Model Summary

Measure	
RSquare Adj	0.9931524
RASE	0.260905

Parameter Estimates for Original Predictors

Term	Estimate	Std Error	Wald ChiSquare	Prob > ChiSquare	Lower 95%	Upper 95%
Intercept	-0.506486	0.1844877	7.5370306	0.0060*	-0.868075	-0.144897
Treatment[Gh-no_wasp]*Strain[Brazil-Uganda]*RIP[RIP1-RIP3_5]*Time[24h-72h]	2.6652302	0.7379507	13.044121	0.0003*	1.2188735	4.1115869
Treatment[Gh-no_wasp]*Strain[Brazil-Uganda]*RIP[RIP2-RIP3_5]*Time[24h-72h]	4.68575	0.7379507	40.318427	<.0001*	3.2393933	6.1321067
Treatment[Lh-no_wasp]*Strain[Brazil-Uganda]*RIP[RIP1-RIP3_5]*Time[24h-72h]	1.0994482	0.7379507	2.219703	0.1363	-0.346909	2.5458049
Treatment[Lh-no_wasp]*Strain[Brazil-Uganda]*RIP[RIP2-RIP3_5]*Time[24h-72h]	2.8613131	0.7379507	15.034055	0.0001*	1.4149564	4.3076698
Strain[Brazil-Uganda]*RIP[RIP1-RIP3_5]*Time[24h-72h]	-1.951067	0.5218099	13.980409	0.0002*	-2.973796	-0.928339
Strain[Brazil-Uganda]*RIP[RIP2-RIP3_5]*Time[24h-72h]	-3.270031	0.5218099	39.271655	<.0001*	-4.29276	-2.247303
Treatment[Gh-no_wasp]*Strain[Brazil-Uganda]*RIP[RIP1-RIP3_5]	-1.971147	0.5218099	14.269645	0.0002*	-2.993875	-0.948418
Treatment[Gh-no_wasp]*Strain[Brazil-Uganda]*RIP[RIP2-RIP3_5]	-4.160043	0.5218099	63.558113	<.0001*	-5.182772	-3.137314
Treatment[Lh-no_wasp]*Strain[Brazil-Uganda]*RIP[RIP1-RIP3_5]	-0.878931	0.5218099	2.8371693	0.0921	-1.90166	0.1437973
Treatment[Lh-no_wasp]*Strain[Brazil-Uganda]*RIP[RIP2-RIP3_5]	-2.308038	0.5218099	19.564171	<.0001*	-3.330767	-1.28531
Treatment[Gh-no_wasp]*Strain[Brazil-Uganda]*Time[24h-72h]	-3.397517	0.5218099	42.393432	<.0001*	-4.420245	-2.374788
Treatment[Lh-no_wasp]*Strain[Brazil-Uganda]*Time[24h-72h]	-1.664815	0.5218099	10.179057	0.0014*	-2.687544	-0.642087
Treatment[Gh-no_wasp]*RIP[RIP1-RIP3_5]*Time[24h-72h]	-0.877172	0.5218099	2.8258224	0.0928	-1.899901	0.1455566
Treatment[Gh-no_wasp]*RIP[RIP2-RIP3_5]*Time[24h-72h]	-2.154512	0.5218099	17.047994	<.0001*	-3.177241	-1.131784
Treatment[Lh-no_wasp]*RIP[RIP1-RIP3_5]*Time[24h-72h]	-0.427684	0.5218099	0.6717719	0.4124	-1.450413	0.5950444
Treatment[Lh-no_wasp]*RIP[RIP2-RIP3_5]*Time[24h-72h]	-1.669579	0.5218099	10.237393	0.0014*	-2.692308	-0.646851
Treatment[Gh-no_wasp]	-2.153584	0.260905	68.133231	<.0001*	-2.664948	-1.64222
Treatment[Lh-no_wasp]	-1.169513	0.260905	20.093041	<.0001*	-1.680878	-0.658149
Strain[Brazil-Uganda]	-0.067589	0.260905	0.067111	0.7956	-0.578954	0.4437748
RIP[RIP1-RIP3_5]	-1.342449	0.260905	26.474712	<.0001*	-1.853814	-0.831085
RIP[RIP2-RIP3_5]	5.4237771	0.260905	432.15447	<.0001*	4.9124128	5.9351414
Time[24h-72h]	-1.827899	0.260905	49.083986	<.0001*	-2.339264	-1.316535
Treatment[Gh-no_wasp]*Strain[Brazil-Uganda]	2.8307118	0.3689753	58.856802	<.0001*	2.1075335	3.5538902
Treatment[Lh-no_wasp]*Strain[Brazil-Uganda]	1.2056423	0.3689753	10.676829	0.0011*	0.4824639	1.9288206
Treatment[Gh-no_wasp]*RIP[RIP1-RIP3_5]	0.86877	0.3689753	5.5438954	0.0185*	0.1455917	1.5919484
Treatment[Gh-no_wasp]*RIP[RIP2-RIP3_5]	3.1092383	0.3689753	71.008996	<.0001*	2.38606	3.8324167
Treatment[Lh-no_wasp]*RIP[RIP1-RIP3_5]	0.160114	0.3689753	0.1883058	0.6643	-0.563064	0.8832923

Generalized Regression for Value

Standard Least Squares

Parameter Estimates for Original Predictors

Term	Estimate	Std Error	Wald ChiSquare	Prob > ChiSquare	Lower 95%	Upper 95%
Treatment[Lh-no_wasp]*RIP[RIP2-RIP3_5]	2.0243669	0.3689753	30.101201	<.0001*	1.3011886	2.7475453
Treatment[Gh-no_wasp]*Time[24h-72h]	1.053851	0.3689753	8.1576259	0.0043*	0.3306727	1.7770294
Treatment[Lh-no_wasp]*Time[24h-72h]	0.629072	0.3689753	2.9067394	0.0882	-0.094106	1.3522504
Strain[Brazil-Uganda]*RIP[RIP1-RIP3_5]	-0.410005	0.3689753	1.2347637	0.2665	-1.133183	0.3131733
Strain[Brazil-Uganda]*RIP[RIP2-RIP3_5]	1.441441	0.3689753	15.261566	<.0001*	0.7182627	2.1646194
Strain[Brazil-Uganda]*Time[24h-72h]	2.2972192	0.3689753	38.762361	<.0001*	1.5740408	3.0203975
RIP[RIP1-RIP3_5]*Time[24h-72h]	1.3459961	0.3689753	13.307391	0.0003*	0.6228177	2.0691744
RIP[RIP2-RIP3_5]*Time[24h-72h]	2.4779155	0.3689753	45.100184	<.0001*	1.7547371	3.2010938

Normal Distribution Parameters	Estimate	Std Error	Wald ChiSquare	Prob > ChiSquare	Lower 95%	Upper 95%
Scale	0.319542	0.0307479	108	<.0001*	0.2592771	0.3798069

Effect Tests

Source	Nparm	DF	Sum of Squares	F Ratio	Prob > F
RIP	2	2	77.00135	377.06171	<.0001*
Treatment*RIP	4	4	8.2872123	20.290491	<.0001*
Treatment	2	2	6.9740807	34.150814	<.0001*
Treatment*Strain	2	2	6.0536768	29.643762	<.0001*
Time	1	1	5.0118232	49.083986	<.0001*
Treatment*Strain*RIP	4	4	6.5541147	16.047158	<.0001*
RIP*Time	2	2	4.616506	22.606196	<.0001*
Strain*Time	1	1	3.957912	38.762361	<.0001*
Treatment*Strain*Time	2	2	4.3292462	21.199537	<.0001*
Strain*RIP*Time	2	2	4.0598589	19.880396	<.0001*
Treatment*Strain*RIP*Time	4	4	4.2907848	10.505599	<.0001*
Strain*RIP	2	2	2.8368545	13.891564	<.0001*
Treatment*RIP*Time	4	4	1.9990198	4.8944195	0.0015*
Treatment*Time	2	2	0.8433854	4.1299059	0.0200*
Strain	1	1	0.0068525	0.067111	0.7963

Generalized Regression for Value

Standard Least Squares

Diagnostic Bundle

Residual by Predicted Plot

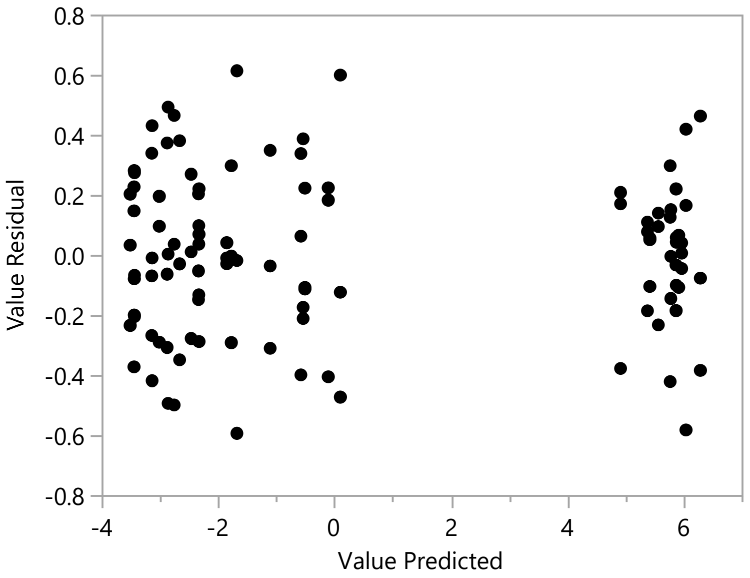

Histogram of Residuals

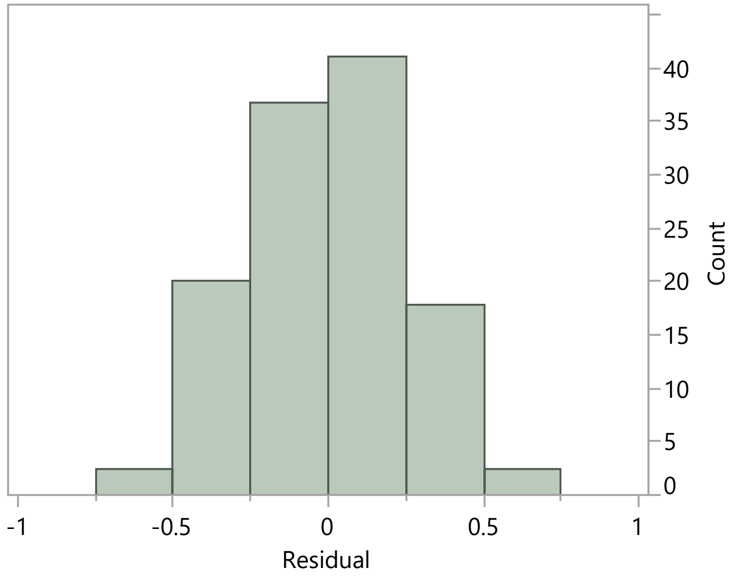

Generalized Regression for Value

Standard Least Squares

Diagnostic Bundle

Residual by Row Plot

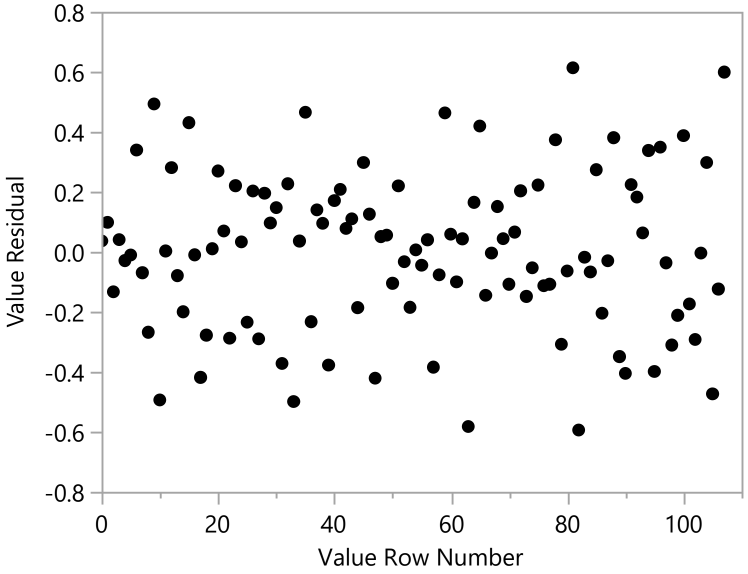

Fitted Probability of Observing a Larger Response

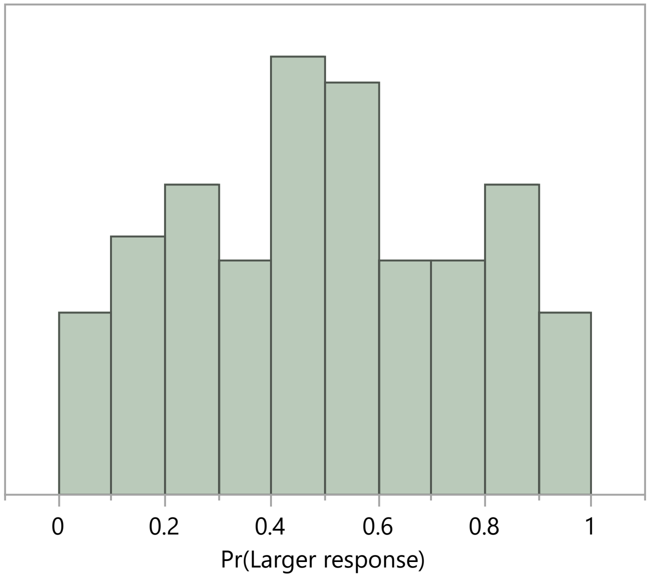

Generalized Regression for Value

Standard Least Squares

Normal Quantile Plot

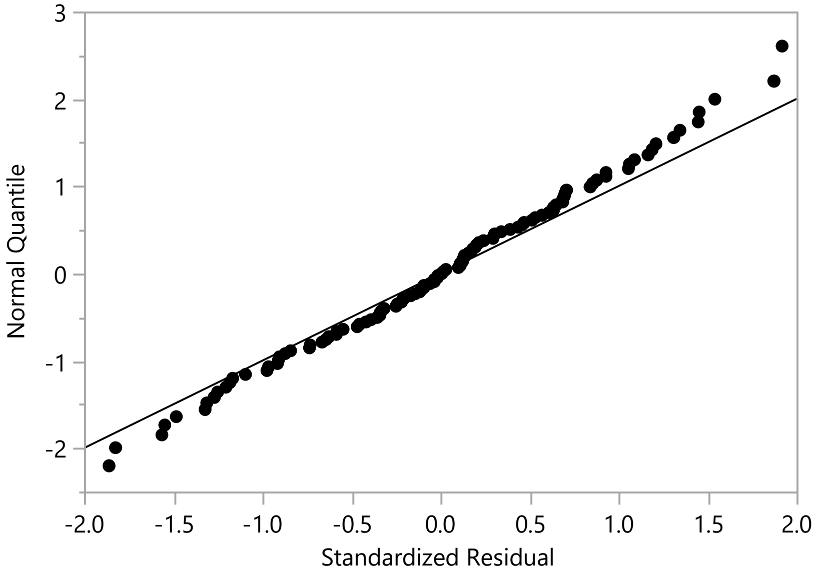

Generalized Regression for Value

Standard Least Squares

Multiple Comparisons for Treatment*Strain*RIP*Time

Least Squares Means Estimates

Treatment	Strain	RIP	Time	Estimate	Std Error	DF	Lower 95%	Upper 95%
Gh	Brazil	RIP1	24h	-3.443138	0.18448766	72	-3.810907	-3.075368
Gh	Brazil	RIP1	72h	-2.751779	0.18448766	72	-3.119548	-2.384009
Gh	Brazil	RIP2	24h	5.782242	0.18448766	72	5.414472	6.150011
Gh	Brazil	RIP2	72h	5.917466	0.18448766	72	5.549696	6.285235
Gh	Brazil	RIP3_5	24h	-1.771293	0.18448766	72	-2.139063	-1.403524
Gh	Brazil	RIP3_5	72h	0.103052	0.18448766	72	-0.264717	0.470822
Gh	Uganda	RIP1	24h	-3.438974	0.18448766	72	-3.806743	-3.071204
Gh	Uganda	RIP1	72h	-3.133749	0.18448766	72	-3.501519	-2.765980
Gh	Uganda	RIP2	24h	5.422300	0.18448766	72	5.054531	5.790070
Gh	Uganda	RIP2	72h	5.872945	0.18448766	72	5.505176	6.240715
Gh	Uganda	RIP3_5	24h	-3.434118	0.18448766	72	-3.801888	-3.066349
Gh	Uganda	RIP3_5	72h	-2.660070	0.18448766	72	-3.027839	-2.292301
Lh	Brazil	RIP1	24h	-3.508949	0.18448766	72	-3.876719	-3.141180
Lh	Brazil	RIP1	72h	-3.009218	0.18448766	72	-3.376988	-2.641449
Lh	Brazil	RIP2	24h	5.876795	0.18448766	72	5.509025	6.244564
Lh	Brazil	RIP2	72h	6.043600	0.18448766	72	5.675831	6.411369
Lh	Brazil	RIP3_5	24h	-1.104370	0.18448766	72	-1.472139	-0.736601
Lh	Brazil	RIP3_5	72h	-0.537947	0.18448766	72	-0.905716	-0.170177
Lh	Uganda	RIP1	24h	-3.138850	0.18448766	72	-3.506620	-2.771081
Lh	Uganda	RIP1	72h	-2.858335	0.18448766	72	-3.226104	-2.490565
Lh	Uganda	RIP2	24h	5.381654	0.18448766	72	5.013884	5.749423
Lh	Uganda	RIP2	72h	5.772145	0.18448766	72	5.404375	6.139914
Lh	Uganda	RIP3_5	24h	-2.874826	0.18448766	72	-3.242596	-2.507057
Lh	Uganda	RIP3_5	72h	-1.675999	0.18448766	72	-2.043769	-1.308230
no_wasp	Brazil	RIP1	24h	-2.462281	0.18448766	72	-2.830051	-2.094512
no_wasp	Brazil	RIP1	72h	-2.326530	0.18448766	72	-2.694299	-1.958761
no_wasp	Brazil	RIP2	24h	5.968347	0.18448766	72	5.600577	6.336116
no_wasp	Brazil	RIP2	72h	6.291143	0.18448766	72	5.923373	6.658912
no_wasp	Brazil	RIP3_5	24h	-0.104756	0.18448766	72	-0.472525	0.263014
no_wasp	Brazil	RIP3_5	72h	-0.574076	0.18448766	72	-0.941845	-0.206306
no_wasp	Uganda	RIP1	24h	-2.330839	0.18448766	72	-2.698608	-1.963069
no_wasp	Uganda	RIP1	72h	-1.848935	0.18448766	72	-2.216705	-1.481166

Generalized Regression for Value

Standard Least Squares

Multiple Comparisons for Treatment*Strain*RIP*Time

Least Squares Means Estimates

Treatment	Strain	RIP	Time	Estimate	Std Error	DF	Lower 95%	Upper 95%
no_wasp	Uganda	RIP2	24h	5.567307	0.18448766	72	5.199538	5.935077
no_wasp	Uganda	RIP2	72h	4.917291	0.18448766	72	4.549522	5.285060
no_wasp	Uganda	RIP3_5	24h	-2.334385	0.18448766	72	-2.702155	-1.966616
no_wasp	Uganda	RIP3_5	72h	-0.506486	0.18448766	72	-0.874255	-0.138717

Tukey HSD All Pairwise Comparisons

Quantile = 4.00311 , Adjusted DF = 72.0 , Adjustment = Tukey

All Pairwise Differences

Treatment	Strain	RIP	Time	-Treatment	-Strain	-RIP	-Time	Difference	Std Error	t Ratio	Prob>|t|	Lower 95%	Upper 95%
Gh	Brazil	RIP1	24h	Gh	Brazil	RIP1	72h	-0.69136	0.2609050	-2.65	0.7424	-1.7358	0.3531
Gh	Brazil	RIP1	24h	Gh	Brazil	RIP2	24h	-9.22538	0.2609050	-35.36	<.0001*	-10.2698	-8.1809
Gh	Brazil	RIP1	24h	Gh	Brazil	RIP2	72h	-9.36060	0.2609050	-35.88	<.0001*	-10.4050	-8.3162
Gh	Brazil	RIP1	24h	Gh	Brazil	RIP3_5	24h	-1.67184	0.2609050	-6.41	<.0001*	-2.7163	-0.6274
Gh	Brazil	RIP1	24h	Gh	Brazil	RIP3_5	72h	-3.54619	0.2609050	-13.59	<.0001*	-4.5906	-2.5018
Gh	Brazil	RIP1	24h	Gh	Uganda	RIP1	24h	-0.00416	0.2609050	-0.02	1.0000	-1.0486	1.0403
Gh	Brazil	RIP1	24h	Gh	Uganda	RIP1	72h	-0.30939	0.2609050	-1.19	1.0000	-1.3538	0.7350
Gh	Brazil	RIP1	24h	Gh	Uganda	RIP2	24h	-8.86544	0.2609050	-33.98	<.0001*	-9.9099	-7.8210
Gh	Brazil	RIP1	24h	Gh	Uganda	RIP2	72h	-9.31608	0.2609050	-35.71	<.0001*	-10.3605	-8.2717
Gh	Brazil	RIP1	24h	Gh	Uganda	RIP3_5	24h	-0.00902	0.2609050	-0.03	1.0000	-1.0535	1.0354
Gh	Brazil	RIP1	24h	Gh	Uganda	RIP3_5	72h	-0.78307	0.2609050	-3.00	0.4842	-1.8275	0.2614
Gh	Brazil	RIP1	24h	Lh	Brazil	RIP1	24h	0.06581	0.2609050	0.25	1.0000	-0.9786	1.1102
Gh	Brazil	RIP1	24h	Lh	Brazil	RIP1	72h	-0.43392	0.2609050	-1.66	0.9993	-1.4784	0.6105
Gh	Brazil	RIP1	24h	Lh	Brazil	RIP2	24h	-9.31993	0.2609050	-35.72	<.0001*	-10.3644	-8.2755
Gh	Brazil	RIP1	24h	Lh	Brazil	RIP2	72h	-9.48674	0.2609050	-36.36	<.0001*	-10.5312	-8.4423
Gh	Brazil	RIP1	24h	Lh	Brazil	RIP3_5	24h	-2.33877	0.2609050	-8.96	<.0001*	-3.3832	-1.2943
Gh	Brazil	RIP1	24h	Lh	Brazil	RIP3_5	72h	-2.90519	0.2609050	-11.14	<.0001*	-3.9496	-1.8608
Gh	Brazil	RIP1	24h	Lh	Uganda	RIP1	24h	-0.30429	0.2609050	-1.17	1.0000	-1.3487	0.7401
Gh	Brazil	RIP1	24h	Lh	Uganda	RIP1	72h	-0.58480	0.2609050	-2.24	0.9420	-1.6292	0.4596
Gh	Brazil	RIP1	24h	Lh	Uganda	RIP2	24h	-8.82479	0.2609050	-33.82	<.0001*	-9.8692	-7.7804
Gh	Brazil	RIP1	24h	Lh	Uganda	RIP2	72h	-9.21528	0.2609050	-35.32	<.0001*	-10.2597	-8.1709
Gh	Brazil	RIP1	24h	Lh	Uganda	RIP3_5	24h	-0.56831	0.2609050	-2.18	0.9580	-1.6127	0.4761
Gh	Brazil	RIP1	24h	Lh	Uganda	RIP3_5	72h	-1.76714	0.2609050	-6.77	<.0001*	-2.8116	-0.7227

Generalized Regression for Value

Standard Least Squares

Multiple Comparisons for Treatment*Strain*RIP*Time

Tukey HSD All Pairwise Comparisons

All Pairwise Differences

Treatment	Strain	RIP	Time	-Treatment	-Strain	-RIP	-Time	Difference	Std Error	t Ratio	Prob>|t|	Lower 95%	Upper 95%
Gh	Brazil	RIP1	24h	no_wasp	Brazil	RIP1	24h	-0.98086	0.2609050	-3.76	0.0979	-2.0253	0.0636
Gh	Brazil	RIP1	24h	no_wasp	Brazil	RIP1	72h	-1.11661	0.2609050	-4.28	0.0217*	-2.1610	-0.0722
Gh	Brazil	RIP1	24h	no_wasp	Brazil	RIP2	24h	-9.41148	0.2609050	-36.07	<.0001*	-10.4559	-8.3671
Gh	Brazil	RIP1	24h	no_wasp	Brazil	RIP2	72h	-9.73428	0.2609050	-37.31	<.0001*	-10.7787	-8.6898
Gh	Brazil	RIP1	24h	no_wasp	Brazil	RIP3_5	24h	-3.33838	0.2609050	-12.80	<.0001*	-4.3828	-2.2940
Gh	Brazil	RIP1	24h	no_wasp	Brazil	RIP3_5	72h	-2.86906	0.2609050	-11.00	<.0001*	-3.9135	-1.8246
Gh	Brazil	RIP1	24h	no_wasp	Uganda	RIP1	24h	-1.11230	0.2609050	-4.26	0.0229*	-2.1567	-0.0679
Gh	Brazil	RIP1	24h	no_wasp	Uganda	RIP1	72h	-1.59420	0.2609050	-6.11	<.0001*	-2.6386	-0.5498
Gh	Brazil	RIP1	24h	no_wasp	Uganda	RIP2	24h	-9.01044	0.2609050	-34.54	<.0001*	-10.0549	-7.9660
Gh	Brazil	RIP1	24h	no_wasp	Uganda	RIP2	72h	-8.36043	0.2609050	-32.04	<.0001*	-9.4049	-7.3160
Gh	Brazil	RIP1	24h	no_wasp	Uganda	RIP3_5	24h	-1.10875	0.2609050	-4.25	0.0239*	-2.1532	-0.0643
Gh	Brazil	RIP1	24h	no_wasp	Uganda	RIP3_5	72h	-2.93665	0.2609050	-11.26	<.0001*	-3.9811	-1.8922
Gh	Brazil	RIP1	72h	Gh	Brazil	RIP2	24h	-8.53402	0.2609050	-32.71	<.0001*	-9.5785	-7.4896
Gh	Brazil	RIP1	72h	Gh	Brazil	RIP2	72h	-8.66924	0.2609050	-33.23	<.0001*	-9.7137	-7.6248
Gh	Brazil	RIP1	72h	Gh	Brazil	RIP3_5	24h	-0.98049	0.2609050	-3.76	0.0982	-2.0249	0.0639
Gh	Brazil	RIP1	72h	Gh	Brazil	RIP3_5	72h	-2.85483	0.2609050	-10.94	<.0001*	-3.8993	-1.8104
Gh	Brazil	RIP1	72h	Gh	Uganda	RIP1	24h	0.68719	0.2609050	2.63	0.7531	-0.3572	1.7316
Gh	Brazil	RIP1	72h	Gh	Uganda	RIP1	72h	0.38197	0.2609050	1.46	0.9999	-0.6625	1.4264
Gh	Brazil	RIP1	72h	Gh	Uganda	RIP2	24h	-8.17408	0.2609050	-31.33	<.0001*	-9.2185	-7.1296
Gh	Brazil	RIP1	72h	Gh	Uganda	RIP2	72h	-8.62472	0.2609050	-33.06	<.0001*	-9.6692	-7.5803
Gh	Brazil	RIP1	72h	Gh	Uganda	RIP3_5	24h	0.68234	0.2609050	2.62	0.7653	-0.3621	1.7268
Gh	Brazil	RIP1	72h	Gh	Uganda	RIP3_5	72h	-0.09171	0.2609050	-0.35	1.0000	-1.1361	0.9527
Gh	Brazil	RIP1	72h	Lh	Brazil	RIP1	24h	0.75717	0.2609050	2.90	0.5585	-0.2873	1.8016
Gh	Brazil	RIP1	72h	Lh	Brazil	RIP1	72h	0.25744	0.2609050	0.99	1.0000	-0.7870	1.3019
Gh	Brazil	RIP1	72h	Lh	Brazil	RIP2	24h	-8.62857	0.2609050	-33.07	<.0001*	-9.6730	-7.5841
Gh	Brazil	RIP1	72h	Lh	Brazil	RIP2	72h	-8.79538	0.2609050	-33.71	<.0001*	-9.8398	-7.7509
Gh	Brazil	RIP1	72h	Lh	Brazil	RIP3_5	24h	-1.64741	0.2609050	-6.31	<.0001*	-2.6918	-0.6030
Gh	Brazil	RIP1	72h	Lh	Brazil	RIP3_5	72h	-2.21383	0.2609050	-8.49	<.0001*	-3.2583	-1.1694
Gh	Brazil	RIP1	72h	Lh	Uganda	RIP1	24h	0.38707	0.2609050	1.48	0.9999	-0.6574	1.4315
Gh	Brazil	RIP1	72h	Lh	Uganda	RIP1	72h	0.10656	0.2609050	0.41	1.0000	-0.9379	1.1510
Gh	Brazil	RIP1	72h	Lh	Uganda	RIP2	24h	-8.13343	0.2609050	-31.17	<.0001*	-9.1779	-7.0890

Generalized Regression for Value

Standard Least Squares

Multiple Comparisons for Treatment*Strain*RIP*Time

Tukey HSD All Pairwise Comparisons

All Pairwise Differences

Treatment	Strain	RIP	Time	-Treatment	-Strain	-RIP	-Time	Difference	Std Error	t Ratio	Prob>|t|	Lower 95%	Upper 95%
Gh	Brazil	RIP1	72h	Lh	Uganda	RIP2	72h	-8.52392	0.2609050	-32.67	<.0001*	-9.5684	-7.4795
Gh	Brazil	RIP1	72h	Lh	Uganda	RIP3_5	24h	0.12305	0.2609050	0.47	1.0000	-0.9214	1.1675
Gh	Brazil	RIP1	72h	Lh	Uganda	RIP3_5	72h	-1.07578	0.2609050	-4.12	0.0351*	-2.1202	-0.0313
Gh	Brazil	RIP1	72h	no_wasp	Brazil	RIP1	24h	-0.28950	0.2609050	-1.11	1.0000	-1.3339	0.7549
Gh	Brazil	RIP1	72h	no_wasp	Brazil	RIP1	72h	-0.42525	0.2609050	-1.63	0.9995	-1.4697	0.6192
Gh	Brazil	RIP1	72h	no_wasp	Brazil	RIP2	24h	-8.72013	0.2609050	-33.42	<.0001*	-9.7646	-7.6757
Gh	Brazil	RIP1	72h	no_wasp	Brazil	RIP2	72h	-9.04292	0.2609050	-34.66	<.0001*	-10.0874	-7.9985
Gh	Brazil	RIP1	72h	no_wasp	Brazil	RIP3_5	24h	-2.64702	0.2609050	-10.15	<.0001*	-3.6915	-1.6026
Gh	Brazil	RIP1	72h	no_wasp	Brazil	RIP3_5	72h	-2.17770	0.2609050	-8.35	<.0001*	-3.2221	-1.1333
Gh	Brazil	RIP1	72h	no_wasp	Uganda	RIP1	24h	-0.42094	0.2609050	-1.61	0.9996	-1.4654	0.6235
Gh	Brazil	RIP1	72h	no_wasp	Uganda	RIP1	72h	-0.90284	0.2609050	-3.46	0.2029	-1.9473	0.1416
Gh	Brazil	RIP1	72h	no_wasp	Uganda	RIP2	24h	-8.31909	0.2609050	-31.89	<.0001*	-9.3635	-7.2747
Gh	Brazil	RIP1	72h	no_wasp	Uganda	RIP2	72h	-7.66907	0.2609050	-29.39	<.0001*	-8.7135	-6.6246
Gh	Brazil	RIP1	72h	no_wasp	Uganda	RIP3_5	24h	-0.41739	0.2609050	-1.60	0.9996	-1.4618	0.6270
Gh	Brazil	RIP1	72h	no_wasp	Uganda	RIP3_5	72h	-2.24529	0.2609050	-8.61	<.0001*	-3.2897	-1.2009
Gh	Brazil	RIP2	24h	Gh	Brazil	RIP2	72h	-0.13522	0.2609050	-0.52	1.0000	-1.1797	0.9092
Gh	Brazil	RIP2	24h	Gh	Brazil	RIP3_5	24h	7.55354	0.2609050	28.95	<.0001*	6.5091	8.5980
Gh	Brazil	RIP2	24h	Gh	Brazil	RIP3_5	72h	5.67919	0.2609050	21.77	<.0001*	4.6348	6.7236
Gh	Brazil	RIP2	24h	Gh	Uganda	RIP1	24h	9.22122	0.2609050	35.34	<.0001*	8.1768	10.2656
Gh	Brazil	RIP2	24h	Gh	Uganda	RIP1	72h	8.91599	0.2609050	34.17	<.0001*	7.8716	9.9604
Gh	Brazil	RIP2	24h	Gh	Uganda	RIP2	24h	0.35994	0.2609050	1.38	1.0000	-0.6845	1.4044
Gh	Brazil	RIP2	24h	Gh	Uganda	RIP2	72h	-0.09070	0.2609050	-0.35	1.0000	-1.1351	0.9537
Gh	Brazil	RIP2	24h	Gh	Uganda	RIP3_5	24h	9.21636	0.2609050	35.32	<.0001*	8.1719	10.2608
Gh	Brazil	RIP2	24h	Gh	Uganda	RIP3_5	72h	8.44231	0.2609050	32.36	<.0001*	7.3979	9.4867
Gh	Brazil	RIP2	24h	Lh	Brazil	RIP1	24h	9.29119	0.2609050	35.61	<.0001*	8.2468	10.3356
Gh	Brazil	RIP2	24h	Lh	Brazil	RIP1	72h	8.79146	0.2609050	33.70	<.0001*	7.7470	9.8359
Gh	Brazil	RIP2	24h	Lh	Brazil	RIP2	24h	-0.09455	0.2609050	-0.36	1.0000	-1.1390	0.9499
Gh	Brazil	RIP2	24h	Lh	Brazil	RIP2	72h	-0.26136	0.2609050	-1.00	1.0000	-1.3058	0.7831
Gh	Brazil	RIP2	24h	Lh	Brazil	RIP3_5	24h	6.88661	0.2609050	26.40	<.0001*	5.8422	7.9310
Gh	Brazil	RIP2	24h	Lh	Brazil	RIP3_5	72h	6.32019	0.2609050	24.22	<.0001*	5.2758	7.3646
Gh	Brazil	RIP2	24h	Lh	Uganda	RIP1	24h	8.92109	0.2609050	34.19	<.0001*	7.8767	9.9655

Generalized Regression for Value

Standard Least Squares

Multiple Comparisons for Treatment*Strain*RIP*Time

Tukey HSD All Pairwise Comparisons

All Pairwise Differences

Treatment	Strain	RIP	Time	-Treatment	-Strain	-RIP	-Time	Difference	Std Error	t Ratio	Prob>|t|	Lower 95%	Upper 95%
Gh	Brazil	RIP2	24h	Lh	Uganda	RIP1	72h	8.64058	0.2609050	33.12	<.0001*	7.5961	9.6850
Gh	Brazil	RIP2	24h	Lh	Uganda	RIP2	24h	0.40059	0.2609050	1.54	0.9998	-0.6438	1.4450
Gh	Brazil	RIP2	24h	Lh	Uganda	RIP2	72h	0.01010	0.2609050	0.04	1.0000	-1.0343	1.0545
Gh	Brazil	RIP2	24h	Lh	Uganda	RIP3_5	24h	8.65707	0.2609050	33.18	<.0001*	7.6126	9.7015
Gh	Brazil	RIP2	24h	Lh	Uganda	RIP3_5	72h	7.45824	0.2609050	28.59	<.0001*	6.4138	8.5027
Gh	Brazil	RIP2	24h	no_wasp	Brazil	RIP1	24h	8.24452	0.2609050	31.60	<.0001*	7.2001	9.2890
Gh	Brazil	RIP2	24h	no_wasp	Brazil	RIP1	72h	8.10877	0.2609050	31.08	<.0001*	7.0643	9.1532
Gh	Brazil	RIP2	24h	no_wasp	Brazil	RIP2	24h	-0.18610	0.2609050	-0.71	1.0000	-1.2305	0.8583
Gh	Brazil	RIP2	24h	no_wasp	Brazil	RIP2	72h	-0.50890	0.2609050	-1.95	0.9900	-1.5533	0.5355
Gh	Brazil	RIP2	24h	no_wasp	Brazil	RIP3_5	24h	5.88700	0.2609050	22.56	<.0001*	4.8426	6.9314
Gh	Brazil	RIP2	24h	no_wasp	Brazil	RIP3_5	72h	6.35632	0.2609050	24.36	<.0001*	5.3119	7.4007
Gh	Brazil	RIP2	24h	no_wasp	Uganda	RIP1	24h	8.11308	0.2609050	31.10	<.0001*	7.0686	9.1575
Gh	Brazil	RIP2	24h	no_wasp	Uganda	RIP1	72h	7.63118	0.2609050	29.25	<.0001*	6.5867	8.6756
Gh	Brazil	RIP2	24h	no_wasp	Uganda	RIP2	24h	0.21493	0.2609050	0.82	1.0000	-0.8295	1.2594
Gh	Brazil	RIP2	24h	no_wasp	Uganda	RIP2	72h	0.86495	0.2609050	3.32	0.2767	-0.1795	1.9094
Gh	Brazil	RIP2	24h	no_wasp	Uganda	RIP3_5	24h	8.11663	0.2609050	31.11	<.0001*	7.0722	9.1611
Gh	Brazil	RIP2	24h	no_wasp	Uganda	RIP3_5	72h	6.28873	0.2609050	24.10	<.0001*	5.2443	7.3332
Gh	Brazil	RIP2	72h	Gh	Brazil	RIP3_5	24h	7.68876	0.2609050	29.47	<.0001*	6.6443	8.7332
Gh	Brazil	RIP2	72h	Gh	Brazil	RIP3_5	72h	5.81441	0.2609050	22.29	<.0001*	4.7700	6.8588
Gh	Brazil	RIP2	72h	Gh	Uganda	RIP1	24h	9.35644	0.2609050	35.86	<.0001*	8.3120	10.4009
Gh	Brazil	RIP2	72h	Gh	Uganda	RIP1	72h	9.05122	0.2609050	34.69	<.0001*	8.0068	10.0956
Gh	Brazil	RIP2	72h	Gh	Uganda	RIP2	24h	0.49517	0.2609050	1.90	0.9933	-0.5493	1.5396
Gh	Brazil	RIP2	72h	Gh	Uganda	RIP2	72h	0.04452	0.2609050	0.17	1.0000	-0.9999	1.0890
Gh	Brazil	RIP2	72h	Gh	Uganda	RIP3_5	24h	9.35158	0.2609050	35.84	<.0001*	8.3072	10.3960
Gh	Brazil	RIP2	72h	Gh	Uganda	RIP3_5	72h	8.57754	0.2609050	32.88	<.0001*	7.5331	9.6220
Gh	Brazil	RIP2	72h	Lh	Brazil	RIP1	24h	9.42641	0.2609050	36.13	<.0001*	8.3820	10.4708
Gh	Brazil	RIP2	72h	Lh	Brazil	RIP1	72h	8.92668	0.2609050	34.21	<.0001*	7.8823	9.9711
Gh	Brazil	RIP2	72h	Lh	Brazil	RIP2	24h	0.04067	0.2609050	0.16	1.0000	-1.0038	1.0851
Gh	Brazil	RIP2	72h	Lh	Brazil	RIP2	72h	-0.12613	0.2609050	-0.48	1.0000	-1.1706	0.9183
Gh	Brazil	RIP2	72h	Lh	Brazil	RIP3_5	24h	7.02184	0.2609050	26.91	<.0001*	5.9774	8.0663
Gh	Brazil	RIP2	72h	Lh	Brazil	RIP3_5	72h	6.45541	0.2609050	24.74	<.0001*	5.4110	7.4998

Generalized Regression for Value

Standard Least Squares

Multiple Comparisons for Treatment*Strain*RIP*Time

Tukey HSD All Pairwise Comparisons

All Pairwise Differences

Treatment	Strain	RIP	Time	-Treatment	-Strain	-RIP	-Time	Difference	Std Error	t Ratio	Prob>|t|	Lower 95%	Upper 95%
Gh	Brazil	RIP2	72h	Lh	Uganda	RIP1	24h	9.05632	0.2609050	34.71	<.0001*	8.0119	10.1007
Gh	Brazil	RIP2	72h	Lh	Uganda	RIP1	72h	8.77580	0.2609050	33.64	<.0001*	7.7314	9.8202
Gh	Brazil	RIP2	72h	Lh	Uganda	RIP2	24h	0.53581	0.2609050	2.05	0.9798	-0.5086	1.5802
Gh	Brazil	RIP2	72h	Lh	Uganda	RIP2	72h	0.14532	0.2609050	0.56	1.0000	-0.8991	1.1898
Gh	Brazil	RIP2	72h	Lh	Uganda	RIP3_5	24h	8.79229	0.2609050	33.70	<.0001*	7.7479	9.8367
Gh	Brazil	RIP2	72h	Lh	Uganda	RIP3_5	72h	7.59347	0.2609050	29.10	<.0001*	6.5490	8.6379
Gh	Brazil	RIP2	72h	no_wasp	Brazil	RIP1	24h	8.37975	0.2609050	32.12	<.0001*	7.3353	9.4242
Gh	Brazil	RIP2	72h	no_wasp	Brazil	RIP1	72h	8.24400	0.2609050	31.60	<.0001*	7.1996	9.2884
Gh	Brazil	RIP2	72h	no_wasp	Brazil	RIP2	24h	-0.05088	0.2609050	-0.20	1.0000	-1.0953	0.9935
Gh	Brazil	RIP2	72h	no_wasp	Brazil	RIP2	72h	-0.37368	0.2609050	-1.43	1.0000	-1.4181	0.6708
Gh	Brazil	RIP2	72h	no_wasp	Brazil	RIP3_5	24h	6.02222	0.2609050	23.08	<.0001*	4.9778	7.0667
Gh	Brazil	RIP2	72h	no_wasp	Brazil	RIP3_5	72h	6.49154	0.2609050	24.88	<.0001*	5.4471	7.5360
Gh	Brazil	RIP2	72h	no_wasp	Uganda	RIP1	24h	8.24830	0.2609050	31.61	<.0001*	7.2039	9.2927
Gh	Brazil	RIP2	72h	no_wasp	Uganda	RIP1	72h	7.76640	0.2609050	29.77	<.0001*	6.7220	8.8108
Gh	Brazil	RIP2	72h	no_wasp	Uganda	RIP2	24h	0.35016	0.2609050	1.34	1.0000	-0.6943	1.3946
Gh	Brazil	RIP2	72h	no_wasp	Uganda	RIP2	72h	1.00017	0.2609050	3.83	0.0804	-0.0443	2.0446
Gh	Brazil	RIP2	72h	no_wasp	Uganda	RIP3_5	24h	8.25185	0.2609050	31.63	<.0001*	7.2074	9.2963
Gh	Brazil	RIP2	72h	no_wasp	Uganda	RIP3_5	72h	6.42395	0.2609050	24.62	<.0001*	5.3795	7.4684
Gh	Brazil	RIP3_5	24h	Gh	Brazil	RIP3_5	72h	-1.87435	0.2609050	-7.18	<.0001*	-2.9188	-0.8299
Gh	Brazil	RIP3_5	24h	Gh	Uganda	RIP1	24h	1.66768	0.2609050	6.39	<.0001*	0.6232	2.7121
Gh	Brazil	RIP3_5	24h	Gh	Uganda	RIP1	72h	1.36246	0.2609050	5.22	0.0008*	0.3180	2.4069
Gh	Brazil	RIP3_5	24h	Gh	Uganda	RIP2	24h	-7.19359	0.2609050	-27.57	<.0001*	-8.2380	-6.1492
Gh	Brazil	RIP3_5	24h	Gh	Uganda	RIP2	72h	-7.64424	0.2609050	-29.30	<.0001*	-8.6887	-6.5998
Gh	Brazil	RIP3_5	24h	Gh	Uganda	RIP3_5	24h	1.66282	0.2609050	6.37	<.0001*	0.6184	2.7073
Gh	Brazil	RIP3_5	24h	Gh	Uganda	RIP3_5	72h	0.88878	0.2609050	3.41	0.2285	-0.1557	1.9332
Gh	Brazil	RIP3_5	24h	Lh	Brazil	RIP1	24h	1.73766	0.2609050	6.66	<.0001*	0.6932	2.7821
Gh	Brazil	RIP3_5	24h	Lh	Brazil	RIP1	72h	1.23792	0.2609050	4.74	0.0047*	0.1935	2.2824
Gh	Brazil	RIP3_5	24h	Lh	Brazil	RIP2	24h	-7.64809	0.2609050	-29.31	<.0001*	-8.6925	-6.6037
Gh	Brazil	RIP3_5	24h	Lh	Brazil	RIP2	72h	-7.81489	0.2609050	-29.95	<.0001*	-8.8593	-6.7705
Gh	Brazil	RIP3_5	24h	Lh	Brazil	RIP3_5	24h	-0.66692	0.2609050	-2.56	0.8023	-1.7114	0.3775
Gh	Brazil	RIP3_5	24h	Lh	Brazil	RIP3_5	72h	-1.23335	0.2609050	-4.73	0.0049*	-2.2778	-0.1889

Generalized Regression for Value

Standard Least Squares

Multiple Comparisons for Treatment*Strain*RIP*Time

Tukey HSD All Pairwise Comparisons

All Pairwise Differences

Treatment	Strain	RIP	Time	-Treatment	-Strain	-RIP	-Time	Difference	Std Error	t Ratio	Prob>|t|	Lower 95%	Upper 95%
Gh	Brazil	RIP3_5	24h	Lh	Uganda	RIP1	24h	1.36756	0.2609050	5.24	0.0008*	0.3231	2.4120
Gh	Brazil	RIP3_5	24h	Lh	Uganda	RIP1	72h	1.08704	0.2609050	4.17	0.0308*	0.0426	2.1315
Gh	Brazil	RIP3_5	24h	Lh	Uganda	RIP2	24h	-7.15295	0.2609050	-27.42	<.0001*	-8.1974	-6.1085
Gh	Brazil	RIP3_5	24h	Lh	Uganda	RIP2	72h	-7.54344	0.2609050	-28.91	<.0001*	-8.5879	-6.4990
Gh	Brazil	RIP3_5	24h	Lh	Uganda	RIP3_5	24h	1.10353	0.2609050	4.23	0.0254*	0.0591	2.1480
Gh	Brazil	RIP3_5	24h	Lh	Uganda	RIP3_5	72h	-0.09529	0.2609050	-0.37	1.0000	-1.1397	0.9491
Gh	Brazil	RIP3_5	24h	no_wasp	Brazil	RIP1	24h	0.69099	0.2609050	2.65	0.7434	-0.3534	1.7354
Gh	Brazil	RIP3_5	24h	no_wasp	Brazil	RIP1	72h	0.55524	0.2609050	2.13	0.9682	-0.4892	1.5997
Gh	Brazil	RIP3_5	24h	no_wasp	Brazil	RIP2	24h	-7.73964	0.2609050	-29.66	<.0001*	-8.7841	-6.6952
Gh	Brazil	RIP3_5	24h	no_wasp	Brazil	RIP2	72h	-8.06244	0.2609050	-30.90	<.0001*	-9.1069	-7.0180
Gh	Brazil	RIP3_5	24h	no_wasp	Brazil	RIP3_5	24h	-1.66654	0.2609050	-6.39	<.0001*	-2.7110	-0.6221
Gh	Brazil	RIP3_5	24h	no_wasp	Brazil	RIP3_5	72h	-1.19722	0.2609050	-4.59	0.0079*	-2.2416	-0.1528
Gh	Brazil	RIP3_5	24h	no_wasp	Uganda	RIP1	24h	0.55955	0.2609050	2.14	0.9651	-0.4849	1.6040
Gh	Brazil	RIP3_5	24h	no_wasp	Uganda	RIP1	72h	0.07764	0.2609050	0.30	1.0000	-0.9668	1.1221
Gh	Brazil	RIP3_5	24h	no_wasp	Uganda	RIP2	24h	-7.33860	0.2609050	-28.13	<.0001*	-8.3830	-6.2942
Gh	Brazil	RIP3_5	24h	no_wasp	Uganda	RIP2	72h	-6.68858	0.2609050	-25.64	<.0001*	-7.7330	-5.6442
Gh	Brazil	RIP3_5	24h	no_wasp	Uganda	RIP3_5	24h	0.56309	0.2609050	2.16	0.9624	-0.4813	1.6075
Gh	Brazil	RIP3_5	24h	no_wasp	Uganda	RIP3_5	72h	-1.26481	0.2609050	-4.85	0.0032*	-2.3092	-0.2204
Gh	Brazil	RIP3_5	72h	Gh	Uganda	RIP1	24h	3.54203	0.2609050	13.58	<.0001*	2.4976	4.5865
Gh	Brazil	RIP3_5	72h	Gh	Uganda	RIP1	72h	3.23680	0.2609050	12.41	<.0001*	2.1924	4.2812
Gh	Brazil	RIP3_5	72h	Gh	Uganda	RIP2	24h	-5.31925	0.2609050	-20.39	<.0001*	-6.3637	-4.2748
Gh	Brazil	RIP3_5	72h	Gh	Uganda	RIP2	72h	-5.76989	0.2609050	-22.11	<.0001*	-6.8143	-4.7255
Gh	Brazil	RIP3_5	72h	Gh	Uganda	RIP3_5	24h	3.53717	0.2609050	13.56	<.0001*	2.4927	4.5816
Gh	Brazil	RIP3_5	72h	Gh	Uganda	RIP3_5	72h	2.76312	0.2609050	10.59	<.0001*	1.7187	3.8076
Gh	Brazil	RIP3_5	72h	Lh	Brazil	RIP1	24h	3.61200	0.2609050	13.84	<.0001*	2.5676	4.6564
Gh	Brazil	RIP3_5	72h	Lh	Brazil	RIP1	72h	3.11227	0.2609050	11.93	<.0001*	2.0678	4.1567
Gh	Brazil	RIP3_5	72h	Lh	Brazil	RIP2	24h	-5.77374	0.2609050	-22.13	<.0001*	-6.8182	-4.7293
Gh	Brazil	RIP3_5	72h	Lh	Brazil	RIP2	72h	-5.94055	0.2609050	-22.77	<.0001*	-6.9850	-4.8961
Gh	Brazil	RIP3_5	72h	Lh	Brazil	RIP3_5	24h	1.20742	0.2609050	4.63	0.0070*	0.1630	2.2519
Gh	Brazil	RIP3_5	72h	Lh	Brazil	RIP3_5	72h	0.64100	0.2609050	2.46	0.8573	-0.4034	1.6854
Gh	Brazil	RIP3_5	72h	Lh	Uganda	RIP1	24h	3.24190	0.2609050	12.43	<.0001*	2.1975	4.2863

Generalized Regression for Value

Standard Least Squares

Multiple Comparisons for Treatment*Strain*RIP*Time

Tukey HSD All Pairwise Comparisons

All Pairwise Differences

Treatment	Strain	RIP	Time	-Treatment	-Strain	-RIP	-Time	Difference	Std Error	t Ratio	Prob>|t|	Lower 95%	Upper 95%
Gh	Brazil	RIP3_5	72h	Lh	Uganda	RIP1	72h	2.96139	0.2609050	11.35	<.0001*	1.9170	4.0058
Gh	Brazil	RIP3_5	72h	Lh	Uganda	RIP2	24h	-5.27860	0.2609050	-20.23	<.0001*	-6.3230	-4.2342
Gh	Brazil	RIP3_5	72h	Lh	Uganda	RIP2	72h	-5.66909	0.2609050	-21.73	<.0001*	-6.7135	-4.6247
Gh	Brazil	RIP3_5	72h	Lh	Uganda	RIP3_5	24h	2.97788	0.2609050	11.41	<.0001*	1.9334	4.0223
Gh	Brazil	RIP3_5	72h	Lh	Uganda	RIP3_5	72h	1.77905	0.2609050	6.82	<.0001*	0.7346	2.8235
Gh	Brazil	RIP3_5	72h	no_wasp	Brazil	RIP1	24h	2.56533	0.2609050	9.83	<.0001*	1.5209	3.6098
Gh	Brazil	RIP3_5	72h	no_wasp	Brazil	RIP1	72h	2.42958	0.2609050	9.31	<.0001*	1.3852	3.4740
Gh	Brazil	RIP3_5	72h	no_wasp	Brazil	RIP2	24h	-5.86529	0.2609050	-22.48	<.0001*	-6.9097	-4.8209
Gh	Brazil	RIP3_5	72h	no_wasp	Brazil	RIP2	72h	-6.18809	0.2609050	-23.72	<.0001*	-7.2325	-5.1437
Gh	Brazil	RIP3_5	72h	no_wasp	Brazil	RIP3_5	24h	0.20781	0.2609050	0.80	1.0000	-0.8366	1.2522
Gh	Brazil	RIP3_5	72h	no_wasp	Brazil	RIP3_5	72h	0.67713	0.2609050	2.60	0.7781	-0.3673	1.7216
Gh	Brazil	RIP3_5	72h	no_wasp	Uganda	RIP1	24h	2.43389	0.2609050	9.33	<.0001*	1.3895	3.4783
Gh	Brazil	RIP3_5	72h	no_wasp	Uganda	RIP1	72h	1.95199	0.2609050	7.48	<.0001*	0.9076	2.9964
Gh	Brazil	RIP3_5	72h	no_wasp	Uganda	RIP2	24h	-5.46426	0.2609050	-20.94	<.0001*	-6.5087	-4.4198
Gh	Brazil	RIP3_5	72h	no_wasp	Uganda	RIP2	72h	-4.81424	0.2609050	-18.45	<.0001*	-5.8587	-3.7698
Gh	Brazil	RIP3_5	72h	no_wasp	Uganda	RIP3_5	24h	2.43744	0.2609050	9.34	<.0001*	1.3930	3.4819
Gh	Brazil	RIP3_5	72h	no_wasp	Uganda	RIP3_5	72h	0.60954	0.2609050	2.34	0.9107	-0.4349	1.6540
Gh	Uganda	RIP1	24h	Gh	Uganda	RIP1	72h	-0.30522	0.2609050	-1.17	1.0000	-1.3497	0.7392
Gh	Uganda	RIP1	24h	Gh	Uganda	RIP2	24h	-8.86127	0.2609050	-33.96	<.0001*	-9.9057	-7.8168
Gh	Uganda	RIP1	24h	Gh	Uganda	RIP2	72h	-9.31192	0.2609050	-35.69	<.0001*	-10.3563	-8.2675
Gh	Uganda	RIP1	24h	Gh	Uganda	RIP3_5	24h	-0.00486	0.2609050	-0.02	1.0000	-1.0493	1.0396
Gh	Uganda	RIP1	24h	Gh	Uganda	RIP3_5	72h	-0.77890	0.2609050	-2.99	0.4960	-1.8233	0.2655
Gh	Uganda	RIP1	24h	Lh	Brazil	RIP1	24h	0.06998	0.2609050	0.27	1.0000	-0.9745	1.1144
Gh	Uganda	RIP1	24h	Lh	Brazil	RIP1	72h	-0.42976	0.2609050	-1.65	0.9994	-1.4742	0.6147
Gh	Uganda	RIP1	24h	Lh	Brazil	RIP2	24h	-9.31577	0.2609050	-35.71	<.0001*	-10.3602	-8.2713
Gh	Uganda	RIP1	24h	Lh	Brazil	RIP2	72h	-9.48257	0.2609050	-36.34	<.0001*	-10.5270	-8.4381
Gh	Uganda	RIP1	24h	Lh	Brazil	RIP3_5	24h	-2.33460	0.2609050	-8.95	<.0001*	-3.3790	-1.2902
Gh	Uganda	RIP1	24h	Lh	Brazil	RIP3_5	72h	-2.90103	0.2609050	-11.12	<.0001*	-3.9455	-1.8566
Gh	Uganda	RIP1	24h	Lh	Uganda	RIP1	24h	-0.30012	0.2609050	-1.15	1.0000	-1.3446	0.7443
Gh	Uganda	RIP1	24h	Lh	Uganda	RIP1	72h	-0.58064	0.2609050	-2.23	0.9464	-1.6251	0.4638
Gh	Uganda	RIP1	24h	Lh	Uganda	RIP2	24h	-8.82063	0.2609050	-33.81	<.0001*	-9.8651	-7.7762

Generalized Regression for Value
Standard Least Squares
Multiple Comparisons for Treatment*Strain*RIP*Time
Tukey HSD All Pairwise Comparisons
All Pairwise Differences

Treatment	Strain	RIP	Time	-Treatment	-Strain	-RIP	-Time	Difference	Std Error	t Ratio	Prob>|t|	Lower 95%	Upper 95%
Gh	Uganda	RIP1	24h	Lh	Uganda	RIP2	72h	-9.21112	0.2609050	-35.30	<.0001*	-10.2555	-8.1667
Gh	Uganda	RIP1	24h	Lh	Uganda	RIP3_5	24h	-0.56415	0.2609050	-2.16	0.9615	-1.6086	0.4803
Gh	Uganda	RIP1	24h	Lh	Uganda	RIP3_5	72h	-1.76297	0.2609050	-6.76	<.0001*	-2.8074	-0.7185
Gh	Uganda	RIP1	24h	no_wasp	Brazil	RIP1	24h	-0.97669	0.2609050	-3.74	0.1020	-2.0211	0.0677
Gh	Uganda	RIP1	24h	no_wasp	Brazil	RIP1	72h	-1.11244	0.2609050	-4.26	0.0228*	-2.1569	-0.0680
Gh	Uganda	RIP1	24h	no_wasp	Brazil	RIP2	24h	-9.40732	0.2609050	-36.06	<.0001*	-10.4518	-8.3629
Gh	Uganda	RIP1	24h	no_wasp	Brazil	RIP2	72h	-9.73012	0.2609050	-37.29	<.0001*	-10.7745	-8.6857
Gh	Uganda	RIP1	24h	no_wasp	Brazil	RIP3_5	24h	-3.33422	0.2609050	-12.78	<.0001*	-4.3786	-2.2898
Gh	Uganda	RIP1	24h	no_wasp	Brazil	RIP3_5	72h	-2.86490	0.2609050	-10.98	<.0001*	-3.9093	-1.8205
Gh	Uganda	RIP1	24h	no_wasp	Uganda	RIP1	24h	-1.10813	0.2609050	-4.25	0.0240*	-2.1526	-0.0637
Gh	Uganda	RIP1	24h	no_wasp	Uganda	RIP1	72h	-1.59004	0.2609050	-6.09	<.0001*	-2.6345	-0.5456
Gh	Uganda	RIP1	24h	no_wasp	Uganda	RIP2	24h	-9.00628	0.2609050	-34.52	<.0001*	-10.0507	-7.9618
Gh	Uganda	RIP1	24h	no_wasp	Uganda	RIP2	72h	-8.35626	0.2609050	-32.03	<.0001*	-9.4007	-7.3118
Gh	Uganda	RIP1	24h	no_wasp	Uganda	RIP3_5	24h	-1.10459	0.2609050	-4.23	0.0251*	-2.1490	-0.0602
Gh	Uganda	RIP1	24h	no_wasp	Uganda	RIP3_5	72h	-2.93249	0.2609050	-11.24	<.0001*	-3.9769	-1.8881
Gh	Uganda	RIP1	72h	Gh	Uganda	RIP2	24h	-8.55605	0.2609050	-32.79	<.0001*	-9.6005	-7.5116
Gh	Uganda	RIP1	72h	Gh	Uganda	RIP2	72h	-9.00669	0.2609050	-34.52	<.0001*	-10.0511	-7.9623
Gh	Uganda	RIP1	72h	Gh	Uganda	RIP3_5	24h	0.30037	0.2609050	1.15	1.0000	-0.7441	1.3448
Gh	Uganda	RIP1	72h	Gh	Uganda	RIP3_5	72h	-0.47368	0.2609050	-1.82	0.9967	-1.5181	0.5708
Gh	Uganda	RIP1	72h	Lh	Brazil	RIP1	24h	0.37520	0.2609050	1.44	1.0000	-0.6692	1.4196
Gh	Uganda	RIP1	72h	Lh	Brazil	RIP1	72h	-0.12453	0.2609050	-0.48	1.0000	-1.1690	0.9199
Gh	Uganda	RIP1	72h	Lh	Brazil	RIP2	24h	-9.01054	0.2609050	-34.54	<.0001*	-10.0550	-7.9661
Gh	Uganda	RIP1	72h	Lh	Brazil	RIP2	72h	-9.17735	0.2609050	-35.18	<.0001*	-10.2218	-8.1329
Gh	Uganda	RIP1	72h	Lh	Brazil	RIP3_5	24h	-2.02938	0.2609050	-7.78	<.0001*	-3.0738	-0.9849
Gh	Uganda	RIP1	72h	Lh	Brazil	RIP3_5	72h	-2.59580	0.2609050	-9.95	<.0001*	-3.6402	-1.5514
Gh	Uganda	RIP1	72h	Lh	Uganda	RIP1	24h	0.00510	0.2609050	0.02	1.0000	-1.0393	1.0495
Gh	Uganda	RIP1	72h	Lh	Uganda	RIP1	72h	-0.27541	0.2609050	-1.06	1.0000	-1.3198	0.7690
Gh	Uganda	RIP1	72h	Lh	Uganda	RIP2	24h	-8.51540	0.2609050	-32.64	<.0001*	-9.5598	-7.4710
Gh	Uganda	RIP1	72h	Lh	Uganda	RIP2	72h	-8.90589	0.2609050	-34.13	<.0001*	-9.9503	-7.8615
Gh	Uganda	RIP1	72h	Lh	Uganda	RIP3_5	24h	-0.25892	0.2609050	-0.99	1.0000	-1.3034	0.7855
Gh	Uganda	RIP1	72h	Lh	Uganda	RIP3_5	72h	-1.45775	0.2609050	-5.59	0.0002*	-2.5022	-0.4133

Generalized Regression for Value

Standard Least Squares

Multiple Comparisons for Treatment*Strain*RIP*Time

Tukey HSD All Pairwise Comparisons

All Pairwise Differences

Treatment	Strain	RIP	Time	-Treatment	-Strain	-RIP	-Time	Difference	Std Error	t Ratio	Prob>|t|	Lower 95%	Upper 95%
Gh	Uganda	RIP1	72h	no_wasp	Brazil	RIP1	24h	-0.67147	0.2609050	-2.57	0.7917	-1.7159	0.3730
Gh	Uganda	RIP1	72h	no_wasp	Brazil	RIP1	72h	-0.80722	0.2609050	-3.09	0.4175	-1.8517	0.2372
Gh	Uganda	RIP1	72h	no_wasp	Brazil	RIP2	24h	-9.10210	0.2609050	-34.89	<.0001*	-10.1465	-8.0577
Gh	Uganda	RIP1	72h	no_wasp	Brazil	RIP2	72h	-9.42489	0.2609050	-36.12	<.0001*	-10.4693	-8.3805
Gh	Uganda	RIP1	72h	no_wasp	Brazil	RIP3_5	24h	-3.02899	0.2609050	-11.61	<.0001*	-4.0734	-1.9846
Gh	Uganda	RIP1	72h	no_wasp	Brazil	RIP3_5	72h	-2.55967	0.2609050	-9.81	<.0001*	-3.6041	-1.5152
Gh	Uganda	RIP1	72h	no_wasp	Uganda	RIP1	24h	-0.80291	0.2609050	-3.08	0.4291	-1.8473	0.2415
Gh	Uganda	RIP1	72h	no_wasp	Uganda	RIP1	72h	-1.28481	0.2609050	-4.92	0.0025*	-2.3292	-0.2404
Gh	Uganda	RIP1	72h	no_wasp	Uganda	RIP2	24h	-8.70106	0.2609050	-33.35	<.0001*	-9.7455	-7.6566
Gh	Uganda	RIP1	72h	no_wasp	Uganda	RIP2	72h	-8.05104	0.2609050	-30.86	<.0001*	-9.0955	-7.0066
Gh	Uganda	RIP1	72h	no_wasp	Uganda	RIP3_5	24h	-0.79936	0.2609050	-3.06	0.4388	-1.8438	0.2451
Gh	Uganda	RIP1	72h	no_wasp	Uganda	RIP3_5	72h	-2.62726	0.2609050	-10.07	<.0001*	-3.6717	-1.5828
Gh	Uganda	RIP2	24h	Gh	Uganda	RIP2	72h	-0.45064	0.2609050	-1.73	0.9985	-1.4951	0.5938
Gh	Uganda	RIP2	24h	Gh	Uganda	RIP3_5	24h	8.85642	0.2609050	33.94	<.0001*	7.8120	9.9008
Gh	Uganda	RIP2	24h	Gh	Uganda	RIP3_5	72h	8.08237	0.2609050	30.98	<.0001*	7.0379	9.1268
Gh	Uganda	RIP2	24h	Lh	Brazil	RIP1	24h	8.93125	0.2609050	34.23	<.0001*	7.8868	9.9757
Gh	Uganda	RIP2	24h	Lh	Brazil	RIP1	72h	8.43152	0.2609050	32.32	<.0001*	7.3871	9.4759
Gh	Uganda	RIP2	24h	Lh	Brazil	RIP2	24h	-0.45449	0.2609050	-1.74	0.9983	-1.4989	0.5899
Gh	Uganda	RIP2	24h	Lh	Brazil	RIP2	72h	-0.62130	0.2609050	-2.38	0.8925	-1.6657	0.4231
Gh	Uganda	RIP2	24h	Lh	Brazil	RIP3_5	24h	6.52667	0.2609050	25.02	<.0001*	5.4822	7.5711
Gh	Uganda	RIP2	24h	Lh	Brazil	RIP3_5	72h	5.96025	0.2609050	22.84	<.0001*	4.9158	7.0047
Gh	Uganda	RIP2	24h	Lh	Uganda	RIP1	24h	8.56115	0.2609050	32.81	<.0001*	7.5167	9.6056
Gh	Uganda	RIP2	24h	Lh	Uganda	RIP1	72h	8.28064	0.2609050	31.74	<.0001*	7.2362	9.3251
Gh	Uganda	RIP2	24h	Lh	Uganda	RIP2	24h	0.04065	0.2609050	0.16	1.0000	-1.0038	1.0851
Gh	Uganda	RIP2	24h	Lh	Uganda	RIP2	72h	-0.34984	0.2609050	-1.34	1.0000	-1.3943	0.6946
Gh	Uganda	RIP2	24h	Lh	Uganda	RIP3_5	24h	8.29713	0.2609050	31.80	<.0001*	7.2527	9.3416
Gh	Uganda	RIP2	24h	Lh	Uganda	RIP3_5	72h	7.09830	0.2609050	27.21	<.0001*	6.0539	8.1427
Gh	Uganda	RIP2	24h	no_wasp	Brazil	RIP1	24h	7.88458	0.2609050	30.22	<.0001*	6.8402	8.9290
Gh	Uganda	RIP2	24h	no_wasp	Brazil	RIP1	72h	7.74883	0.2609050	29.70	<.0001*	6.7044	8.7933
Gh	Uganda	RIP2	24h	no_wasp	Brazil	RIP2	24h	-0.54605	0.2609050	-2.09	0.9742	-1.5905	0.4984
Gh	Uganda	RIP2	24h	no_wasp	Brazil	RIP2	72h	-0.86884	0.2609050	-3.33	0.2684	-1.9133	0.1756

Generalized Regression for Value
Standard Least Squares
Multiple Comparisons for Treatment*Strain*RIP*Time
Tukey HSD All Pairwise Comparisons
All Pairwise Differences

Treatment	Strain	RIP	Time	-Treatment	-Strain	-RIP	-Time	Difference	Std Error	t Ratio	Prob>|t|	Lower 95%	Upper 95%
Gh	Uganda	RIP2	24h	no_wasp	Brazil	RIP3_5	24h	5.52706	0.2609050	21.18	<.0001*	4.4826	6.5715
Gh	Uganda	RIP2	24h	no_wasp	Brazil	RIP3_5	72h	5.99638	0.2609050	22.98	<.0001*	4.9519	7.0408
Gh	Uganda	RIP2	24h	no_wasp	Uganda	RIP1	24h	7.75314	0.2609050	29.72	<.0001*	6.7087	8.7976
Gh	Uganda	RIP2	24h	no_wasp	Uganda	RIP1	72h	7.27124	0.2609050	27.87	<.0001*	6.2268	8.3157
Gh	Uganda	RIP2	24h	no_wasp	Uganda	RIP2	24h	-0.14501	0.2609050	-0.56	1.0000	-1.1894	0.8994
Gh	Uganda	RIP2	24h	no_wasp	Uganda	RIP2	72h	0.50501	0.2609050	1.94	0.9911	-0.5394	1.5494
Gh	Uganda	RIP2	24h	no_wasp	Uganda	RIP3_5	24h	7.75669	0.2609050	29.73	<.0001*	6.7123	8.8011
Gh	Uganda	RIP2	24h	no_wasp	Uganda	RIP3_5	72h	5.92879	0.2609050	22.72	<.0001*	4.8844	6.9732
Gh	Uganda	RIP2	72h	Gh	Uganda	RIP3_5	24h	9.30706	0.2609050	35.67	<.0001*	8.2626	10.3515
Gh	Uganda	RIP2	72h	Gh	Uganda	RIP3_5	72h	8.53302	0.2609050	32.71	<.0001*	7.4886	9.5774
Gh	Uganda	RIP2	72h	Lh	Brazil	RIP1	24h	9.38189	0.2609050	35.96	<.0001*	8.3375	10.4263
Gh	Uganda	RIP2	72h	Lh	Brazil	RIP1	72h	8.88216	0.2609050	34.04	<.0001*	7.8377	9.9266
Gh	Uganda	RIP2	72h	Lh	Brazil	RIP2	24h	-0.00385	0.2609050	-0.01	1.0000	-1.0483	1.0406
Gh	Uganda	RIP2	72h	Lh	Brazil	RIP2	72h	-0.17065	0.2609050	-0.65	1.0000	-1.2151	0.8738
Gh	Uganda	RIP2	72h	Lh	Brazil	RIP3_5	24h	6.97732	0.2609050	26.74	<.0001*	5.9329	8.0217
Gh	Uganda	RIP2	72h	Lh	Brazil	RIP3_5	72h	6.41089	0.2609050	24.57	<.0001*	5.3665	7.4553
Gh	Uganda	RIP2	72h	Lh	Uganda	RIP1	24h	9.01180	0.2609050	34.54	<.0001*	7.9674	10.0562
Gh	Uganda	RIP2	72h	Lh	Uganda	RIP1	72h	8.73128	0.2609050	33.47	<.0001*	7.6868	9.7757
Gh	Uganda	RIP2	72h	Lh	Uganda	RIP2	24h	0.49129	0.2609050	1.88	0.9941	-0.5531	1.5357
Gh	Uganda	RIP2	72h	Lh	Uganda	RIP2	72h	0.10080	0.2609050	0.39	1.0000	-0.9436	1.1452
Gh	Uganda	RIP2	72h	Lh	Uganda	RIP3_5	24h	8.74777	0.2609050	33.53	<.0001*	7.7033	9.7922
Gh	Uganda	RIP2	72h	Lh	Uganda	RIP3_5	72h	7.54894	0.2609050	28.93	<.0001*	6.5045	8.5934
Gh	Uganda	RIP2	72h	no_wasp	Brazil	RIP1	24h	8.33523	0.2609050	31.95	<.0001*	7.2908	9.3797
Gh	Uganda	RIP2	72h	no_wasp	Brazil	RIP1	72h	8.19948	0.2609050	31.43	<.0001*	7.1550	9.2439
Gh	Uganda	RIP2	72h	no_wasp	Brazil	RIP2	24h	-0.09540	0.2609050	-0.37	1.0000	-1.1398	0.9490
Gh	Uganda	RIP2	72h	no_wasp	Brazil	RIP2	72h	-0.41820	0.2609050	-1.60	0.9996	-1.4626	0.6262
Gh	Uganda	RIP2	72h	no_wasp	Brazil	RIP3_5	24h	5.97770	0.2609050	22.91	<.0001*	4.9333	7.0221
Gh	Uganda	RIP2	72h	no_wasp	Brazil	RIP3_5	72h	6.44702	0.2609050	24.71	<.0001*	5.4026	7.4915
Gh	Uganda	RIP2	72h	no_wasp	Uganda	RIP1	24h	8.20378	0.2609050	31.44	<.0001*	7.1594	9.2482
Gh	Uganda	RIP2	72h	no_wasp	Uganda	RIP1	72h	7.72188	0.2609050	29.60	<.0001*	6.6774	8.7663
Gh	Uganda	RIP2	72h	no_wasp	Uganda	RIP2	24h	0.30564	0.2609050	1.17	1.0000	-0.7388	1.3501

Generalized Regression for Value

Standard Least Squares

Multiple Comparisons for Treatment*Strain*RIP*Time

Tukey HSD All Pairwise Comparisons

All Pairwise Differences

Treatment	Strain	RIP	Time	-Treatment	-Strain	-RIP	-Time	Difference	Std Error	t Ratio	Prob>|t|	Lower 95%	Upper 95%
Gh	Uganda	RIP2	72h	no_wasp	Uganda	RIP2	72h	0.95565	0.2609050	3.66	0.1254	-0.0888	2.0001
Gh	Uganda	RIP2	72h	no_wasp	Uganda	RIP3_5	24h	8.20733	0.2609050	31.46	<.0001*	7.1629	9.2518
Gh	Uganda	RIP2	72h	no_wasp	Uganda	RIP3_5	72h	6.37943	0.2609050	24.45	<.0001*	5.3350	7.4239
Gh	Uganda	RIP3_5	24h	Gh	Uganda	RIP3_5	72h	-0.77405	0.2609050	-2.97	0.5099	-1.8185	0.2704
Gh	Uganda	RIP3_5	24h	Lh	Brazil	RIP1	24h	0.07483	0.2609050	0.29	1.0000	-0.9696	1.1193
Gh	Uganda	RIP3_5	24h	Lh	Brazil	RIP1	72h	-0.42490	0.2609050	-1.63	0.9995	-1.4693	0.6195
Gh	Uganda	RIP3_5	24h	Lh	Brazil	RIP2	24h	-9.31091	0.2609050	-35.69	<.0001*	-10.3553	-8.2665
Gh	Uganda	RIP3_5	24h	Lh	Brazil	RIP2	72h	-9.47772	0.2609050	-36.33	<.0001*	-10.5221	-8.4333
Gh	Uganda	RIP3_5	24h	Lh	Brazil	RIP3_5	24h	-2.32975	0.2609050	-8.93	<.0001*	-3.3742	-1.2853
Gh	Uganda	RIP3_5	24h	Lh	Brazil	RIP3_5	72h	-2.89617	0.2609050	-11.10	<.0001*	-3.9406	-1.8517
Gh	Uganda	RIP3_5	24h	Lh	Uganda	RIP1	24h	-0.29527	0.2609050	-1.13	1.0000	-1.3397	0.7492
Gh	Uganda	RIP3_5	24h	Lh	Uganda	RIP1	72h	-0.57578	0.2609050	-2.21	0.9512	-1.6202	0.4686
Gh	Uganda	RIP3_5	24h	Lh	Uganda	RIP2	24h	-8.81577	0.2609050	-33.79	<.0001*	-9.8602	-7.7713
Gh	Uganda	RIP3_5	24h	Lh	Uganda	RIP2	72h	-9.20626	0.2609050	-35.29	<.0001*	-10.2507	-8.1618
Gh	Uganda	RIP3_5	24h	Lh	Uganda	RIP3_5	24h	-0.55929	0.2609050	-2.14	0.9653	-1.6037	0.4851
Gh	Uganda	RIP3_5	24h	Lh	Uganda	RIP3_5	72h	-1.75812	0.2609050	-6.74	<.0001*	-2.8025	-0.7137
Gh	Uganda	RIP3_5	24h	no_wasp	Brazil	RIP1	24h	-0.97184	0.2609050	-3.72	0.1071	-2.0163	0.0726
Gh	Uganda	RIP3_5	24h	no_wasp	Brazil	RIP1	72h	-1.10759	0.2609050	-4.25	0.0242*	-2.1520	-0.0632
Gh	Uganda	RIP3_5	24h	no_wasp	Brazil	RIP2	24h	-9.40246	0.2609050	-36.04	<.0001*	-10.4469	-8.3580
Gh	Uganda	RIP3_5	24h	no_wasp	Brazil	RIP2	72h	-9.72526	0.2609050	-37.28	<.0001*	-10.7697	-8.6808
Gh	Uganda	RIP3_5	24h	no_wasp	Brazil	RIP3_5	24h	-3.32936	0.2609050	-12.76	<.0001*	-4.3738	-2.2849
Gh	Uganda	RIP3_5	24h	no_wasp	Brazil	RIP3_5	72h	-2.86004	0.2609050	-10.96	<.0001*	-3.9045	-1.8156
Gh	Uganda	RIP3_5	24h	no_wasp	Uganda	RIP1	24h	-1.10328	0.2609050	-4.23	0.0255*	-2.1477	-0.0588
Gh	Uganda	RIP3_5	24h	no_wasp	Uganda	RIP1	72h	-1.58518	0.2609050	-6.08	<.0001*	-2.6296	-0.5408
Gh	Uganda	RIP3_5	24h	no_wasp	Uganda	RIP2	24h	-9.00143	0.2609050	-34.50	<.0001*	-10.0459	-7.9570
Gh	Uganda	RIP3_5	24h	no_wasp	Uganda	RIP2	72h	-8.35141	0.2609050	-32.01	<.0001*	-9.3958	-7.3070
Gh	Uganda	RIP3_5	24h	no_wasp	Uganda	RIP3_5	24h	-1.09973	0.2609050	-4.22	0.0266*	-2.1442	-0.0553
Gh	Uganda	RIP3_5	24h	no_wasp	Uganda	RIP3_5	72h	-2.92763	0.2609050	-11.22	<.0001*	-3.9721	-1.8832
Gh	Uganda	RIP3_5	72h	Lh	Brazil	RIP1	24h	0.84888	0.2609050	3.25	0.3127	-0.1956	1.8933
Gh	Uganda	RIP3_5	72h	Lh	Brazil	RIP1	72h	0.34915	0.2609050	1.34	1.0000	-0.6953	1.3936
Gh	Uganda	RIP3_5	72h	Lh	Brazil	RIP2	24h	-8.53686	0.2609050	-32.72	<.0001*	-9.5813	-7.4924

Generalized Regression for Value

Standard Least Squares

Multiple Comparisons for Treatment*Strain*RIP*Time

Tukey HSD All Pairwise Comparisons

All Pairwise Differences

Treatment	Strain	RIP	Time	-Treatment	-Strain	-RIP	-Time	Difference	Std Error	t Ratio	Prob>|t|	Lower 95%	Upper 95%
Gh	Uganda	RIP3_5	72h	Lh	Brazil	RIP2	72h	-8.70367	0.2609050	-33.36	<.0001*	-9.7481	-7.6592
Gh	Uganda	RIP3_5	72h	Lh	Brazil	RIP3_5	24h	-1.55570	0.2609050	-5.96	<.0001*	-2.6001	-0.5113
Gh	Uganda	RIP3_5	72h	Lh	Brazil	RIP3_5	72h	-2.12212	0.2609050	-8.13	<.0001*	-3.1666	-1.0777
Gh	Uganda	RIP3_5	72h	Lh	Uganda	RIP1	24h	0.47878	0.2609050	1.84	0.9960	-0.5657	1.5232
Gh	Uganda	RIP3_5	72h	Lh	Uganda	RIP1	72h	0.19826	0.2609050	0.76	1.0000	-0.8462	1.2427
Gh	Uganda	RIP3_5	72h	Lh	Uganda	RIP2	24h	-8.04172	0.2609050	-30.82	<.0001*	-9.0862	-6.9973
Gh	Uganda	RIP3_5	72h	Lh	Uganda	RIP2	72h	-8.43221	0.2609050	-32.32	<.0001*	-9.4766	-7.3878
Gh	Uganda	RIP3_5	72h	Lh	Uganda	RIP3_5	24h	0.21476	0.2609050	0.82	1.0000	-0.8297	1.2592
Gh	Uganda	RIP3_5	72h	Lh	Uganda	RIP3_5	72h	-0.98407	0.2609050	-3.77	0.0948	-2.0285	0.0604
Gh	Uganda	RIP3_5	72h	no_wasp	Brazil	RIP1	24h	-0.19779	0.2609050	-0.76	1.0000	-1.2422	0.8466
Gh	Uganda	RIP3_5	72h	no_wasp	Brazil	RIP1	72h	-0.33354	0.2609050	-1.28	1.0000	-1.3780	0.7109
Gh	Uganda	RIP3_5	72h	no_wasp	Brazil	RIP2	24h	-8.62842	0.2609050	-33.07	<.0001*	-9.6728	-7.5840
Gh	Uganda	RIP3_5	72h	no_wasp	Brazil	RIP2	72h	-8.95121	0.2609050	-34.31	<.0001*	-9.9956	-7.9068
Gh	Uganda	RIP3_5	72h	no_wasp	Brazil	RIP3_5	24h	-2.55531	0.2609050	-9.79	<.0001*	-3.5997	-1.5109
Gh	Uganda	RIP3_5	72h	no_wasp	Brazil	RIP3_5	72h	-2.08599	0.2609050	-8.00	<.0001*	-3.1304	-1.0416
Gh	Uganda	RIP3_5	72h	no_wasp	Uganda	RIP1	24h	-0.32923	0.2609050	-1.26	1.0000	-1.3737	0.7152
Gh	Uganda	RIP3_5	72h	no_wasp	Uganda	RIP1	72h	-0.81113	0.2609050	-3.11	0.4070	-1.8556	0.2333
Gh	Uganda	RIP3_5	72h	no_wasp	Uganda	RIP2	24h	-8.22738	0.2609050	-31.53	<.0001*	-9.2718	-7.1829
Gh	Uganda	RIP3_5	72h	no_wasp	Uganda	RIP2	72h	-7.57736	0.2609050	-29.04	<.0001*	-8.6218	-6.5329
Gh	Uganda	RIP3_5	72h	no_wasp	Uganda	RIP3_5	24h	-0.32568	0.2609050	-1.25	1.0000	-1.3701	0.7187
Gh	Uganda	RIP3_5	72h	no_wasp	Uganda	RIP3_5	72h	-2.15358	0.2609050	-8.25	<.0001*	-3.1980	-1.1092
Lh	Brazil	RIP1	24h	Lh	Brazil	RIP1	72h	-0.49973	0.2609050	-1.92	0.9923	-1.5442	0.5447
Lh	Brazil	RIP1	24h	Lh	Brazil	RIP2	24h	-9.38574	0.2609050	-35.97	<.0001*	-10.4302	-8.3413
Lh	Brazil	RIP1	24h	Lh	Brazil	RIP2	72h	-9.55255	0.2609050	-36.61	<.0001*	-10.5970	-8.5081
Lh	Brazil	RIP1	24h	Lh	Brazil	RIP3_5	24h	-2.40458	0.2609050	-9.22	<.0001*	-3.4490	-1.3601
Lh	Brazil	RIP1	24h	Lh	Brazil	RIP3_5	72h	-2.97100	0.2609050	-11.39	<.0001*	-4.0154	-1.9266
Lh	Brazil	RIP1	24h	Lh	Uganda	RIP1	24h	-0.37010	0.2609050	-1.42	1.0000	-1.4145	0.6743
Lh	Brazil	RIP1	24h	Lh	Uganda	RIP1	72h	-0.65061	0.2609050	-2.49	0.8380	-1.6950	0.3938
Lh	Brazil	RIP1	24h	Lh	Uganda	RIP2	24h	-8.89060	0.2609050	-34.08	<.0001*	-9.9350	-7.8462
Lh	Brazil	RIP1	24h	Lh	Uganda	RIP2	72h	-9.28109	0.2609050	-35.57	<.0001*	-10.3255	-8.2367
Lh	Brazil	RIP1	24h	Lh	Uganda	RIP3_5	24h	-0.63412	0.2609050	-2.43	0.8703	-1.6786	0.4103

Generalized Regression for Value

Standard Least Squares

Multiple Comparisons for Treatment*Strain*RIP*Time

Tukey HSD All Pairwise Comparisons

All Pairwise Differences

Treatment	Strain	RIP	Time	-Treatment	-Strain	-RIP	-Time	Difference	Std Error	t Ratio	Prob>|t|	Lower 95%	Upper 95%
Lh	Brazil	RIP1	24h	Lh	Uganda	RIP3_5	72h	-1.83295	0.2609050	-7.03	<.0001*	-2.8774	-0.7885
Lh	Brazil	RIP1	24h	no_wasp	Brazil	RIP1	24h	-1.04667	0.2609050	-4.01	0.0488*	-2.0911	-0.0022
Lh	Brazil	RIP1	24h	no_wasp	Brazil	RIP1	72h	-1.18242	0.2609050	-4.53	0.0096*	-2.2269	-0.1380
Lh	Brazil	RIP1	24h	no_wasp	Brazil	RIP2	24h	-9.47730	0.2609050	-36.32	<.0001*	-10.5217	-8.4329
Lh	Brazil	RIP1	24h	no_wasp	Brazil	RIP2	72h	-9.80009	0.2609050	-37.56	<.0001*	-10.8445	-8.7557
Lh	Brazil	RIP1	24h	no_wasp	Brazil	RIP3_5	24h	-3.40419	0.2609050	-13.05	<.0001*	-4.4486	-2.3598
Lh	Brazil	RIP1	24h	no_wasp	Brazil	RIP3_5	72h	-2.93487	0.2609050	-11.25	<.0001*	-3.9793	-1.8904
Lh	Brazil	RIP1	24h	no_wasp	Uganda	RIP1	24h	-1.17811	0.2609050	-4.52	0.0101*	-2.2225	-0.1337
Lh	Brazil	RIP1	24h	no_wasp	Uganda	RIP1	72h	-1.66001	0.2609050	-6.36	<.0001*	-2.7044	-0.6156
Lh	Brazil	RIP1	24h	no_wasp	Uganda	RIP2	24h	-9.07626	0.2609050	-34.79	<.0001*	-10.1207	-8.0318
Lh	Brazil	RIP1	24h	no_wasp	Uganda	RIP2	72h	-8.42624	0.2609050	-32.30	<.0001*	-9.4707	-7.3818
Lh	Brazil	RIP1	24h	no_wasp	Uganda	RIP3_5	24h	-1.17456	0.2609050	-4.50	0.0106*	-2.2190	-0.1301
Lh	Brazil	RIP1	24h	no_wasp	Uganda	RIP3_5	72h	-3.00246	0.2609050	-11.51	<.0001*	-4.0469	-1.9580
Lh	Brazil	RIP1	72h	Lh	Brazil	RIP2	24h	-8.88601	0.2609050	-34.06	<.0001*	-9.9304	-7.8416
Lh	Brazil	RIP1	72h	Lh	Brazil	RIP2	72h	-9.05282	0.2609050	-34.70	<.0001*	-10.0972	-8.0084
Lh	Brazil	RIP1	72h	Lh	Brazil	RIP3_5	24h	-1.90485	0.2609050	-7.30	<.0001*	-2.9493	-0.8604
Lh	Brazil	RIP1	72h	Lh	Brazil	RIP3_5	72h	-2.47127	0.2609050	-9.47	<.0001*	-3.5157	-1.4268
Lh	Brazil	RIP1	72h	Lh	Uganda	RIP1	24h	0.12963	0.2609050	0.50	1.0000	-0.9148	1.1741
Lh	Brazil	RIP1	72h	Lh	Uganda	RIP1	72h	-0.15088	0.2609050	-0.58	1.0000	-1.1953	0.8935
Lh	Brazil	RIP1	72h	Lh	Uganda	RIP2	24h	-8.39087	0.2609050	-32.16	<.0001*	-9.4353	-7.3464
Lh	Brazil	RIP1	72h	Lh	Uganda	RIP2	72h	-8.78136	0.2609050	-33.66	<.0001*	-9.8258	-7.7369
Lh	Brazil	RIP1	72h	Lh	Uganda	RIP3_5	24h	-0.13439	0.2609050	-0.52	1.0000	-1.1788	0.9100
Lh	Brazil	RIP1	72h	Lh	Uganda	RIP3_5	72h	-1.33322	0.2609050	-5.11	0.0013*	-2.3777	-0.2888
Lh	Brazil	RIP1	72h	no_wasp	Brazil	RIP1	24h	-0.54694	0.2609050	-2.10	0.9736	-1.5914	0.4975
Lh	Brazil	RIP1	72h	no_wasp	Brazil	RIP1	72h	-0.68269	0.2609050	-2.62	0.7644	-1.7271	0.3617
Lh	Brazil	RIP1	72h	no_wasp	Brazil	RIP2	24h	-8.97757	0.2609050	-34.41	<.0001*	-10.0220	-7.9331
Lh	Brazil	RIP1	72h	no_wasp	Brazil	RIP2	72h	-9.30036	0.2609050	-35.65	<.0001*	-10.3448	-8.2559
Lh	Brazil	RIP1	72h	no_wasp	Brazil	RIP3_5	24h	-2.90446	0.2609050	-11.13	<.0001*	-3.9489	-1.8600
Lh	Brazil	RIP1	72h	no_wasp	Brazil	RIP3_5	72h	-2.43514	0.2609050	-9.33	<.0001*	-3.4796	-1.3907
Lh	Brazil	RIP1	72h	no_wasp	Uganda	RIP1	24h	-0.67838	0.2609050	-2.60	0.7751	-1.7228	0.3661
Lh	Brazil	RIP1	72h	no_wasp	Uganda	RIP1	72h	-1.16028	0.2609050	-4.45	0.0127*	-2.2047	-0.1159

Generalized Regression for Value

Standard Least Squares

Multiple Comparisons for Treatment*Strain*RIP*Time

Tukey HSD All Pairwise Comparisons

All Pairwise Differences

Treatment	Strain	RIP	Time	-Treatment	-Strain	-RIP	-Time	Difference	Std Error	t Ratio	Prob>|t|	Lower 95%	Upper 95%
Lh	Brazil	RIP1	72h	no_wasp	Uganda	RIP2	24h	-8.57653	0.2609050	-32.87	<.0001*	-9.6210	-7.5321
Lh	Brazil	RIP1	72h	no_wasp	Uganda	RIP2	72h	-7.92651	0.2609050	-30.38	<.0001*	-8.9709	-6.8821
Lh	Brazil	RIP1	72h	no_wasp	Uganda	RIP3_5	24h	-0.67483	0.2609050	-2.59	0.7837	-1.7193	0.3696
Lh	Brazil	RIP1	72h	no_wasp	Uganda	RIP3_5	72h	-2.50273	0.2609050	-9.59	<.0001*	-3.5472	-1.4583
Lh	Brazil	RIP2	24h	Lh	Brazil	RIP2	72h	-0.16681	0.2609050	-0.64	1.0000	-1.2112	0.8776
Lh	Brazil	RIP2	24h	Lh	Brazil	RIP3_5	24h	6.98116	0.2609050	26.76	<.0001*	5.9367	8.0256
Lh	Brazil	RIP2	24h	Lh	Brazil	RIP3_5	72h	6.41474	0.2609050	24.59	<.0001*	5.3703	7.4592
Lh	Brazil	RIP2	24h	Lh	Uganda	RIP1	24h	9.01564	0.2609050	34.56	<.0001*	7.9712	10.0601
Lh	Brazil	RIP2	24h	Lh	Uganda	RIP1	72h	8.73513	0.2609050	33.48	<.0001*	7.6907	9.7796
Lh	Brazil	RIP2	24h	Lh	Uganda	RIP2	24h	0.49514	0.2609050	1.90	0.9933	-0.5493	1.5396
Lh	Brazil	RIP2	24h	Lh	Uganda	RIP2	72h	0.10465	0.2609050	0.40	1.0000	-0.9398	1.1491
Lh	Brazil	RIP2	24h	Lh	Uganda	RIP3_5	24h	8.75162	0.2609050	33.54	<.0001*	7.7072	9.7961
Lh	Brazil	RIP2	24h	Lh	Uganda	RIP3_5	72h	7.55279	0.2609050	28.95	<.0001*	6.5084	8.5972
Lh	Brazil	RIP2	24h	no_wasp	Brazil	RIP1	24h	8.33908	0.2609050	31.96	<.0001*	7.2946	9.3835
Lh	Brazil	RIP2	24h	no_wasp	Brazil	RIP1	72h	8.20332	0.2609050	31.44	<.0001*	7.1589	9.2478
Lh	Brazil	RIP2	24h	no_wasp	Brazil	RIP2	24h	-0.09155	0.2609050	-0.35	1.0000	-1.1360	0.9529
Lh	Brazil	RIP2	24h	no_wasp	Brazil	RIP2	72h	-0.41435	0.2609050	-1.59	0.9997	-1.4588	0.6301
Lh	Brazil	RIP2	24h	no_wasp	Brazil	RIP3_5	24h	5.98155	0.2609050	22.93	<.0001*	4.9371	7.0260
Lh	Brazil	RIP2	24h	no_wasp	Brazil	RIP3_5	72h	6.45087	0.2609050	24.72	<.0001*	5.4064	7.4953
Lh	Brazil	RIP2	24h	no_wasp	Uganda	RIP1	24h	8.20763	0.2609050	31.46	<.0001*	7.1632	9.2521
Lh	Brazil	RIP2	24h	no_wasp	Uganda	RIP1	72h	7.72573	0.2609050	29.61	<.0001*	6.6813	8.7702
Lh	Brazil	RIP2	24h	no_wasp	Uganda	RIP2	24h	0.30949	0.2609050	1.19	1.0000	-0.7349	1.3539
Lh	Brazil	RIP2	24h	no_wasp	Uganda	RIP2	72h	0.95950	0.2609050	3.68	0.1208	-0.0849	2.0039
Lh	Brazil	RIP2	24h	no_wasp	Uganda	RIP3_5	24h	8.21118	0.2609050	31.47	<.0001*	7.1667	9.2556
Lh	Brazil	RIP2	24h	no_wasp	Uganda	RIP3_5	72h	6.38328	0.2609050	24.47	<.0001*	5.3388	7.4277
Lh	Brazil	RIP2	72h	Lh	Brazil	RIP3_5	24h	7.14797	0.2609050	27.40	<.0001*	6.1035	8.1924
Lh	Brazil	RIP2	72h	Lh	Brazil	RIP3_5	72h	6.58155	0.2609050	25.23	<.0001*	5.5371	7.6260
Lh	Brazil	RIP2	72h	Lh	Uganda	RIP1	24h	9.18245	0.2609050	35.19	<.0001*	8.1380	10.2269
Lh	Brazil	RIP2	72h	Lh	Uganda	RIP1	72h	8.90193	0.2609050	34.12	<.0001*	7.8575	9.9464
Lh	Brazil	RIP2	72h	Lh	Uganda	RIP2	24h	0.66195	0.2609050	2.54	0.8135	-0.3825	1.7064
Lh	Brazil	RIP2	72h	Lh	Uganda	RIP2	72h	0.27146	0.2609050	1.04	1.0000	-0.7730	1.3159

Generalized Regression for Value
Standard Least Squares
Multiple Comparisons for Treatment*Strain*RIP*Time
Tukey HSD All Pairwise Comparisons
All Pairwise Differences

Treatment	Strain	RIP	Time	-Treatment	-Strain	-RIP	-Time	Difference	Std Error	t Ratio	Prob>|t|	Lower 95%	Upper 95%
Lh	Brazil	RIP2	72h	Lh	Uganda	RIP3_5	24h	8.91843	0.2609050	34.18	<.0001*	7.8740	9.9629
Lh	Brazil	RIP2	72h	Lh	Uganda	RIP3_5	72h	7.71960	0.2609050	29.59	<.0001*	6.6752	8.7640
Lh	Brazil	RIP2	72h	no_wasp	Brazil	RIP1	24h	8.50588	0.2609050	32.60	<.0001*	7.4615	9.5503
Lh	Brazil	RIP2	72h	no_wasp	Brazil	RIP1	72h	8.37013	0.2609050	32.08	<.0001*	7.3257	9.4146
Lh	Brazil	RIP2	72h	no_wasp	Brazil	RIP2	24h	0.07525	0.2609050	0.29	1.0000	-0.9692	1.1197
Lh	Brazil	RIP2	72h	no_wasp	Brazil	RIP2	72h	-0.24754	0.2609050	-0.95	1.0000	-1.2920	0.7969
Lh	Brazil	RIP2	72h	no_wasp	Brazil	RIP3_5	24h	6.14836	0.2609050	23.57	<.0001*	5.1039	7.1928
Lh	Brazil	RIP2	72h	no_wasp	Brazil	RIP3_5	72h	6.61768	0.2609050	25.36	<.0001*	5.5732	7.6621
Lh	Brazil	RIP2	72h	no_wasp	Uganda	RIP1	24h	8.37444	0.2609050	32.10	<.0001*	7.3300	9.4189
Lh	Brazil	RIP2	72h	no_wasp	Uganda	RIP1	72h	7.89254	0.2609050	30.25	<.0001*	6.8481	8.9370
Lh	Brazil	RIP2	72h	no_wasp	Uganda	RIP2	24h	0.47629	0.2609050	1.83	0.9963	-0.5681	1.5207
Lh	Brazil	RIP2	72h	no_wasp	Uganda	RIP2	72h	1.12631	0.2609050	4.32	0.0193*	0.0819	2.1707
Lh	Brazil	RIP2	72h	no_wasp	Uganda	RIP3_5	24h	8.37799	0.2609050	32.11	<.0001*	7.3336	9.4224
Lh	Brazil	RIP2	72h	no_wasp	Uganda	RIP3_5	72h	6.55009	0.2609050	25.11	<.0001*	5.5057	7.5945
Lh	Brazil	RIP3_5	24h	Lh	Brazil	RIP3_5	72h	-0.56642	0.2609050	-2.17	0.9596	-1.6109	0.4780
Lh	Brazil	RIP3_5	24h	Lh	Uganda	RIP1	24h	2.03448	0.2609050	7.80	<.0001*	0.9900	3.0789
Lh	Brazil	RIP3_5	24h	Lh	Uganda	RIP1	72h	1.75396	0.2609050	6.72	<.0001*	0.7095	2.7984
Lh	Brazil	RIP3_5	24h	Lh	Uganda	RIP2	24h	-6.48602	0.2609050	-24.86	<.0001*	-7.5305	-5.4416
Lh	Brazil	RIP3_5	24h	Lh	Uganda	RIP2	72h	-6.87651	0.2609050	-26.36	<.0001*	-7.9209	-5.8321
Lh	Brazil	RIP3_5	24h	Lh	Uganda	RIP3_5	24h	1.77046	0.2609050	6.79	<.0001*	0.7260	2.8149
Lh	Brazil	RIP3_5	24h	Lh	Uganda	RIP3_5	72h	0.57163	0.2609050	2.19	0.9551	-0.4728	1.6161
Lh	Brazil	RIP3_5	24h	no_wasp	Brazil	RIP1	24h	1.35791	0.2609050	5.20	0.0009*	0.3135	2.4023
Lh	Brazil	RIP3_5	24h	no_wasp	Brazil	RIP1	72h	1.22216	0.2609050	4.68	0.0057*	0.1777	2.2666
Lh	Brazil	RIP3_5	24h	no_wasp	Brazil	RIP2	24h	-7.07272	0.2609050	-27.11	<.0001*	-8.1171	-6.0283
Lh	Brazil	RIP3_5	24h	no_wasp	Brazil	RIP2	72h	-7.39551	0.2609050	-28.35	<.0001*	-8.4399	-6.3511
Lh	Brazil	RIP3_5	24h	no_wasp	Brazil	RIP3_5	24h	-0.99961	0.2609050	-3.83	0.0808	-2.0440	0.0448
Lh	Brazil	RIP3_5	24h	no_wasp	Brazil	RIP3_5	72h	-0.53029	0.2609050	-2.03	0.9823	-1.5747	0.5141
Lh	Brazil	RIP3_5	24h	no_wasp	Uganda	RIP1	24h	1.22647	0.2609050	4.70	0.0054*	0.1820	2.2709
Lh	Brazil	RIP3_5	24h	no_wasp	Uganda	RIP1	72h	0.74457	0.2609050	2.85	0.5949	-0.2999	1.7890
Lh	Brazil	RIP3_5	24h	no_wasp	Uganda	RIP2	24h	-6.67168	0.2609050	-25.57	<.0001*	-7.7161	-5.6272
Lh	Brazil	RIP3_5	24h	no_wasp	Uganda	RIP2	72h	-6.02166	0.2609050	-23.08	<.0001*	-7.0661	-4.9772

Generalized Regression for Value

Standard Least Squares

Multiple Comparisons for Treatment*Strain*RIP*Time

Tukey HSD All Pairwise Comparisons

All Pairwise Differences

Treatment	Strain	RIP	Time	-Treatment	-Strain	-RIP	-Time	Difference	Std Error	t Ratio	Prob>|t|	Lower 95%	Upper 95%
Lh	Brazil	RIP3_5	24h	no_wasp	Uganda	RIP3_5	24h	1.23002	0.2609050	4.71	0.0052*	0.1856	2.2744
Lh	Brazil	RIP3_5	24h	no_wasp	Uganda	RIP3_5	72h	-0.59788	0.2609050	-2.29	0.9266	-1.6423	0.4465
Lh	Brazil	RIP3_5	72h	Lh	Uganda	RIP1	24h	2.60090	0.2609050	9.97	<.0001*	1.5565	3.6453
Lh	Brazil	RIP3_5	72h	Lh	Uganda	RIP1	72h	2.32039	0.2609050	8.89	<.0001*	1.2760	3.3648
Lh	Brazil	RIP3_5	72h	Lh	Uganda	RIP2	24h	-5.91960	0.2609050	-22.69	<.0001*	-6.9640	-4.8752
Lh	Brazil	RIP3_5	72h	Lh	Uganda	RIP2	72h	-6.31009	0.2609050	-24.19	<.0001*	-7.3545	-5.2657
Lh	Brazil	RIP3_5	72h	Lh	Uganda	RIP3_5	24h	2.33688	0.2609050	8.96	<.0001*	1.2924	3.3813
Lh	Brazil	RIP3_5	72h	Lh	Uganda	RIP3_5	72h	1.13805	0.2609050	4.36	0.0167*	0.0936	2.1825
Lh	Brazil	RIP3_5	72h	no_wasp	Brazil	RIP1	24h	1.92433	0.2609050	7.38	<.0001*	0.8799	2.9688
Lh	Brazil	RIP3_5	72h	no_wasp	Brazil	RIP1	72h	1.78858	0.2609050	6.86	<.0001*	0.7442	2.8330
Lh	Brazil	RIP3_5	72h	no_wasp	Brazil	RIP2	24h	-6.50629	0.2609050	-24.94	<.0001*	-7.5507	-5.4619
Lh	Brazil	RIP3_5	72h	no_wasp	Brazil	RIP2	72h	-6.82909	0.2609050	-26.17	<.0001*	-7.8735	-5.7847
Lh	Brazil	RIP3_5	72h	no_wasp	Brazil	RIP3_5	24h	-0.43319	0.2609050	-1.66	0.9993	-1.4776	0.6112
Lh	Brazil	RIP3_5	72h	no_wasp	Brazil	RIP3_5	72h	0.03613	0.2609050	0.14	1.0000	-1.0083	1.0806
Lh	Brazil	RIP3_5	72h	no_wasp	Uganda	RIP1	24h	1.79289	0.2609050	6.87	<.0001*	0.7485	2.8373
Lh	Brazil	RIP3_5	72h	no_wasp	Uganda	RIP1	72h	1.31099	0.2609050	5.02	0.0017*	0.2666	2.3554
Lh	Brazil	RIP3_5	72h	no_wasp	Uganda	RIP2	24h	-6.10525	0.2609050	-23.40	<.0001*	-7.1497	-5.0608
Lh	Brazil	RIP3_5	72h	no_wasp	Uganda	RIP2	72h	-5.45524	0.2609050	-20.91	<.0001*	-6.4997	-4.4108
Lh	Brazil	RIP3_5	72h	no_wasp	Uganda	RIP3_5	24h	1.79644	0.2609050	6.89	<.0001*	0.7520	2.8409
Lh	Brazil	RIP3_5	72h	no_wasp	Uganda	RIP3_5	72h	-0.03146	0.2609050	-0.12	1.0000	-1.0759	1.0130
Lh	Uganda	RIP1	24h	Lh	Uganda	RIP1	72h	-0.28052	0.2609050	-1.08	1.0000	-1.3249	0.7639
Lh	Uganda	RIP1	24h	Lh	Uganda	RIP2	24h	-8.52050	0.2609050	-32.66	<.0001*	-9.5649	-7.4761
Lh	Uganda	RIP1	24h	Lh	Uganda	RIP2	72h	-8.91099	0.2609050	-34.15	<.0001*	-9.9554	-7.8666
Lh	Uganda	RIP1	24h	Lh	Uganda	RIP3_5	24h	-0.26402	0.2609050	-1.01	1.0000	-1.3085	0.7804
Lh	Uganda	RIP1	24h	Lh	Uganda	RIP3_5	72h	-1.46285	0.2609050	-5.61	0.0002*	-2.5073	-0.4184
Lh	Uganda	RIP1	24h	no_wasp	Brazil	RIP1	24h	-0.67657	0.2609050	-2.59	0.7795	-1.7210	0.3679
Lh	Uganda	RIP1	24h	no_wasp	Brazil	RIP1	72h	-0.81232	0.2609050	-3.11	0.4039	-1.8568	0.2321
Lh	Uganda	RIP1	24h	no_wasp	Brazil	RIP2	24h	-9.10720	0.2609050	-34.91	<.0001*	-10.1516	-8.0628
Lh	Uganda	RIP1	24h	no_wasp	Brazil	RIP2	72h	-9.42999	0.2609050	-36.14	<.0001*	-10.4744	-8.3856
Lh	Uganda	RIP1	24h	no_wasp	Brazil	RIP3_5	24h	-3.03409	0.2609050	-11.63	<.0001*	-4.0785	-1.9897
Lh	Uganda	RIP1	24h	no_wasp	Brazil	RIP3_5	72h	-2.56477	0.2609050	-9.83	<.0001*	-3.6092	-1.5203

Generalized Regression for Value

Standard Least Squares

Multiple Comparisons for Treatment*Strain*RIP*Time

Tukey HSD All Pairwise Comparisons

All Pairwise Differences

Treatment	Strain	RIP	Time	-Treatment	-Strain	-RIP	-Time	Difference	Std Error	t Ratio	Prob>|t|	Lower 95%	Upper 95%
Lh	Uganda	RIP1	24h	no_wasp	Uganda	RIP1	24h	-0.80801	0.2609050	-3.10	0.4154	-1.8524	0.2364
Lh	Uganda	RIP1	24h	no_wasp	Uganda	RIP1	72h	-1.28991	0.2609050	-4.94	0.0023*	-2.3343	-0.2455
Lh	Uganda	RIP1	24h	no_wasp	Uganda	RIP2	24h	-8.70616	0.2609050	-33.37	<.0001*	-9.7506	-7.6617
Lh	Uganda	RIP1	24h	no_wasp	Uganda	RIP2	72h	-8.05614	0.2609050	-30.88	<.0001*	-9.1006	-7.0117
Lh	Uganda	RIP1	24h	no_wasp	Uganda	RIP3_5	24h	-0.80446	0.2609050	-3.08	0.4249	-1.8489	0.2400
Lh	Uganda	RIP1	24h	no_wasp	Uganda	RIP3_5	72h	-2.63236	0.2609050	-10.09	<.0001*	-3.6768	-1.5879
Lh	Uganda	RIP1	72h	Lh	Uganda	RIP2	24h	-8.23999	0.2609050	-31.58	<.0001*	-9.2844	-7.1956
Lh	Uganda	RIP1	72h	Lh	Uganda	RIP2	72h	-8.63048	0.2609050	-33.08	<.0001*	-9.6749	-7.5860
Lh	Uganda	RIP1	72h	Lh	Uganda	RIP3_5	24h	0.01649	0.2609050	0.06	1.0000	-1.0279	1.0609
Lh	Uganda	RIP1	72h	Lh	Uganda	RIP3_5	72h	-1.18234	0.2609050	-4.53	0.0096*	-2.2268	-0.1379
Lh	Uganda	RIP1	72h	no_wasp	Brazil	RIP1	24h	-0.39605	0.2609050	-1.52	0.9999	-1.4405	0.6484
Lh	Uganda	RIP1	72h	no_wasp	Brazil	RIP1	72h	-0.53180	0.2609050	-2.04	0.9817	-1.5762	0.5126
Lh	Uganda	RIP1	72h	no_wasp	Brazil	RIP2	24h	-8.82668	0.2609050	-33.83	<.0001*	-9.8711	-7.7823
Lh	Uganda	RIP1	72h	no_wasp	Brazil	RIP2	72h	-9.14948	0.2609050	-35.07	<.0001*	-10.1939	-8.1050
Lh	Uganda	RIP1	72h	no_wasp	Brazil	RIP3_5	24h	-2.75358	0.2609050	-10.55	<.0001*	-3.7980	-1.7091
Lh	Uganda	RIP1	72h	no_wasp	Brazil	RIP3_5	72h	-2.28426	0.2609050	-8.76	<.0001*	-3.3287	-1.2398
Lh	Uganda	RIP1	72h	no_wasp	Uganda	RIP1	24h	-0.52750	0.2609050	-2.02	0.9836	-1.5719	0.5169
Lh	Uganda	RIP1	72h	no_wasp	Uganda	RIP1	72h	-1.00940	0.2609050	-3.87	0.0730	-2.0538	0.0350
Lh	Uganda	RIP1	72h	no_wasp	Uganda	RIP2	24h	-8.42564	0.2609050	-32.29	<.0001*	-9.4701	-7.3812
Lh	Uganda	RIP1	72h	no_wasp	Uganda	RIP2	72h	-7.77563	0.2609050	-29.80	<.0001*	-8.8201	-6.7312
Lh	Uganda	RIP1	72h	no_wasp	Uganda	RIP3_5	24h	-0.52395	0.2609050	-2.01	0.9850	-1.5684	0.5205
Lh	Uganda	RIP1	72h	no_wasp	Uganda	RIP3_5	72h	-2.35185	0.2609050	-9.01	<.0001*	-3.3963	-1.3074
Lh	Uganda	RIP2	24h	Lh	Uganda	RIP2	72h	-0.39049	0.2609050	-1.50	0.9999	-1.4349	0.6539
Lh	Uganda	RIP2	24h	Lh	Uganda	RIP3_5	24h	8.25648	0.2609050	31.65	<.0001*	7.2120	9.3009
Lh	Uganda	RIP2	24h	Lh	Uganda	RIP3_5	72h	7.05765	0.2609050	27.05	<.0001*	6.0132	8.1021
Lh	Uganda	RIP2	24h	no_wasp	Brazil	RIP1	24h	7.84394	0.2609050	30.06	<.0001*	6.7995	8.8884
Lh	Uganda	RIP2	24h	no_wasp	Brazil	RIP1	72h	7.70818	0.2609050	29.54	<.0001*	6.6638	8.7526
Lh	Uganda	RIP2	24h	no_wasp	Brazil	RIP2	24h	-0.58669	0.2609050	-2.25	0.9400	-1.6311	0.4577
Lh	Uganda	RIP2	24h	no_wasp	Brazil	RIP2	72h	-0.90949	0.2609050	-3.49	0.1915	-1.9539	0.1349
Lh	Uganda	RIP2	24h	no_wasp	Brazil	RIP3_5	24h	5.48641	0.2609050	21.03	<.0001*	4.4420	6.5308
Lh	Uganda	RIP2	24h	no_wasp	Brazil	RIP3_5	72h	5.95573	0.2609050	22.83	<.0001*	4.9113	7.0002

Generalized Regression for Value

Standard Least Squares

Multiple Comparisons for Treatment*Strain*RIP*Time

Tukey HSD All Pairwise Comparisons

All Pairwise Differences

Treatment	Strain	RIP	Time	-Treatment	-Strain	-RIP	-Time	Difference	Std Error	t Ratio	Prob>|t|	Lower 95%	Upper 95%
Lh	Uganda	RIP2	24h	no_wasp	Uganda	RIP1	24h	7.71249	0.2609050	29.56	<.0001*	6.6681	8.7569
Lh	Uganda	RIP2	24h	no_wasp	Uganda	RIP1	72h	7.23059	0.2609050	27.71	<.0001*	6.1862	8.2750
Lh	Uganda	RIP2	24h	no_wasp	Uganda	RIP2	24h	-0.18565	0.2609050	-0.71	1.0000	-1.2301	0.8588
Lh	Uganda	RIP2	24h	no_wasp	Uganda	RIP2	72h	0.46436	0.2609050	1.78	0.9976	-0.5801	1.5088
Lh	Uganda	RIP2	24h	no_wasp	Uganda	RIP3_5	24h	7.71604	0.2609050	29.57	<.0001*	6.6716	8.7605
Lh	Uganda	RIP2	24h	no_wasp	Uganda	RIP3_5	72h	5.88814	0.2609050	22.57	<.0001*	4.8437	6.9326
Lh	Uganda	RIP2	72h	Lh	Uganda	RIP3_5	24h	8.64697	0.2609050	33.14	<.0001*	7.6025	9.6914
Lh	Uganda	RIP2	72h	Lh	Uganda	RIP3_5	72h	7.44814	0.2609050	28.55	<.0001*	6.4037	8.4926
Lh	Uganda	RIP2	72h	no_wasp	Brazil	RIP1	24h	8.23443	0.2609050	31.56	<.0001*	7.1900	9.2789
Lh	Uganda	RIP2	72h	no_wasp	Brazil	RIP1	72h	8.09867	0.2609050	31.04	<.0001*	7.0542	9.1431
Lh	Uganda	RIP2	72h	no_wasp	Brazil	RIP2	24h	-0.19620	0.2609050	-0.75	1.0000	-1.2406	0.8482
Lh	Uganda	RIP2	72h	no_wasp	Brazil	RIP2	72h	-0.51900	0.2609050	-1.99	0.9868	-1.5634	0.5254
Lh	Uganda	RIP2	72h	no_wasp	Brazil	RIP3_5	24h	5.87690	0.2609050	22.53	<.0001*	4.8325	6.9213
Lh	Uganda	RIP2	72h	no_wasp	Brazil	RIP3_5	72h	6.34622	0.2609050	24.32	<.0001*	5.3018	7.3907
Lh	Uganda	RIP2	72h	no_wasp	Uganda	RIP1	24h	8.10298	0.2609050	31.06	<.0001*	7.0586	9.1474
Lh	Uganda	RIP2	72h	no_wasp	Uganda	RIP1	72h	7.62108	0.2609050	29.21	<.0001*	6.5766	8.6655
Lh	Uganda	RIP2	72h	no_wasp	Uganda	RIP2	24h	0.20484	0.2609050	0.79	1.0000	-0.8396	1.2493
Lh	Uganda	RIP2	72h	no_wasp	Uganda	RIP2	72h	0.85485	0.2609050	3.28	0.2990	-0.1896	1.8993
Lh	Uganda	RIP2	72h	no_wasp	Uganda	RIP3_5	24h	8.10653	0.2609050	31.07	<.0001*	7.0621	9.1510
Lh	Uganda	RIP2	72h	no_wasp	Uganda	RIP3_5	72h	6.27863	0.2609050	24.06	<.0001*	5.2342	7.3231
Lh	Uganda	RIP3_5	24h	Lh	Uganda	RIP3_5	72h	-1.19883	0.2609050	-4.59	0.0078*	-2.2433	-0.1544
Lh	Uganda	RIP3_5	24h	no_wasp	Brazil	RIP1	24h	-0.41255	0.2609050	-1.58	0.9997	-1.4570	0.6319
Lh	Uganda	RIP3_5	24h	no_wasp	Brazil	RIP1	72h	-0.54830	0.2609050	-2.10	0.9728	-1.5927	0.4961
Lh	Uganda	RIP3_5	24h	no_wasp	Brazil	RIP2	24h	-8.84317	0.2609050	-33.89	<.0001*	-9.8876	-7.7987
Lh	Uganda	RIP3_5	24h	no_wasp	Brazil	RIP2	72h	-9.16597	0.2609050	-35.13	<.0001*	-10.2104	-8.1215
Lh	Uganda	RIP3_5	24h	no_wasp	Brazil	RIP3_5	24h	-2.77007	0.2609050	-10.62	<.0001*	-3.8145	-1.7256
Lh	Uganda	RIP3_5	24h	no_wasp	Brazil	RIP3_5	72h	-2.30075	0.2609050	-8.82	<.0001*	-3.3452	-1.2563
Lh	Uganda	RIP3_5	24h	no_wasp	Uganda	RIP1	24h	-0.54399	0.2609050	-2.09	0.9754	-1.5884	0.5004
Lh	Uganda	RIP3_5	24h	no_wasp	Uganda	RIP1	72h	-1.02589	0.2609050	-3.93	0.0612	-2.0703	0.0185
Lh	Uganda	RIP3_5	24h	no_wasp	Uganda	RIP2	24h	-8.44213	0.2609050	-32.36	<.0001*	-9.4866	-7.3977
Lh	Uganda	RIP3_5	24h	no_wasp	Uganda	RIP2	72h	-7.79212	0.2609050	-29.87	<.0001*	-8.8365	-6.7477

Generalized Regression for Value

Standard Least Squares

Multiple Comparisons for Treatment*Strain*RIP*Time

Tukey HSD All Pairwise Comparisons

All Pairwise Differences

Treatment	Strain	RIP	Time	-Treatment	-Strain	-RIP	-Time	Difference	Std Error	t Ratio	Prob>|t|	Lower 95%	Upper 95%
Lh	Uganda	RIP3_5	24h	no_wasp	Uganda	RIP3_5	24h	-0.54044	0.2609050	-2.07	0.9774	-1.5849	0.5040
Lh	Uganda	RIP3_5	24h	no_wasp	Uganda	RIP3_5	72h	-2.36834	0.2609050	-9.08	<.0001*	-3.4128	-1.3239
Lh	Uganda	RIP3_5	72h	no_wasp	Brazil	RIP1	24h	0.78628	0.2609050	3.01	0.4752	-0.2581	1.8307
Lh	Uganda	RIP3_5	72h	no_wasp	Brazil	RIP1	72h	0.65053	0.2609050	2.49	0.8382	-0.3939	1.6950
Lh	Uganda	RIP3_5	72h	no_wasp	Brazil	RIP2	24h	-7.64435	0.2609050	-29.30	<.0001*	-8.6888	-6.5999
Lh	Uganda	RIP3_5	72h	no_wasp	Brazil	RIP2	72h	-7.96714	0.2609050	-30.54	<.0001*	-9.0116	-6.9227
Lh	Uganda	RIP3_5	72h	no_wasp	Brazil	RIP3_5	24h	-1.57124	0.2609050	-6.02	<.0001*	-2.6157	-0.5268
Lh	Uganda	RIP3_5	72h	no_wasp	Brazil	RIP3_5	72h	-1.10192	0.2609050	-4.22	0.0259*	-2.1464	-0.0575
Lh	Uganda	RIP3_5	72h	no_wasp	Uganda	RIP1	24h	0.65484	0.2609050	2.51	0.8291	-0.3896	1.6993
Lh	Uganda	RIP3_5	72h	no_wasp	Uganda	RIP1	72h	0.17294	0.2609050	0.66	1.0000	-0.8715	1.2174
Lh	Uganda	RIP3_5	72h	no_wasp	Uganda	RIP2	24h	-7.24331	0.2609050	-27.76	<.0001*	-8.2877	-6.1989
Lh	Uganda	RIP3_5	72h	no_wasp	Uganda	RIP2	72h	-6.59329	0.2609050	-25.27	<.0001*	-7.6377	-5.5489
Lh	Uganda	RIP3_5	72h	no_wasp	Uganda	RIP3_5	24h	0.65839	0.2609050	2.52	0.8214	-0.3860	1.7028
Lh	Uganda	RIP3_5	72h	no_wasp	Uganda	RIP3_5	72h	-1.16951	0.2609050	-4.48	0.0113*	-2.2139	-0.1251
no_wasp	Brazil	RIP1	24h	no_wasp	Brazil	RIP1	72h	-0.13575	0.2609050	-0.52	1.0000	-1.1802	0.9087
no_wasp	Brazil	RIP1	24h	no_wasp	Brazil	RIP2	24h	-8.43063	0.2609050	-32.31	<.0001*	-9.4751	-7.3862
no_wasp	Brazil	RIP1	24h	no_wasp	Brazil	RIP2	72h	-8.75342	0.2609050	-33.55	<.0001*	-9.7979	-7.7090
no_wasp	Brazil	RIP1	24h	no_wasp	Brazil	RIP3_5	24h	-2.35753	0.2609050	-9.04	<.0001*	-3.4020	-1.3131
no_wasp	Brazil	RIP1	24h	no_wasp	Brazil	RIP3_5	72h	-1.88821	0.2609050	-7.24	<.0001*	-2.9326	-0.8438
no_wasp	Brazil	RIP1	24h	no_wasp	Uganda	RIP1	24h	-0.13144	0.2609050	-0.50	1.0000	-1.1759	0.9130
no_wasp	Brazil	RIP1	24h	no_wasp	Uganda	RIP1	72h	-0.61335	0.2609050	-2.35	0.9050	-1.6578	0.4311
no_wasp	Brazil	RIP1	24h	no_wasp	Uganda	RIP2	24h	-8.02959	0.2609050	-30.78	<.0001*	-9.0740	-6.9852
no_wasp	Brazil	RIP1	24h	no_wasp	Uganda	RIP2	72h	-7.37957	0.2609050	-28.28	<.0001*	-8.4240	-6.3351
no_wasp	Brazil	RIP1	24h	no_wasp	Uganda	RIP3_5	24h	-0.12790	0.2609050	-0.49	1.0000	-1.1723	0.9165
no_wasp	Brazil	RIP1	24h	no_wasp	Uganda	RIP3_5	72h	-1.95580	0.2609050	-7.50	<.0001*	-3.0002	-0.9114
no_wasp	Brazil	RIP1	72h	no_wasp	Brazil	RIP2	24h	-8.29488	0.2609050	-31.79	<.0001*	-9.3393	-7.2504
no_wasp	Brazil	RIP1	72h	no_wasp	Brazil	RIP2	72h	-8.61767	0.2609050	-33.03	<.0001*	-9.6621	-7.5732
no_wasp	Brazil	RIP1	72h	no_wasp	Brazil	RIP3_5	24h	-2.22177	0.2609050	-8.52	<.0001*	-3.2662	-1.1773
no_wasp	Brazil	RIP1	72h	no_wasp	Brazil	RIP3_5	72h	-1.75245	0.2609050	-6.72	<.0001*	-2.7969	-0.7080
no_wasp	Brazil	RIP1	72h	no_wasp	Uganda	RIP1	24h	0.00431	0.2609050	0.02	1.0000	-1.0401	1.0487
no_wasp	Brazil	RIP1	72h	no_wasp	Uganda	RIP1	72h	-0.47759	0.2609050	-1.83	0.9962	-1.5220	0.5668

Generalized Regression for Value

Standard Least Squares

Multiple Comparisons for Treatment*Strain*RIP*Time

Tukey HSD All Pairwise Comparisons

All Pairwise Differences

Treatment	Strain	RIP	Time	-Treatment	-Strain	-RIP	-Time	Difference	Std Error	t Ratio	Prob>|t|	Lower 95%	Upper 95%
no_wasp	Brazil	RIP1	72h	no_wasp	Uganda	RIP2	24h	-7.89384	0.2609050	-30.26	<.0001*	-8.9383	-6.8494
no_wasp	Brazil	RIP1	72h	no_wasp	Uganda	RIP2	72h	-7.24382	0.2609050	-27.76	<.0001*	-8.2883	-6.1994
no_wasp	Brazil	RIP1	72h	no_wasp	Uganda	RIP3_5	24h	0.00786	0.2609050	0.03	1.0000	-1.0366	1.0523
no_wasp	Brazil	RIP1	72h	no_wasp	Uganda	RIP3_5	72h	-1.82004	0.2609050	-6.98	<.0001*	-2.8645	-0.7756
no_wasp	Brazil	RIP2	24h	no_wasp	Brazil	RIP2	72h	-0.32280	0.2609050	-1.24	1.0000	-1.3672	0.7216
no_wasp	Brazil	RIP2	24h	no_wasp	Brazil	RIP3_5	24h	6.07310	0.2609050	23.28	<.0001*	5.0287	7.1175
no_wasp	Brazil	RIP2	24h	no_wasp	Brazil	RIP3_5	72h	6.54242	0.2609050	25.08	<.0001*	5.4980	7.5869
no_wasp	Brazil	RIP2	24h	no_wasp	Uganda	RIP1	24h	8.29919	0.2609050	31.81	<.0001*	7.2548	9.3436
no_wasp	Brazil	RIP2	24h	no_wasp	Uganda	RIP1	72h	7.81728	0.2609050	29.96	<.0001*	6.7729	8.8617
no_wasp	Brazil	RIP2	24h	no_wasp	Uganda	RIP2	24h	0.40104	0.2609050	1.54	0.9998	-0.6434	1.4455
no_wasp	Brazil	RIP2	24h	no_wasp	Uganda	RIP2	72h	1.05106	0.2609050	4.03	0.0464*	0.0066	2.0955
no_wasp	Brazil	RIP2	24h	no_wasp	Uganda	RIP3_5	24h	8.30273	0.2609050	31.82	<.0001*	7.2583	9.3472
no_wasp	Brazil	RIP2	24h	no_wasp	Uganda	RIP3_5	72h	6.47483	0.2609050	24.82	<.0001*	5.4304	7.5193
no_wasp	Brazil	RIP2	72h	no_wasp	Brazil	RIP3_5	24h	6.39590	0.2609050	24.51	<.0001*	5.3515	7.4403
no_wasp	Brazil	RIP2	72h	no_wasp	Brazil	RIP3_5	72h	6.86522	0.2609050	26.31	<.0001*	5.8208	7.9096
no_wasp	Brazil	RIP2	72h	no_wasp	Uganda	RIP1	24h	8.62198	0.2609050	33.05	<.0001*	7.5776	9.6664
no_wasp	Brazil	RIP2	72h	no_wasp	Uganda	RIP1	72h	8.14008	0.2609050	31.20	<.0001*	7.0956	9.1845
no_wasp	Brazil	RIP2	72h	no_wasp	Uganda	RIP2	24h	0.72384	0.2609050	2.77	0.6542	-0.3206	1.7683
no_wasp	Brazil	RIP2	72h	no_wasp	Uganda	RIP2	72h	1.37385	0.2609050	5.27	0.0007*	0.3294	2.4183
no_wasp	Brazil	RIP2	72h	no_wasp	Uganda	RIP3_5	24h	8.62553	0.2609050	33.06	<.0001*	7.5811	9.6700
no_wasp	Brazil	RIP2	72h	no_wasp	Uganda	RIP3_5	72h	6.79763	0.2609050	26.05	<.0001*	5.7532	7.8421
no_wasp	Brazil	RIP3_5	24h	no_wasp	Brazil	RIP3_5	72h	0.46932	0.2609050	1.80	0.9971	-0.5751	1.5138
no_wasp	Brazil	RIP3_5	24h	no_wasp	Uganda	RIP1	24h	2.22608	0.2609050	8.53	<.0001*	1.1817	3.2705
no_wasp	Brazil	RIP3_5	24h	no_wasp	Uganda	RIP1	72h	1.74418	0.2609050	6.69	<.0001*	0.6997	2.7886
no_wasp	Brazil	RIP3_5	24h	no_wasp	Uganda	RIP2	24h	-5.67206	0.2609050	-21.74	<.0001*	-6.7165	-4.6276
no_wasp	Brazil	RIP3_5	24h	no_wasp	Uganda	RIP2	72h	-5.02205	0.2609050	-19.25	<.0001*	-6.0665	-3.9776
no_wasp	Brazil	RIP3_5	24h	no_wasp	Uganda	RIP3_5	24h	2.22963	0.2609050	8.55	<.0001*	1.1852	3.2741
no_wasp	Brazil	RIP3_5	24h	no_wasp	Uganda	RIP3_5	72h	0.40173	0.2609050	1.54	0.9998	-0.6427	1.4462
no_wasp	Brazil	RIP3_5	72h	no_wasp	Uganda	RIP1	24h	1.75676	0.2609050	6.73	<.0001*	0.7123	2.8012
no_wasp	Brazil	RIP3_5	72h	no_wasp	Uganda	RIP1	72h	1.27486	0.2609050	4.89	0.0028*	0.2304	2.3193
no_wasp	Brazil	RIP3_5	72h	no_wasp	Uganda	RIP2	24h	-6.14138	0.2609050	-23.54	<.0001*	-7.1858	-5.0970

Generalized Regression for Value

Standard Least Squares

Multiple Comparisons for Treatment*Strain*RIP*Time

Tukey HSD All Pairwise Comparisons

All Pairwise Differences

Treatment	Strain	RIP	Time	-Treatment	-Strain	-RIP	-Time	Difference	Std Error	t Ratio	Prob>|t|	Lower 95%	Upper 95%
no_wasp	Brazil	RIP3_5	72h	no_wasp	Uganda	RIP2	72h	-5.49137	0.2609050	-21.05	<.0001*	-6.5358	-4.4469
no_wasp	Brazil	RIP3_5	72h	no_wasp	Uganda	RIP3_5	24h	1.76031	0.2609050	6.75	<.0001*	0.7159	2.8047
no_wasp	Brazil	RIP3_5	72h	no_wasp	Uganda	RIP3_5	72h	-0.06759	0.2609050	-0.26	1.0000	-1.1120	0.9768
no_wasp	Uganda	RIP1	24h	no_wasp	Uganda	RIP1	72h	-0.48190	0.2609050	-1.85	0.9956	-1.5263	0.5625
no_wasp	Uganda	RIP1	24h	no_wasp	Uganda	RIP2	24h	-7.89815	0.2609050	-30.27	<.0001*	-8.9426	-6.8537
no_wasp	Uganda	RIP1	24h	no_wasp	Uganda	RIP2	72h	-7.24813	0.2609050	-27.78	<.0001*	-8.2926	-6.2037
no_wasp	Uganda	RIP1	24h	no_wasp	Uganda	RIP3_5	24h	0.00355	0.2609050	0.01	1.0000	-1.0409	1.0480
no_wasp	Uganda	RIP1	24h	no_wasp	Uganda	RIP3_5	72h	-1.82435	0.2609050	-6.99	<.0001*	-2.8688	-0.7799
no_wasp	Uganda	RIP1	72h	no_wasp	Uganda	RIP2	24h	-7.41624	0.2609050	-28.43	<.0001*	-8.4607	-6.3718
no_wasp	Uganda	RIP1	72h	no_wasp	Uganda	RIP2	72h	-6.76623	0.2609050	-25.93	<.0001*	-7.8107	-5.7218
no_wasp	Uganda	RIP1	72h	no_wasp	Uganda	RIP3_5	24h	0.48545	0.2609050	1.86	0.9951	-0.5590	1.5299
no_wasp	Uganda	RIP1	72h	no_wasp	Uganda	RIP3_5	72h	-1.34245	0.2609050	-5.15	0.0011*	-2.3869	-0.2980
no_wasp	Uganda	RIP2	24h	no_wasp	Uganda	RIP2	72h	0.65002	0.2609050	2.49	0.8392	-0.3944	1.6944
no_wasp	Uganda	RIP2	24h	no_wasp	Uganda	RIP3_5	24h	7.90169	0.2609050	30.29	<.0001*	6.8573	8.9461
no_wasp	Uganda	RIP2	24h	no_wasp	Uganda	RIP3_5	72h	6.07379	0.2609050	23.28	<.0001*	5.0294	7.1182
no_wasp	Uganda	RIP2	72h	no_wasp	Uganda	RIP3_5	24h	7.25168	0.2609050	27.79	<.0001*	6.2072	8.2961
no_wasp	Uganda	RIP2	72h	no_wasp	Uganda	RIP3_5	72h	5.42378	0.2609050	20.79	<.0001*	4.3793	6.4682
no_wasp	Uganda	RIP3_5	24h	no_wasp	Uganda	RIP3_5	72h	-1.82790	0.2609050	-7.01	<.0001*	-2.8723	-0.7835

All Pairwise Differences Connecting Letters

Generalized Regression for Value

Standard Least Squares

Multiple Comparisons for Treatment*Strain*RIP*Time

Tukey HSD All Pairwise Comparisons

All Pairwise Differences Connecting Letters

Treatment	Strain	RIP	Time												Least Squares Mean
no_wasp	Brazil	RIP2	72h	A											6.291143
Lh	Brazil	RIP2	72h	A											6.043600
no_wasp	Brazil	RIP2	24h	A											5.968347
Gh	Brazil	RIP2	72h	A	B										5.917466
Lh	Brazil	RIP2	24h	A	B										5.876795
Gh	Uganda	RIP2	72h	A	B										5.872945
Gh	Brazil	RIP2	24h	A	B										5.782242
Lh	Uganda	RIP2	72h	A	B										5.772145
no_wasp	Uganda	RIP2	24h	A	B										5.567307
Gh	Uganda	RIP2	24h	A	B										5.422300
Lh	Uganda	RIP2	24h	A	B										5.381654
no_wasp	Uganda	RIP2	72h		B										4.917291
Gh	Brazil	RIP3_5	72h			C									0.103052
no_wasp	Brazil	RIP3_5	24h			C	D								-0.104756
no_wasp	Uganda	RIP3_5	72h			C	D								-0.506486
Lh	Brazil	RIP3_5	72h			C	D								-0.537947
no_wasp	Brazil	RIP3_5	72h			C	D								-0.574076
Lh	Brazil	RIP3_5	24h				D	E							-1.104370
Lh	Uganda	RIP3_5	72h					E	F						-1.675999
Gh	Brazil	RIP3_5	24h					E	F	G					-1.771293
no_wasp	Uganda	RIP1	72h					E	F	G	H				-1.848935
no_wasp	Brazil	RIP1	72h						F	G	H	I			-2.326530
no_wasp	Uganda	RIP1	24h						F	G	H	I			-2.330839
no_wasp	Uganda	RIP3_5	24h						F	G	H	I			-2.334385
no_wasp	Brazil	RIP1	24h						F	G	H	I	J		-2.462281
Gh	Uganda	RIP3_5	72h						F	G	H	I	J	K	-2.660070
Gh	Brazil	RIP1	72h							G	H	I	J	K	-2.751779
Lh	Uganda	RIP1	72h								H	I	J	K	-2.858335
Lh	Uganda	RIP3_5	24h								H	I	J	K	-2.874826
Lh	Brazil	RIP1	72h									I	J	K	-3.009218

Generalized Regression for Value

Standard Least Squares

Multiple Comparisons for Treatment*Strain*RIP*Time

Tukey HSD All Pairwise Comparisons

All Pairwise Differences Connecting Letters

Treatment	Strain	RIP	Time				Least Squares Mean
Gh	Uganda	RIP1	72h	I	J	K	-3.133749
Lh	Uganda	RIP1	24h	I	J	K	-3.138850
Gh	Uganda	RIP3_5	24h		J	K	-3.434118
Gh	Uganda	RIP1	24h		J	K	-3.438974
Gh	Brazil	RIP1	24h		J	K	-3.443138
Lh	Brazil	RIP1	24h			K	-3.508949

Levels not connected by same letter are significantly different.

All Pairwise Comparisons Scatterplot

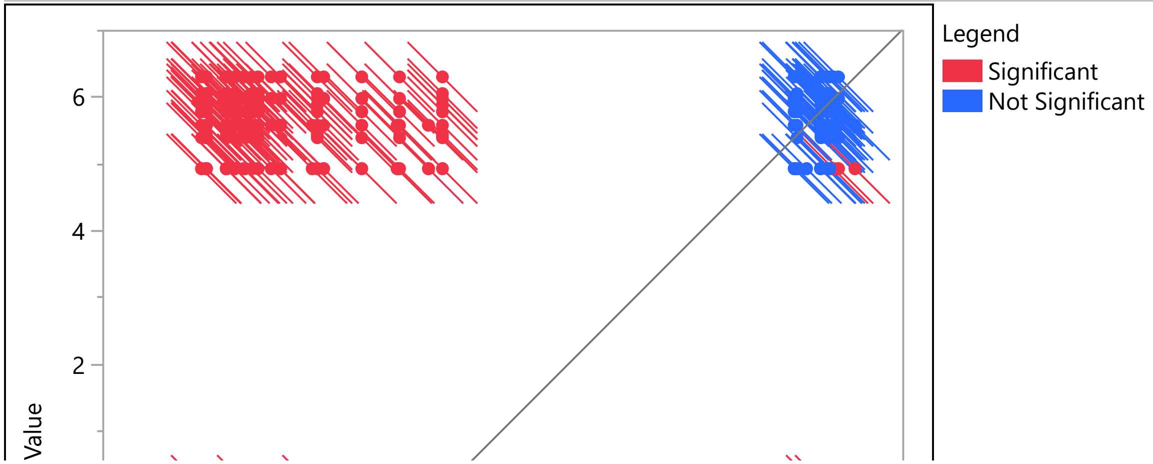

Generalized Regression for Value

Standard Least Squares

Multiple Comparisons for Treatment*Strain*RIP*Time

Tukey HSD All Pairwise Comparisons

All Pairwise Comparisons Scatterplot

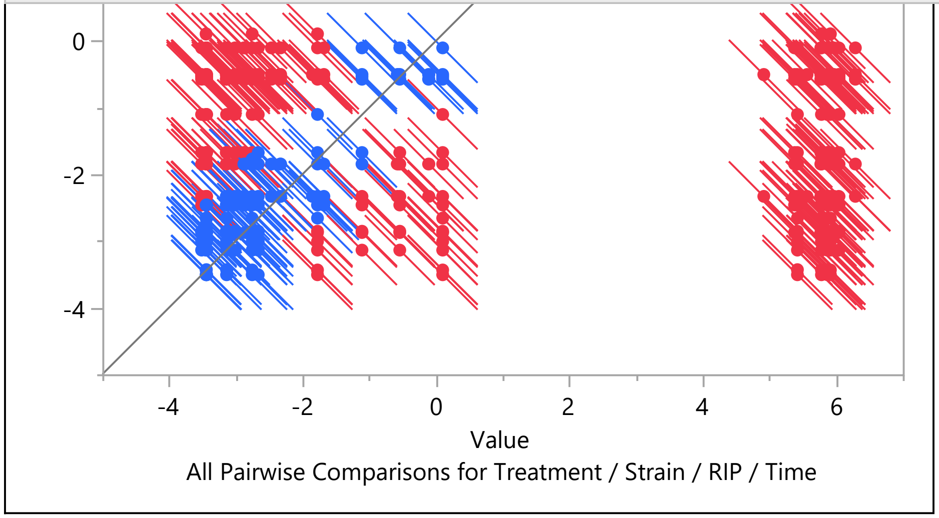

Supplement: Supplemental Information 9 — Full results of the statistical analysis performed with JMP Pro v.15 software. The analyzed data are qPCR values of gene expression of Spiroplasma RIP genes. [file peerj-09-11020-s009.pdf]
