# Supplementary material for: Differential gene expression in a tripartite interaction: Drosophila, Spiroplasma and parasitic wasps
Source: PeerJ. 2021 Mar 4;9:e11020. doi: 10.7717/peerj.11020 (PMC7937342; doi:10.7717/peerj.11020)
Supplement: Supplemental Information 14 [file peerj-09-11020-s014.pdf]

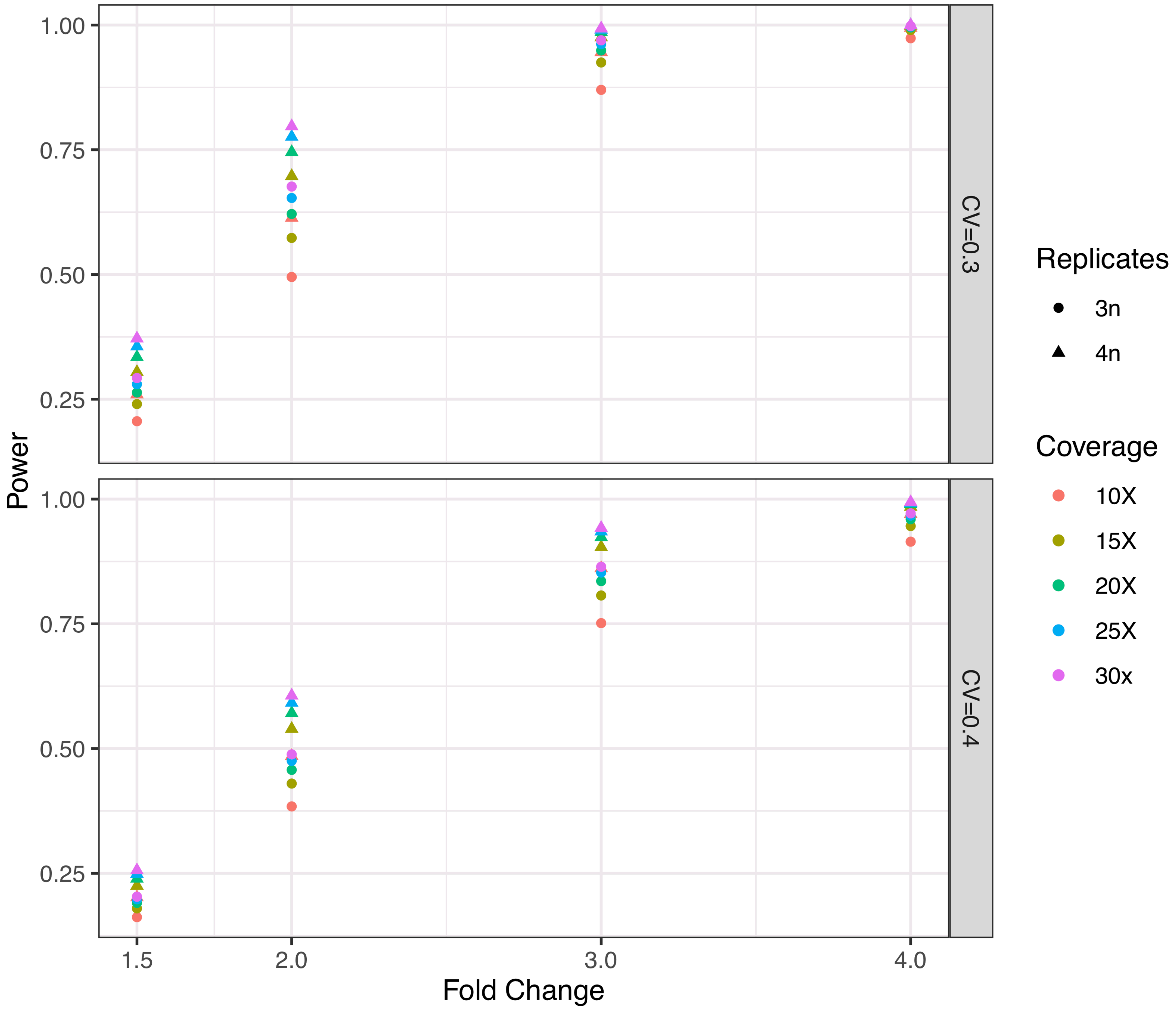

Figure S1. Power Analyses. Estimation of power for alfa = 0.05, at five different effect values (Fold change: 1.5, 2, 3 and 4); number of replicates (n = 3 and 4), coverage values (X = 10, 15, 20 and 25), and coefficient of variation (CV = 0.3 and 0.4).

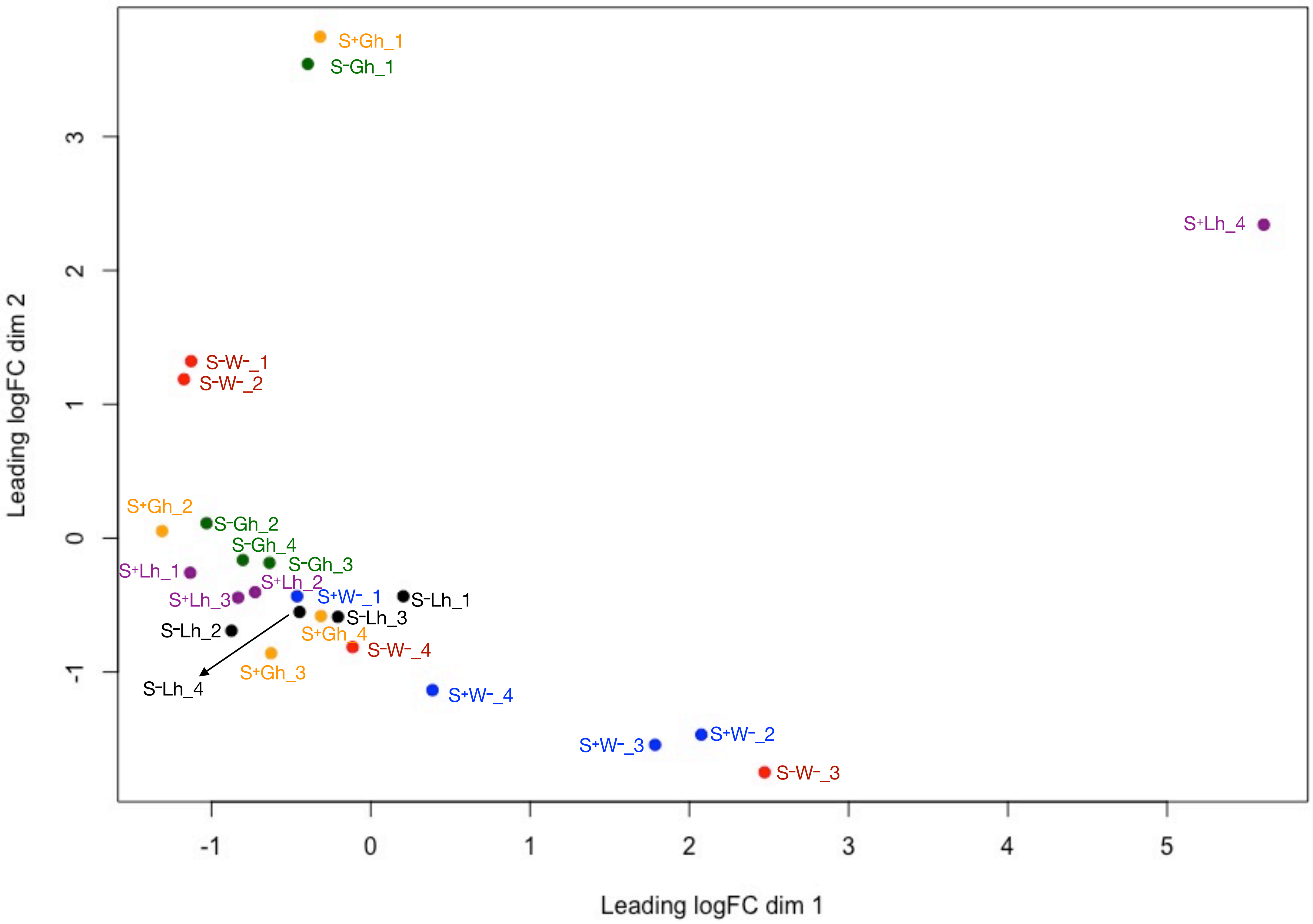

Figure S2. Multidimensional Scaling Plot of *D. melanogaster* gene expression for all treatments generated at Time point 1 (24h post-wasp attack, PWA). Replicates are separated by colors, S^-W^- (red), S^+W^- (blue), S^-Lh (black), S^+Lh (purple), S^-Gh (green) and S^+Gh (yellow). Lh= *L. heterotoma*, Gh= *Ganaspis sp*. S^-= *Spiroplasma*-free, S^+= *Spiroplasma* infected and W^-= Wasp-free.

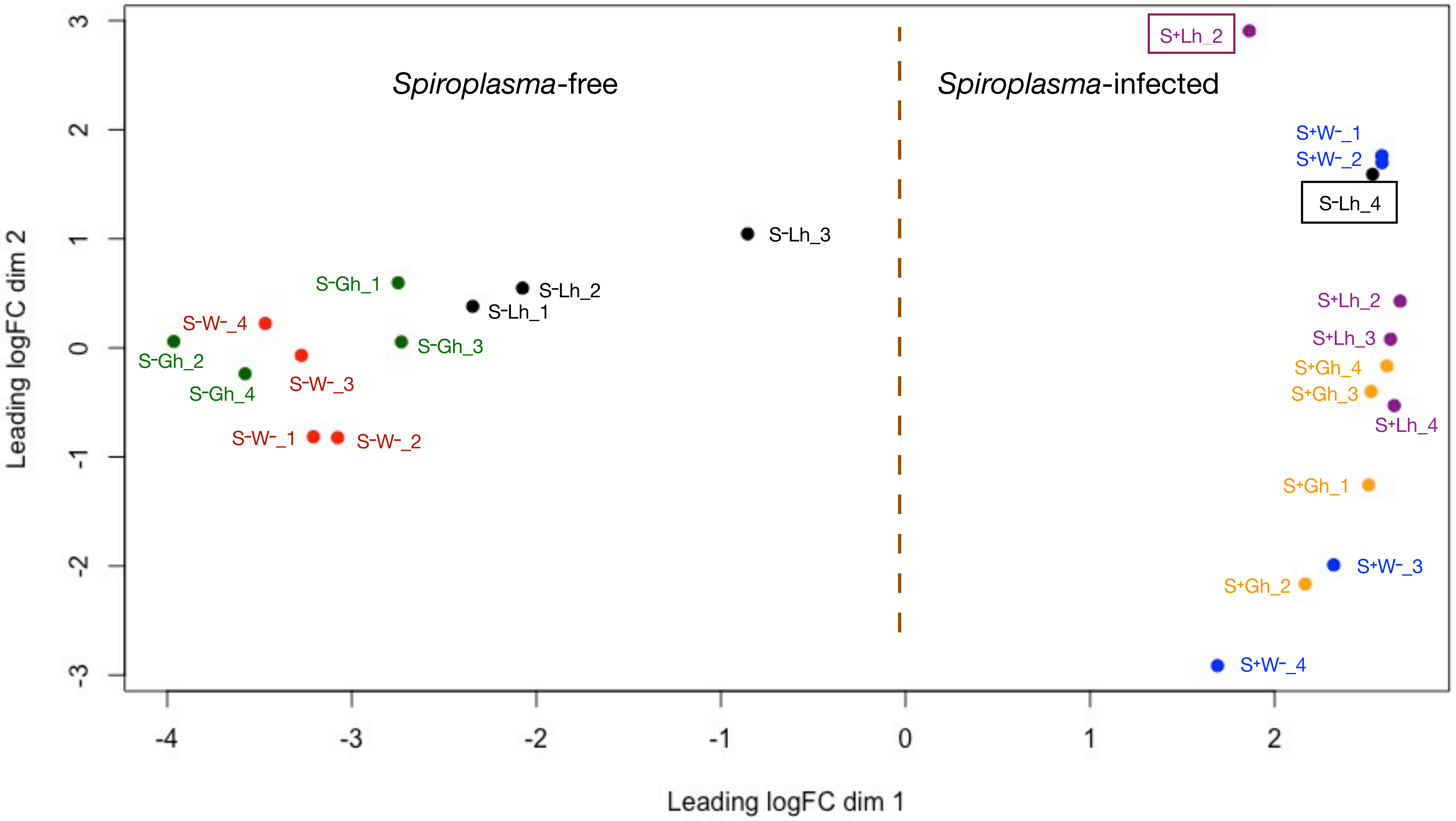

Figure S3. Multidimensional Scaling Plot of *D. melanogaster* gene expression for all treatments generated at Time point 2 (72h post-wasp attack, PWA). Replicates are separated by colors, S^-W^- (red), S^+W^- (blue), S^-Lh (black), S^+Lh (purple), S^-Gh (green) and S^+Gh (yellow). Samples deemed outliers are indicated by a border. Brown vertical dashed line indicated separation of samples by *Spiroplasma* infection status. Lh= *L. heterotoma*, Gh= *Ganaspis sp*. S^-= *Spiroplasma*-free, S^+= *Spiroplasma* infected and W^- = Wasp-free.

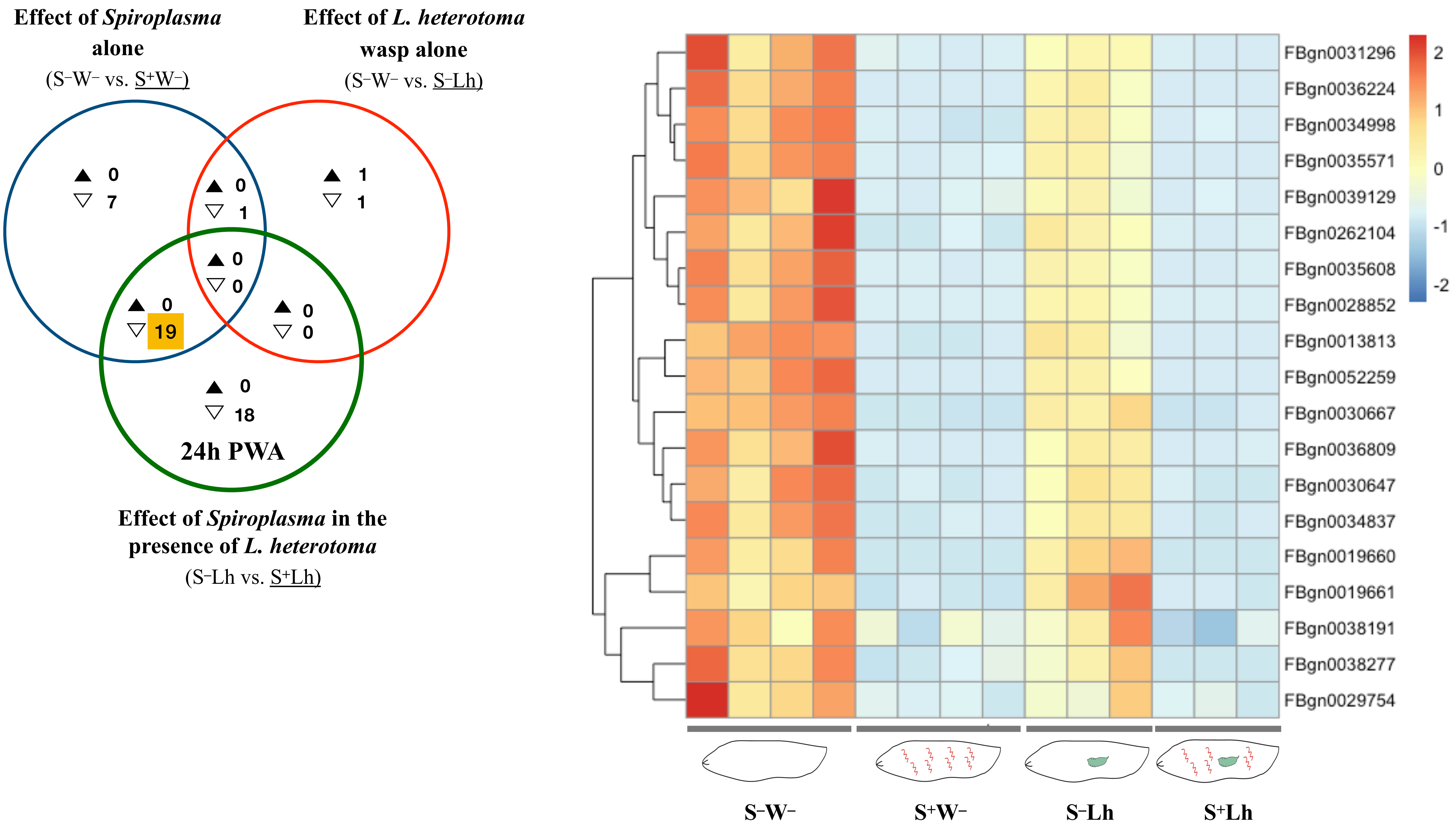

Figure S4. Analysis of *D. melanogaster* genes induced by *L. heterotoma* parasitism at Time point 1 (24h post-wasp attack, PWA). a) Venn diagram depicting significantly up- (black triangles) and down-regulated (white triangles) genes in the underlined treatment in three sets of pairwise comparisons among the relevant treatments; *Spiroplasma*-free & Wasp-free (S⁻W⁻), *Spiroplasma*-infected & Wasp-free (S⁺W⁻), *Spiroplasma*-free & *L. heterotoma* exposed (S⁻Lh), and *Spiroplasma*-infected & *L. heterotoma*-exposed (S⁺Lh). (B) Heatmap of the 19 downregulated genes (highlighted yellow in A) in the intersection between (S⁻W⁻ vs. S⁺W⁻) and (S⁻Lh vs. S⁺Lh). Expression levels per gene (row) are depicted as Z-scores (scaled by row), of the trimmed mean of M-values (TMM), for each replicate within each of the four treatments.

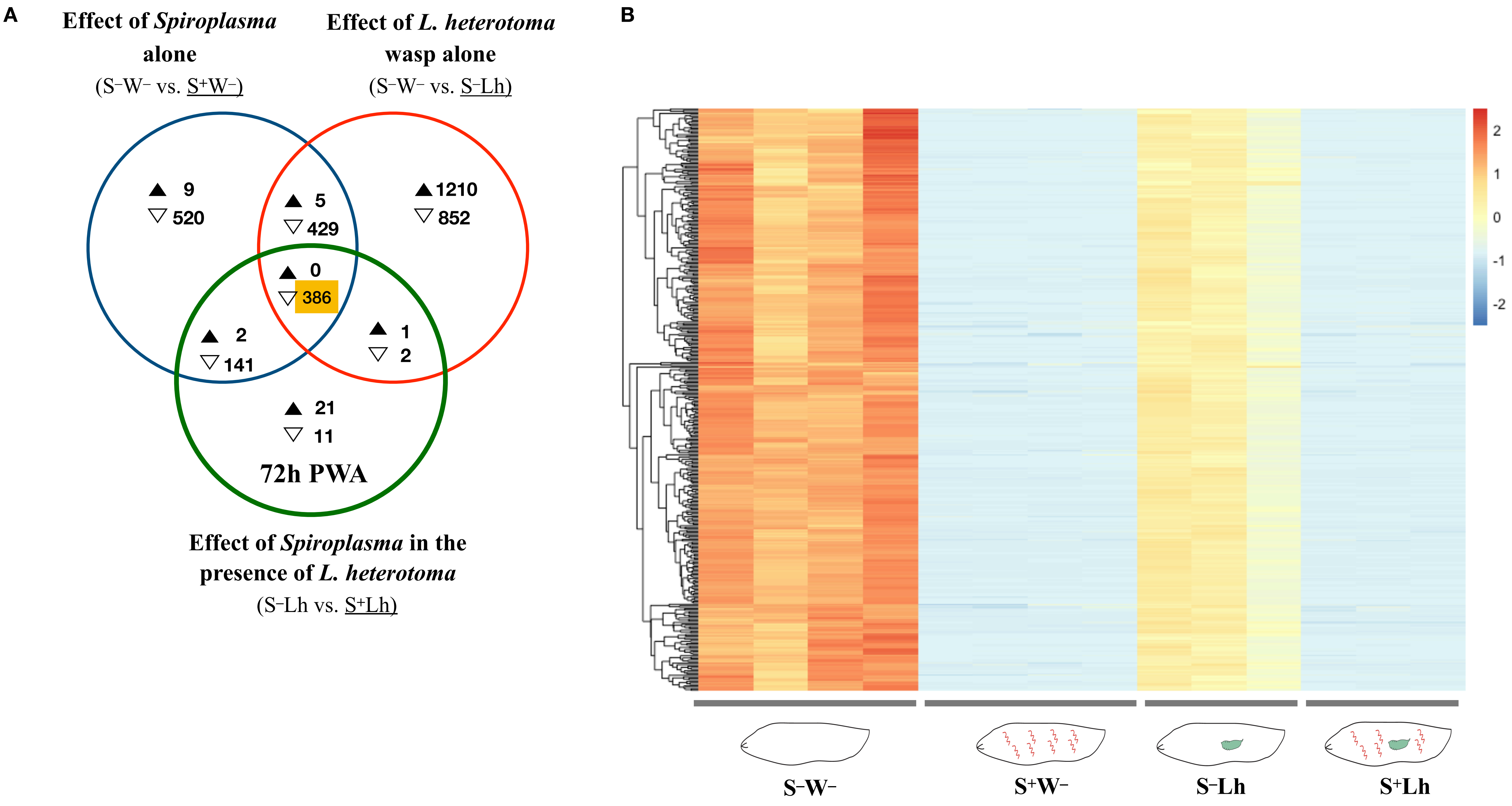

Figure S5. Analysis of *D. melanogaster* genes induced by *L. heterotoma* parasitism at Time point 2 (72h post-wasp attack, PWA). (A) Venn diagram depicting significantly up- (black triangles) and down-regulated (white triangles) genes in the underlined treatment in three sets of pairwise comparisons among the relevant treatments; *Spiroplasma*-free & Wasp-free (S^-W^-), *Spiroplasma*-infected & Wasp-free (S^+W^-), *Spiroplasma*-free & *L. heterotoma* exposed (S^-Lh), and *Spiroplasma*-infected & *L. heterotoma*-exposed (S^+Lh). (B) Heatmap of the 386 genes in the intersection of all comparisons (highlighted yellow in A). Expression levels per gene (row) are depicted as Z-scores (scaled by row), of the trimmed mean of M-values (TMM), for each replicate within each of the four treatments.

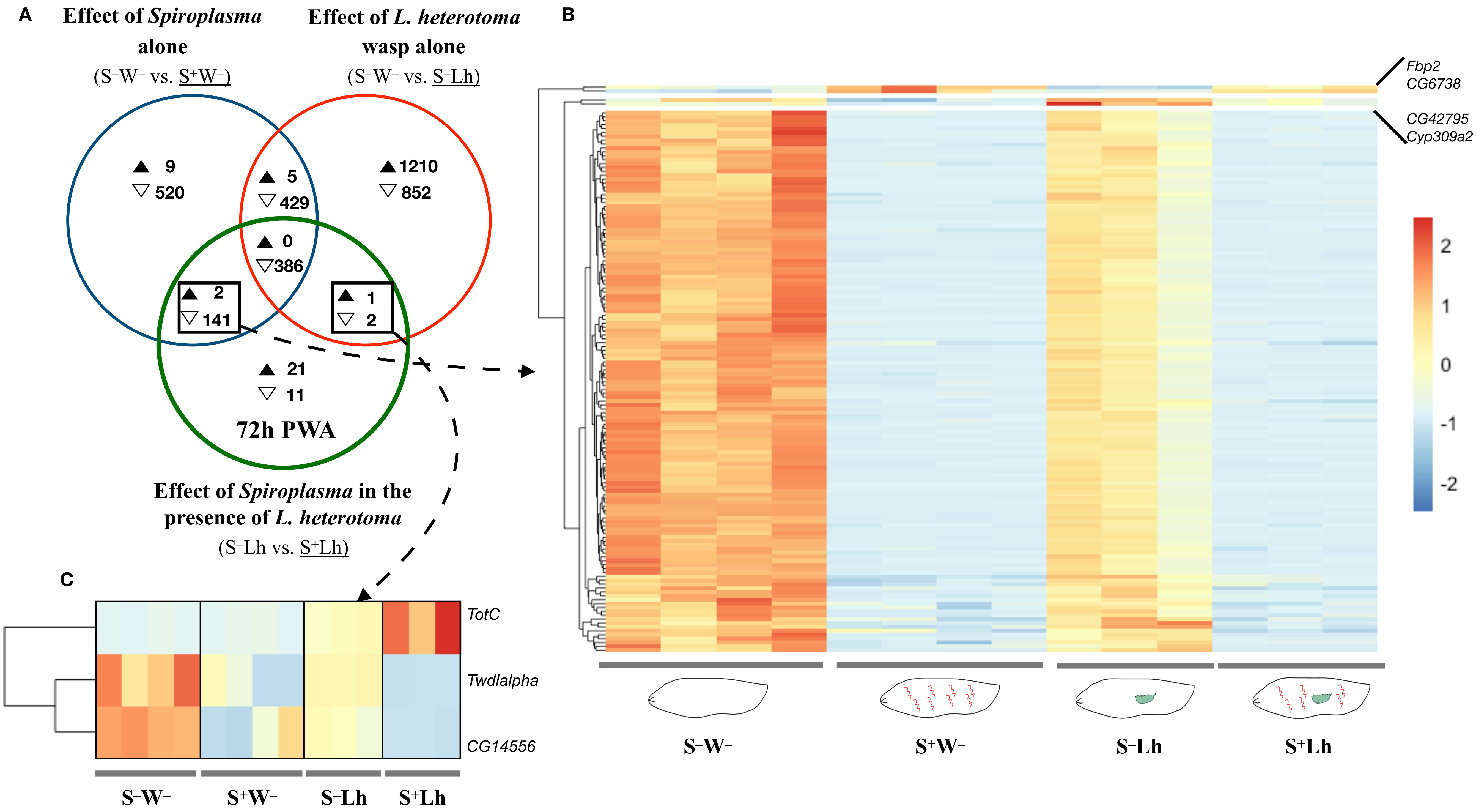

Figure S6. Analysis of *D. melanogaster* genes induced by *L. heterotoma* parasitism at Time point 2 (72h post-wasp attack, PWA). (A) Venn diagram depicting significantly up- (black triangles) and down-regulated (white triangles) genes in the underlined treatment in three sets of pairwise comparisons among the relevant treatments; *Spiroplasma*-free & Wasp-free (S−W−), *Spiroplasma*-infected & Wasp-free (S+W−), *Spiroplasma*-free & *L. heterotoma* exposed (S−Lh), and *Spiroplasma*-infected & *L. heterotoma*-exposed (S+Lh). (B) Heatmap of the 143 genes found in the intersection of the (S−Lh vs. S+Lh) and (S−W− vs. S+W−) sets. (C) Heatmap of the 3 genes in intersection of the (S−Lh vs. S+Lh) and (S−W− vs. S−Lh) sets. Genes *Fbp2* and *TotC* are also depicted in Fig. 3C. Expression levels per gene (row) are depicted as Z-scores (scaled by row), of the trimmed mean of M-values (TMM), for each replicate within each of the four treatments.

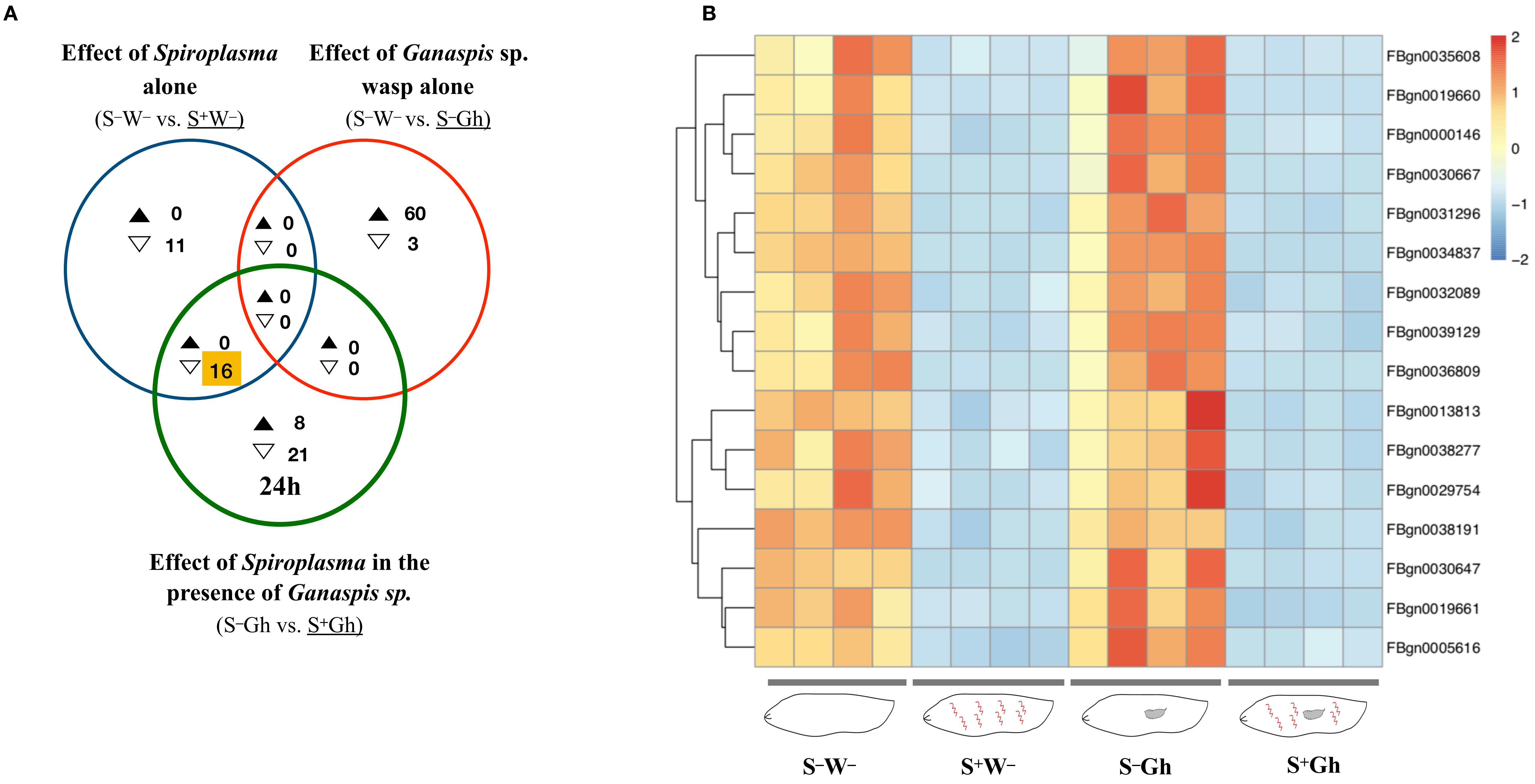

Figure S7. Analysis of *D. melanogaster* genes induced by *Ganaspis sp.* parasitism at Time point 1 (24h post-wasp attack, PWA). (A) Venn diagram depicting significantly up- (black triangles) and down-regulated (white triangles) genes in the underlined treatment in three sets of pairwise comparisons among the relevant treatments; *Spiroplasma*-free & Wasp-free (S^-W^-), *Spiroplasma*-infected & Wasp-free (S^+W^-), *Spiroplasma*-free & *Ganaspis sp.*-exposed (S^-Gh), and *Spiroplasma*-infected & *Ganaspis sp*-exposed (S^+Gh). (B) Heatmap of the 16 genes (highlighted yellow in A) from the intersection between (S^-Gh vs. S^+Gh) and (S^-W^- vs S^+W^-). Expression levels per gene (row) are depicted as Z-scores (scaled by row), of the trimmed mean of M-values (TMM), for each replicate within each of the four treatments.

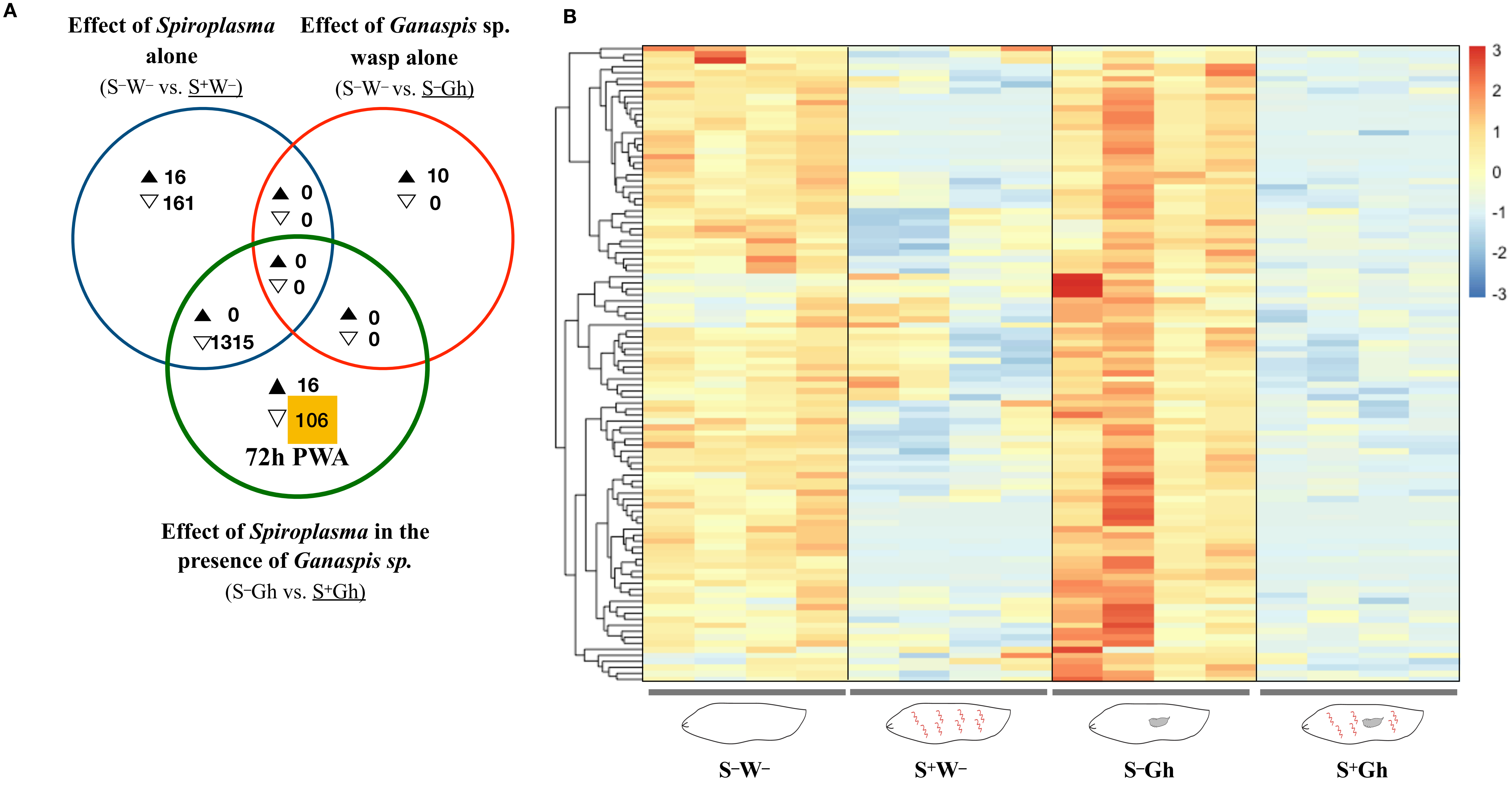

Figure S8. Analysis of *D. melanogaster* genes induced by *Ganaspis sp.* parasitism at Time point 2 (72h post-wasp attack, PWA). (A) Venn diagram depicting significantly up- (black triangles) and down-regulated (white triangles) genes in the underlined treatment in three sets of pairwise comparisons among the relevant treatments; *Spiroplasma*-free & Wasp-free (S−W−), *Spiroplasma*-infected & Wasp-free (S+W−), *Spiroplasma*-free & *Ganaspis sp.*-exposed (S−Gh), and *Spiroplasma*-infected & *Ganaspis sp*-exposed (S+Gh). (B) Heatmap of the 106 exclusively downregulated genes (highlighted in yellow in A) in the (S−Gh vs. S+Gh) comparison. Expression levels per gene (row) are depicted as Z-scores (scaled by row), of the trimmed mean of M-values (TMM), for each replicate within each of the four treatments.

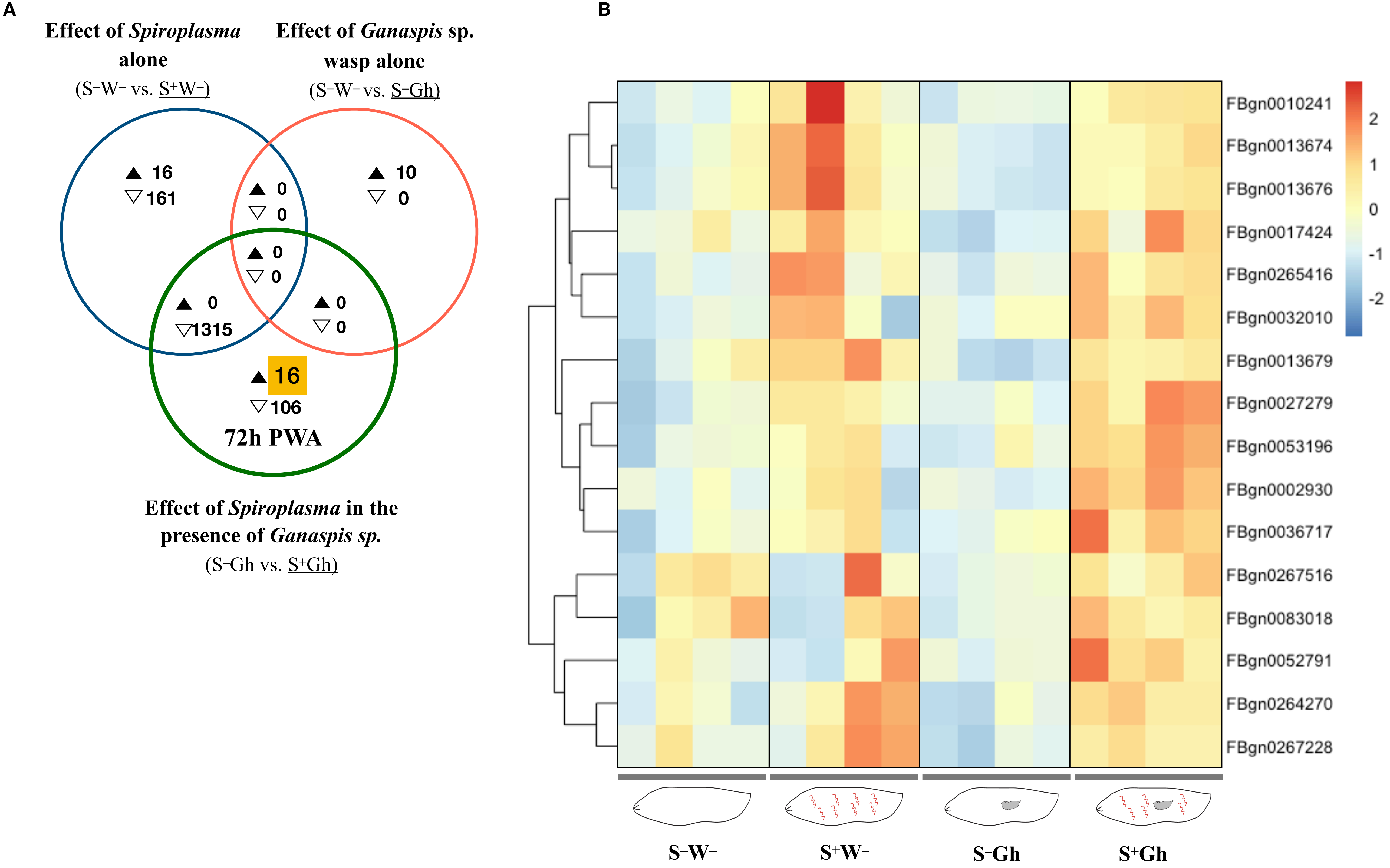

Figure S9. Analysis of *D. melanogaster* genes induced by *Ganaspis sp.* parasitism at Time point 2 (72h post-wasp attack, PWA). (A) Venn diagram depicting significantly up- (black triangles) and down-regulated (white triangles) genes in the underlined treatment in three sets of pairwise comparisons among the relevant treatments; *Spiroplasma*-free & Wasp-free (S^-W^-), *Spiroplasma*-infected & Wasp-free (S^+W^-), *Spiroplasma*-free & *Ganaspis sp.*-exposed (S^-Gh), and *Spiroplasma*-infected & *Ganaspis sp*-exposed (S^+Gh). (B) Heatmap of the 16 exclusively upregulated genes (highlighted in yellow in A) in the (S^-Gh vs. S^+Gh) comparison. Expression levels per gene (row) are depicted as Z-scores (scaled by row), of the trimmed mean of M-values (TMM), for each replicate within each of the four treatments.

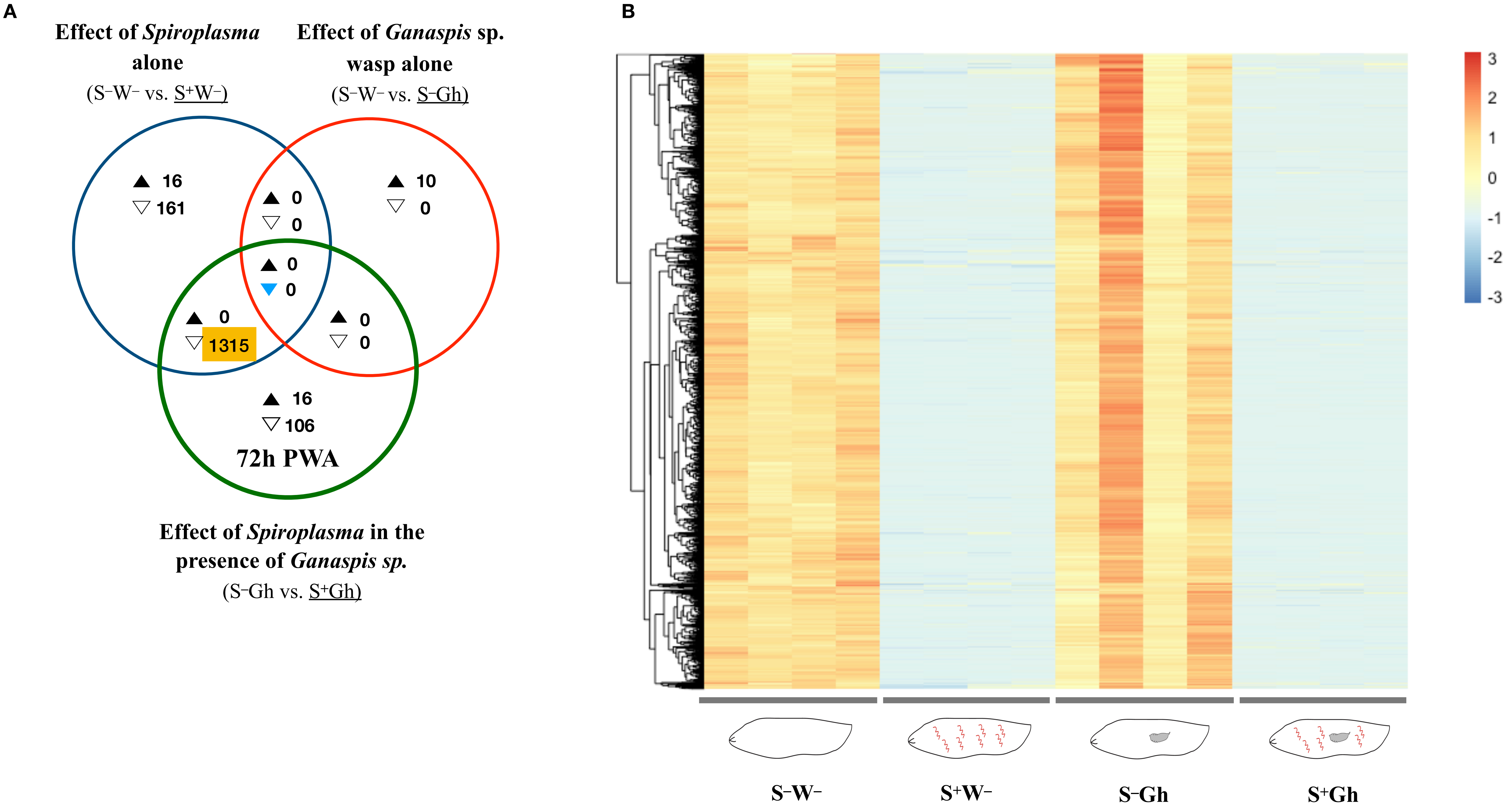

Figure S10. Analysis of *D. melanogaster* genes induced by *Ganaspis sp*. parasitism at Time point 2 (72h post-wasp attack, PWA). (A) Venn diagram depicting significantly up- (black triangles) and down-regulated (white triangles) genes in the underlined treatment in three sets of pairwise comparisons among the relevant treatments; *Spiroplasma*-free & Wasp-free (S-W-), *Spiroplasma*-infected & Wasp-free (S+W-), *Spiroplasma*-free & *Ganaspis sp.*-exposed (S-Gh), and *Spiroplasma*-infected & *Ganaspis sp*-exposed (S+Gh). (B) Heatmap of the 1315 genes (highlighted yellow in A) in the intersection between the (S-W- vs S+W-) and. (S-Gh vs S+Gh) sets. Expression levels per gene (row) are depicted as Z-scores (scaled by row), of the trimmed mean of M-values (TMM), for each replicate within each of the four treatments.

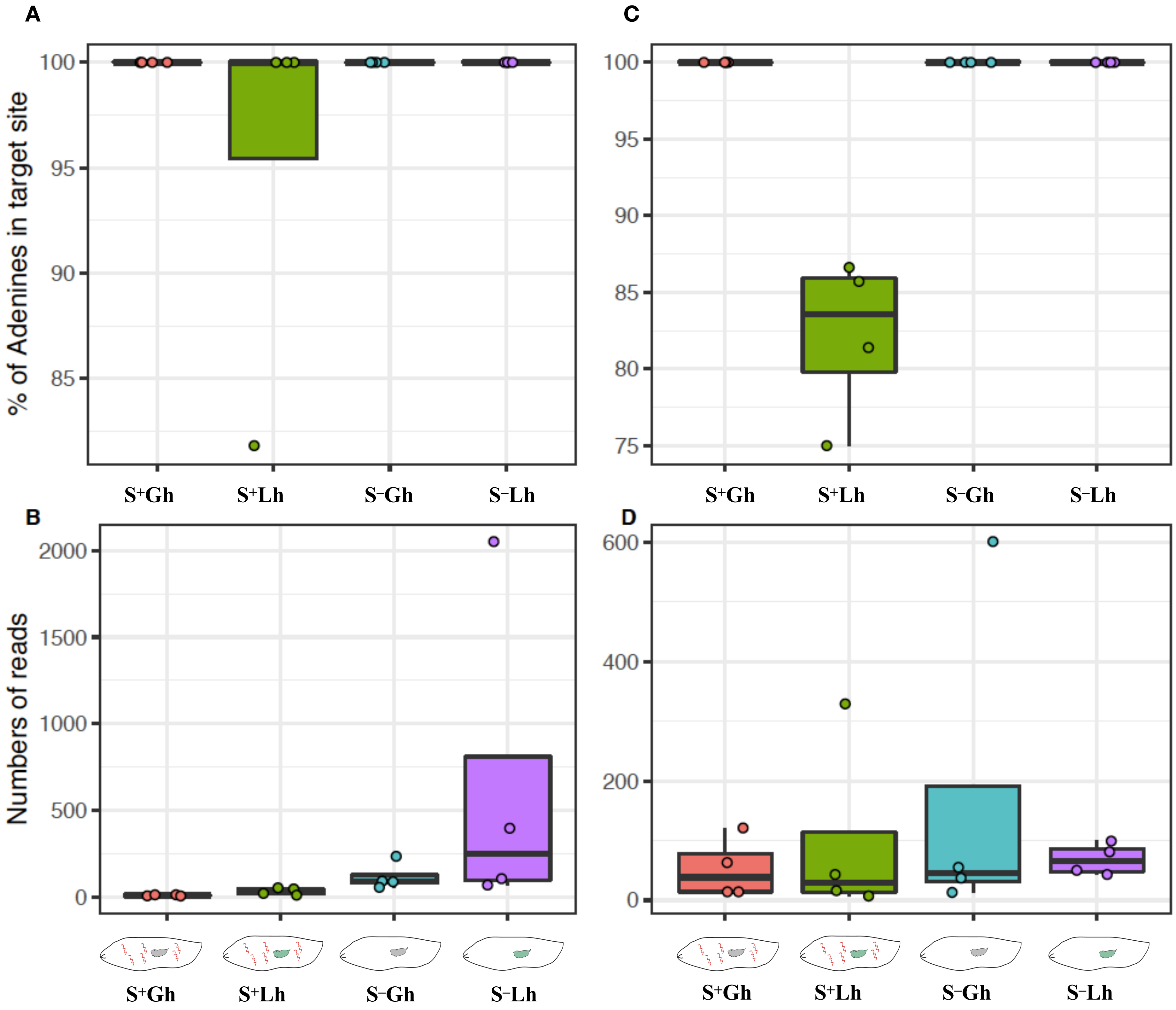

Figure S11. Signals of depurination in 28S rRNA of the wasps in following treatments: *Spiroplasma*-infected & *L. heterotoma*-exposed (S+Lh); *Spiroplasma*-infected & *Ganaspis* sp-exposed (S+Gh); *Spiroplasma*-free & *L. heterotoma*-exposed (S−Lh); and *Spiroplasma*-free & *Ganaspis* sp.-exposed (S−Gh). Percent of adenines (i.e., the expected base in the absence of depurination) at the target site of Ribosome Inactivating Proteins (RIPs), (A) and (C). Number of wasp reads that mapped to the target depurination sites (B) and (D). Time point 1 (T1; 24h PWA) is shown in (A) and (B); Time-point 2 (T2; 72h PWA) is shown in (C) and (D). In S+Lh treatment at T1, there is no significant effect of *Spiroplasma* on adenine proportion (X^2=0.10863, df=1, P=0.7417). In T2, a significant effect of treatment was detected (X^2=128.58, df=1,P< 2.2 e-16).

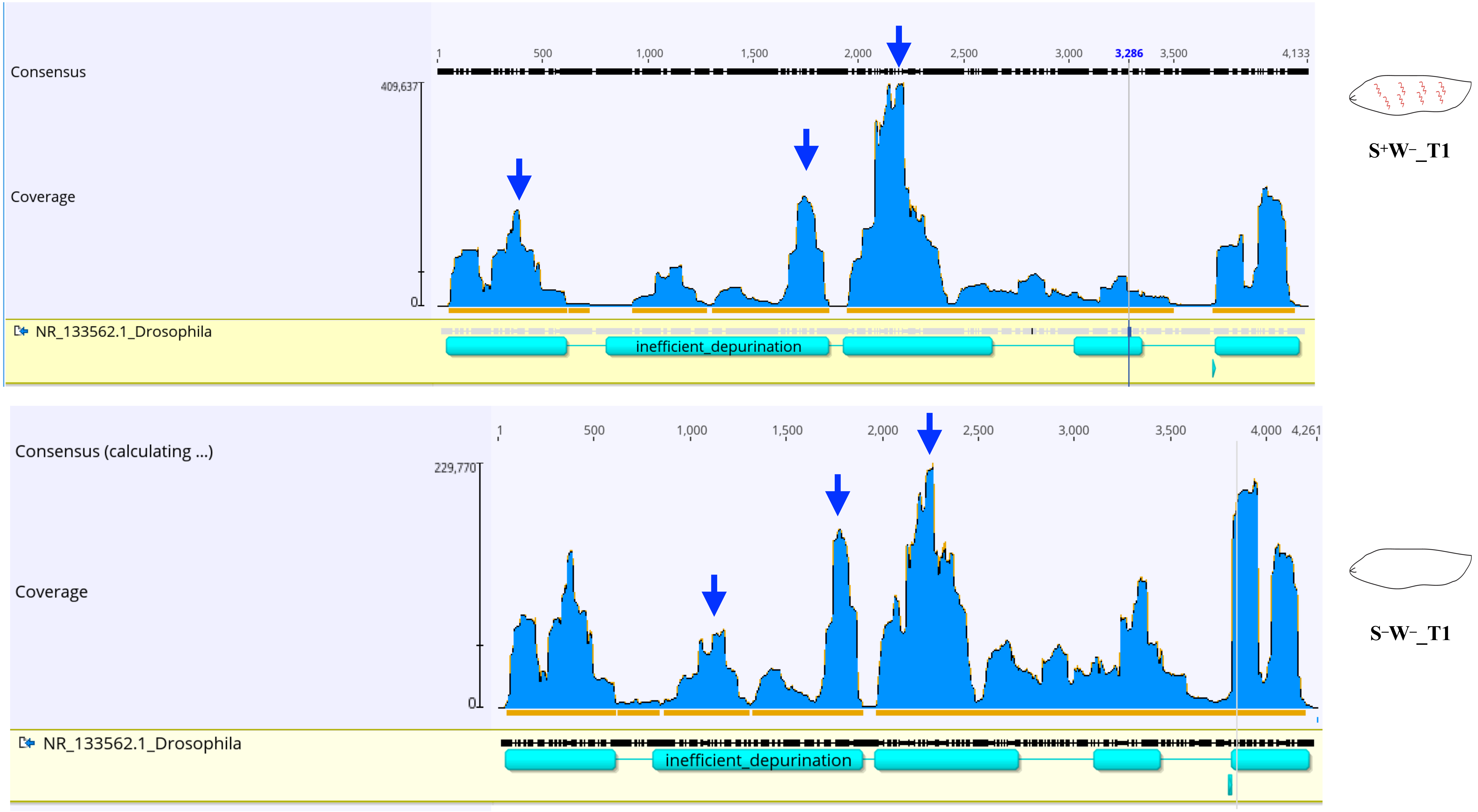

Figure S12. Non-uniform removal of *D. melanogaster* ribosomal RNA (ribodepletion). Images of the recovered reads from the transcriptome that aligned to the full sequence of the 28S rRNA of *D. melanogaster*. The following treatments were evaluated: *Spiroplasma*-free & Wasp-free (S−W−); and *Spiroplasma*-infected & Wasp-free (S+W−). The images are derived from visualizing mapped reads in Geneious software from one replicate per treatment at Time point 1 (T1). Blue arrows and aquamarine rectangles indicate some ribosomal regions high coverage, and thus less efficient ribodepletion.

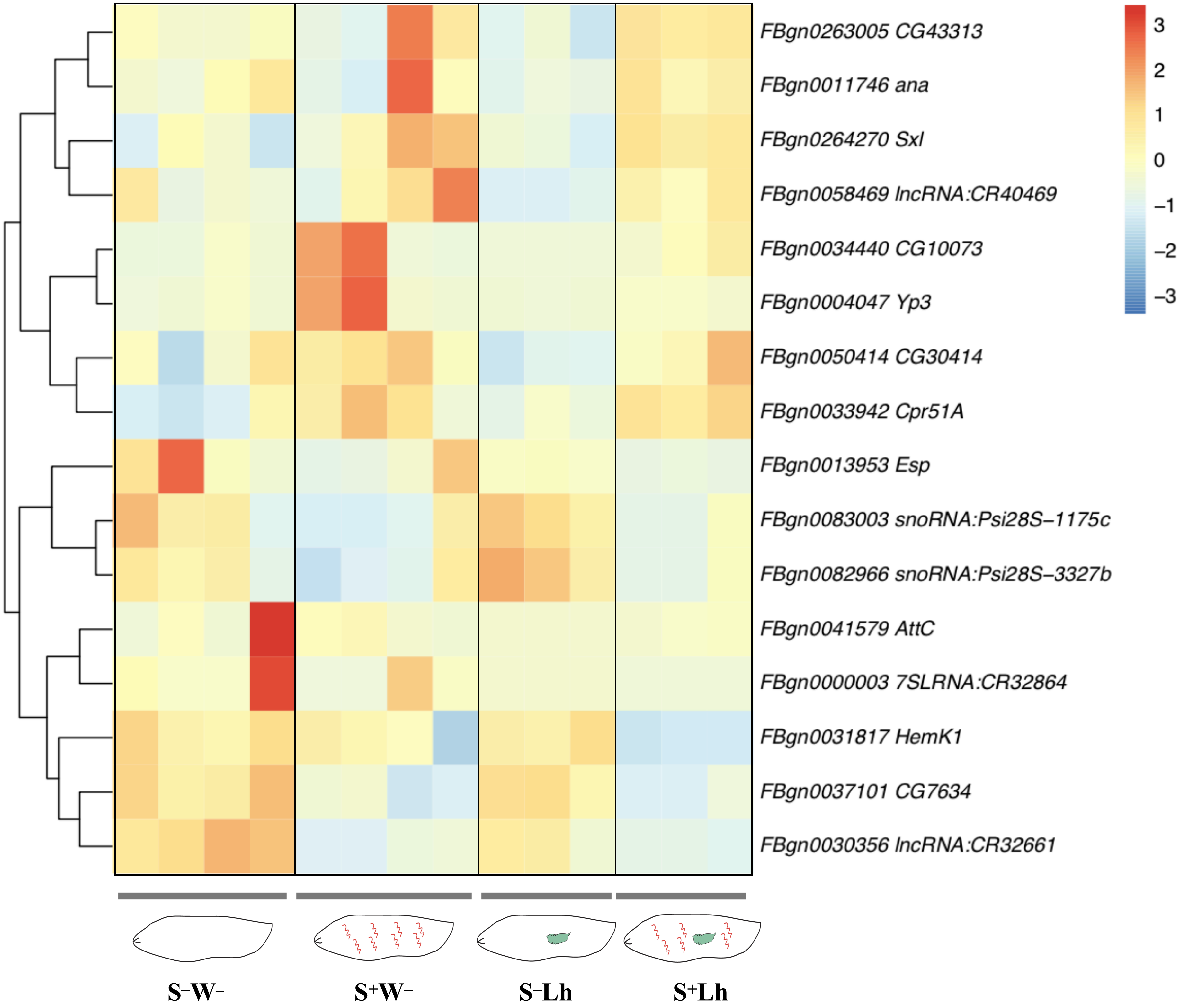

Figure S13. Analysis of *D. melanogaster* genes to complement those in Fig. 3B. Shown here are heatmaps of 9 up- and 7 down-regulated genes from the exclusive green set (S^-Lh vs. S^+Lh comparison; Fig. 3B) that do not belong to Group A–C at Time point 2 (72h post-wasp attack, PWA). Expression levels per gene (row) are depicted as Z-scores (scaled by row), of the trimmed mean of M-values (TMM), for each replicate within each of the four treatments. *Spiroplasma*-free & Wasp-free (S^-W^-), *Spiroplasma*-infected & Wasp-free (S^+W^-), *Spiroplasma*-free & *L. heterotoma* exposed (S^-Lh), and *Spiroplasma*-infected & *L. heterotoma*-exposed (S^+Lh).

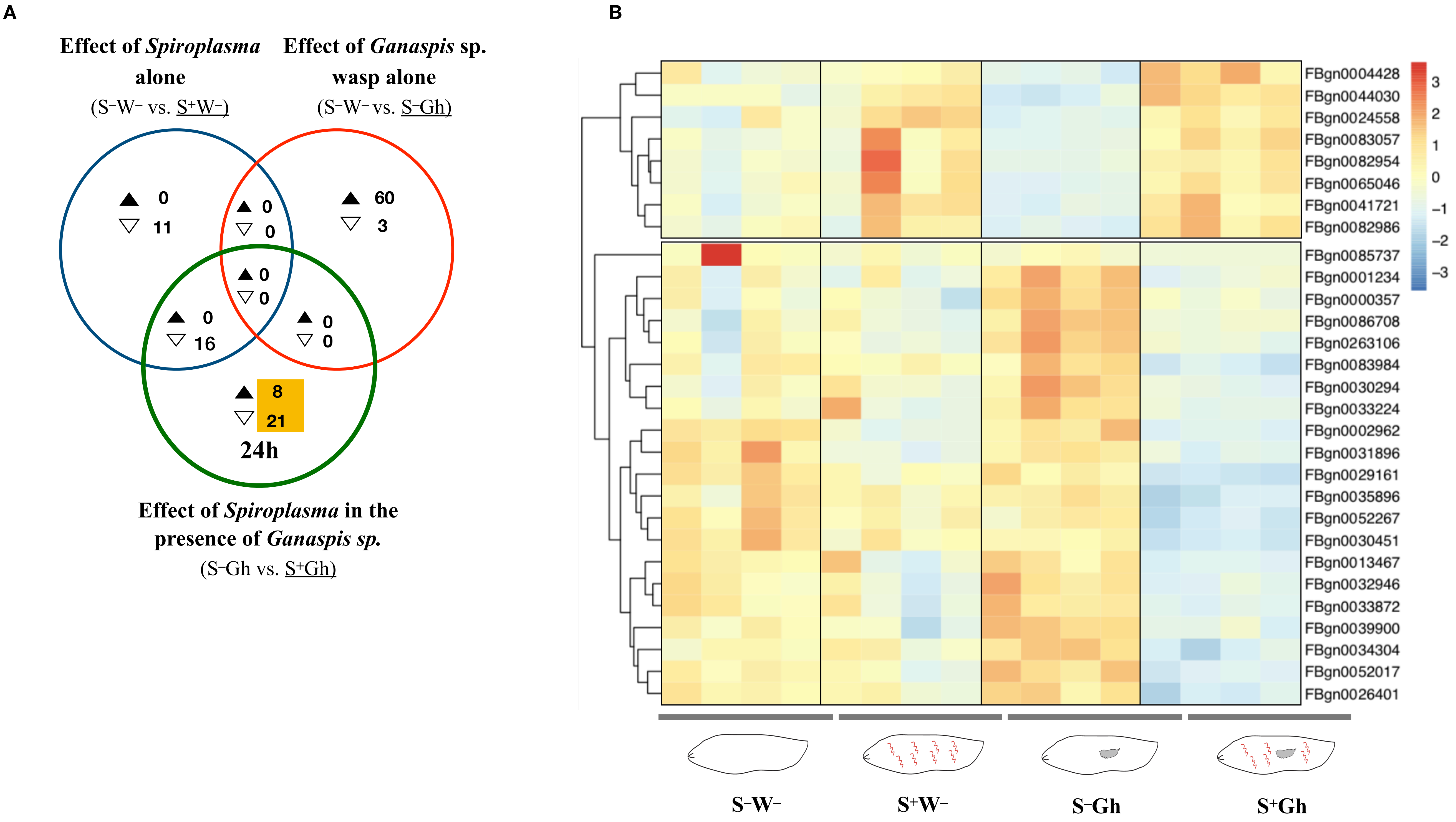

Figure S14. Analysis of *D. melanogaster* genes induced by *Ganaspis sp.* parasitism at Time point 1 (24h post-wasp attack, PWA). (A) Venn diagram depicting significantly up- (black triangles) and down-regulated (white triangles) genes in the underlined treatment in three sets of pairwise comparisons among the relevant treatments; *Spiroplasma*-free & Wasp-free (S-W-), *Spiroplasma*-infected & Wasp-free (S+W-), *Spiroplasma*-free & *Ganaspis sp.*-exposed (S-Gh), and *Spiroplasma*-infected & *Ganaspis sp*-exposed (S+Gh). (B) Heatmap of the 8 + 21 exclusive genes (highlighted yellow in A) in the (S-Gh vs S+Gh) comparison. Expression levels per gene (row) are depicted as Z-scores (scaled by row), of the trimmed mean of M- values (TMM), for each replicate within each of the four treatments.
